# Supplementary material for: Homogeneous solution assembled Turing structures with near zero strain semi-coherence interface
Source: Nat Commun. 2022 May 26;13:2942. doi: 10.1038/s41467-022-30574-3 (PMC9135718; doi:10.1038/s41467-022-30574-3)
Supplement: Supplementary file 1 — Supplementary Information [file 41467_2022_30574_MOESM1_ESM.pdf]

Supplementary Information

Homogeneous solution assembled Turing structures with near zero strain semi-coherence interface

Yuanming Zhang[1, 2]; Ningsi Zhang[1, 2]; Yong Liu[3]; Yong Chen[1, 2]; Huiting Huang[1, 2]; Wenjing Wang[1, 2]; Xiaoming Xu[1, 2]; Yang Li[1, 2]; Fengtao Fan[3]; Jinhua Ye[4]; Zhaosheng Li[1, 2*]; Zhigang Zou[1, 2]

[1]Collaborative Innovation Center of Advanced Microstructures, National Laboratory of Solid State Microstructures, College of Engineering and Applied Sciences, Nanjing University, 22 Hankou Road, Nanjing 210093, China.

[2]Jiangsu Key Laboratory of Nano Technology, Nanjing University, 22 Hankou Road, Nanjing 210093, China.

[3]State Key Laboratory of Catalysis, Dalian Institute of Chemical Physics, Chinese Academy of Sciences, Dalian National Laboratory for Clean Energy, Dalian 116023, China.

[4]International Center for Materials Nanoarchitectonics (WPI-MANA), National Institute for Materials Science, 1-1 Namiki, Tsukuba 305-0047, Japan.

*Corresponding Author E-mail: zsli@nju.edu.cn

Table of contents:

Supplementary Fig. 1. The relationship between photoelectrode performance (AM 1.5G, 100 mW cm^{-2}) and lattice constant and ionic radius……………………………………………………………………………………4

Supplementary Fig. 2. The calculated electronic structure and partial density of states around Fermi level……..5

Supplementary Fig. 3. Schematic diagrams of several representative photoelectrode with heterojunction structure……..7

Supplementary Fig. 4. SEM images and elemental mapping of conventional dual-phase interface films by electrophoretic deposition……………………………………………………………………………………..8

Supplementary Fig. 5. Side-view SEM images, elemental mapping and TEM images of conventional dual-phase interface film……………………………………………………………………………………………9

Supplementary Fig. 6. SEM images and XRD patterns of non-Turing dual-phase interface films (Zn: Fe=1:3 and Zn: Fe=1:4) prepared from inorganic metal source……………………………………………………...10
Supplementary Fig. 7. Preparation of APT tip samples………………………………………………………11
Supplementary Fig. 8. APT test of the Turing interface film (Zn: Fe=1:3)……………………………………12
Supplementary Fig. 9. APT test of the $ZnFe_2O_4$ film…………………………………………………………..13
Supplementary Fig. 10. Charge separation efficiency of the photoelectrodes…………………………………14
Supplementary Fig. 11. Schematic diagram of spatial structure and Rietveld refinement of XRD patterns…15
Supplementary Fig. 12. XPS data……………………………………………………………………………..16
Supplementary Fig. 13. High-resolution XPS signals of Zn 2*p*, Fe 2*p*, O 1*s* and Sn 3*d*……………………….17
Supplementary Fig. 14. Understanding of re-coordination mechanism based on Tyndall effect……………..18
Supplementary Fig. 15. Ratio of diffusion coefficient…………………………………………………………20
Supplementary Fig. 16. SEM images of Turing structure formation process…………………………………21
Supplementary Fig. 17. SEM images, elemental mapping and HAADF-STEM images of Turing structure film………22
Supplementary Fig. 18. Side-view SEM images and elemental mapping………………………………………23
Supplementary Note 1. Numerical Simulation…………………………………………………………………..24
Supplementary Fig. 19. Numerical simulation…………………………………………………………………...27
Supplementary Fig. 20. Optical observation of shape evolution of the droplets………………………………28
Supplementary Fig. 21. *In situ* optical observation of shape evolution of the droplets………………………..29
Supplementary Fig. 22. Schematic illustration for the preparation of photoelectrode films…………………30
Supplementary Fig. 23. HAADF-STEM images…………………………………………………………………31
Supplementary Fig. 24. HAADF-STEM images…………………………………………………………………32
Supplementary Fig. 25. Composition analysis…………………………………………………………………..33
Supplementary Fig. 26. Elemental mappings……………………………………………………………………34
Supplementary Fig. 27. Structural analysis……………………………………………………………………...35
Supplementary Fig. 28. HAADF-STEM images…………………………………………………………………36
Supplementary Fig. 29. Crystal structure………………………………………………………………………..37
Supplementary Fig. 30. Photoelectrochemical activities for the Turing interface films……………………….38
Supplementary Fig. 31. Photoelectrochemical activities under LED illumination……………………………..39
Supplementary Fig. 32. Faraday efficiency………………………………………………………………………40
Supplementary Fig. 33. Photoelectrocatalytic process for the removal of phenol………………………………41
Supplementary Fig. 34. Photoelectrochemical activities for the conventional dual-phase interface films…...42
Supplementary Fig. 35. The relationship between photocurrent and α-Fe_2O_3 content and film thickness…43
Supplementary Fig. 36. Band edge position……………………………………………………………………44
Supplementary Fig. 37. Selective photodeposition………………………………………………………………45
Supplementary Fig. 38. Interface states…………………………………………………………………………46
Supplementary Fig. 39. Optical properties of thin films…………………………………………………………..47
Supplementary Fig. 40. Photoelectrochemical impedance………………………………………………………48
Supplementary Fig. 41. Photoelectrochemical activities…………………………………………………………49
Supplementary Fig. 42. Photoelectrochemical wastewater treatment…………………………………………..50
Supplementary Fig. 43. SPV and TPV…………………………………………………………………………51
Supplementary Fig. 44. KPFM and C-AFM……………………………………………………………………...52
Supplementary Fig. 45. PL and TRPL…………………………………………………………………………53
Supplementary Fig. 46. AFM micrographs (2D and 3D) prepared from $Fe[C_5H_7O_2]_3$………………………...54

Supplementary Fig. 47. AFM micrographs (2D and 3D) of non-Turing dual-phase interface films (Zn: Fe=1:3 and Zn: Fe=1:4) prepared from inorganic metal source……………………………………………………..55
Supplementary Fig. 48. Different fabrication strategies were used to verify the homogeneous solution characteristics of Turing structure and non-Turing structure…………………………………………………56
Supplementary Fig. 49. The shape evolution of droplets with different solvents………………………………57
Supplementary Fig. 50. Elemental mappings……………………………………………………………………59
Supplementary Fig. 51. SEM images, elemental mappings and photoelectrochemical activities of Zn-doped α-Fe_2O_3 films……………………………………………………………………………………………………60
Supplementary Fig. 52. XRD patterns, SEM images and elemental mappings…………………………………61
Supplementary Fig. 53. Optical properties of the samples………………………………………………………62
Supplementary Fig. 54. XPS spectra and band edge position of the samples…………………………………..63
Supplementary Fig. 55. SEM images and elemental mappings of Turing interface film………………………64
Supplementary Fig. 56. Curves of relationship between photocurrents at 1.6 V vs. RHE and α-Fe_2O_3 content……..65
Supplementary Table 1. Comparison of our films to other iron-based films in recent years…………………66
Supplementary Table 2. Quality of raw materials used in the preparation of the conventional dual-phase interface films……………………………………………………………………………………………………...67
Supplementary Table 3. Solution concentrations for diffusion coefficient test…………………………………68
Supplementary Table 4. Photoelectrochemical impedance spectroscopic curves……………………………...69
Supplementary References……………………………………………………………………………………..70

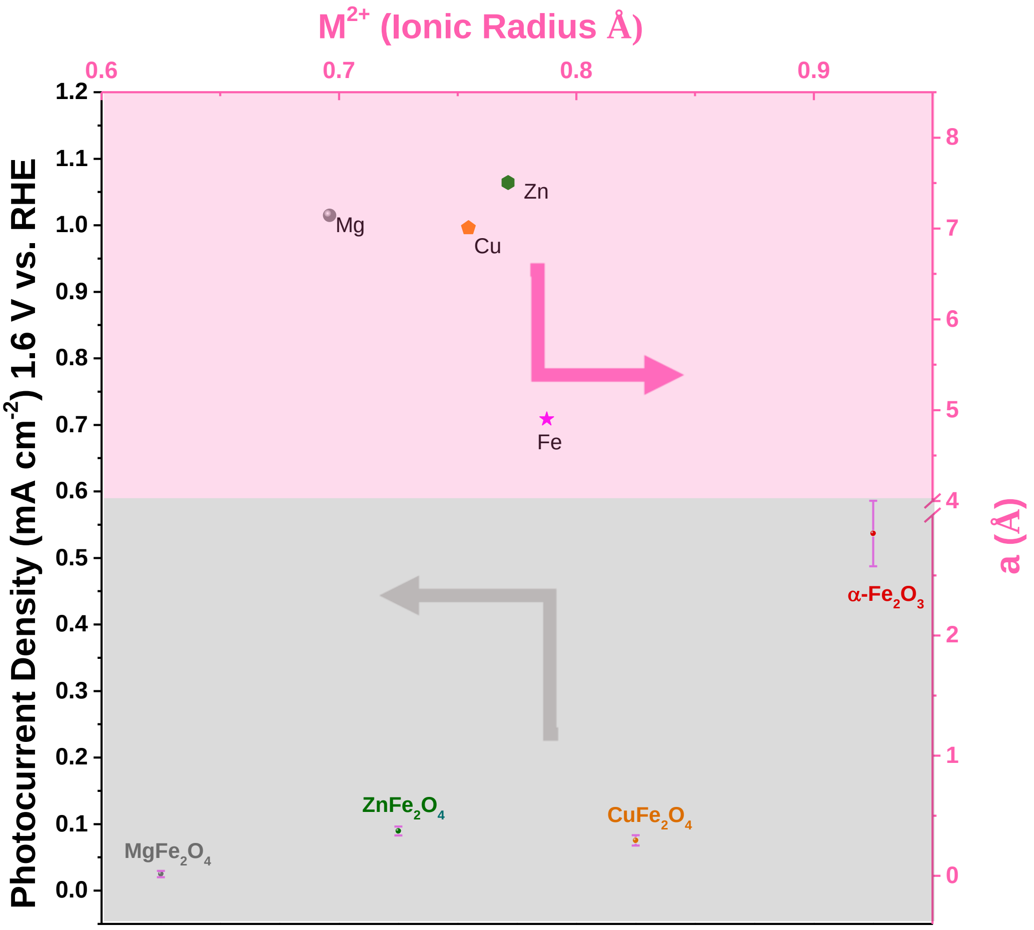

Supplementary Fig. 1. The relationship between photoelectrode performance (AM 1.5G, 100 mW cm^{-2}) and lattice constant and ionic radius. "a" represents one of the cell parameters of $ZnFe_2O_4$, $CuFe_2O_4$, $MgFe_2O_4$ and α-Fe_2O_3.

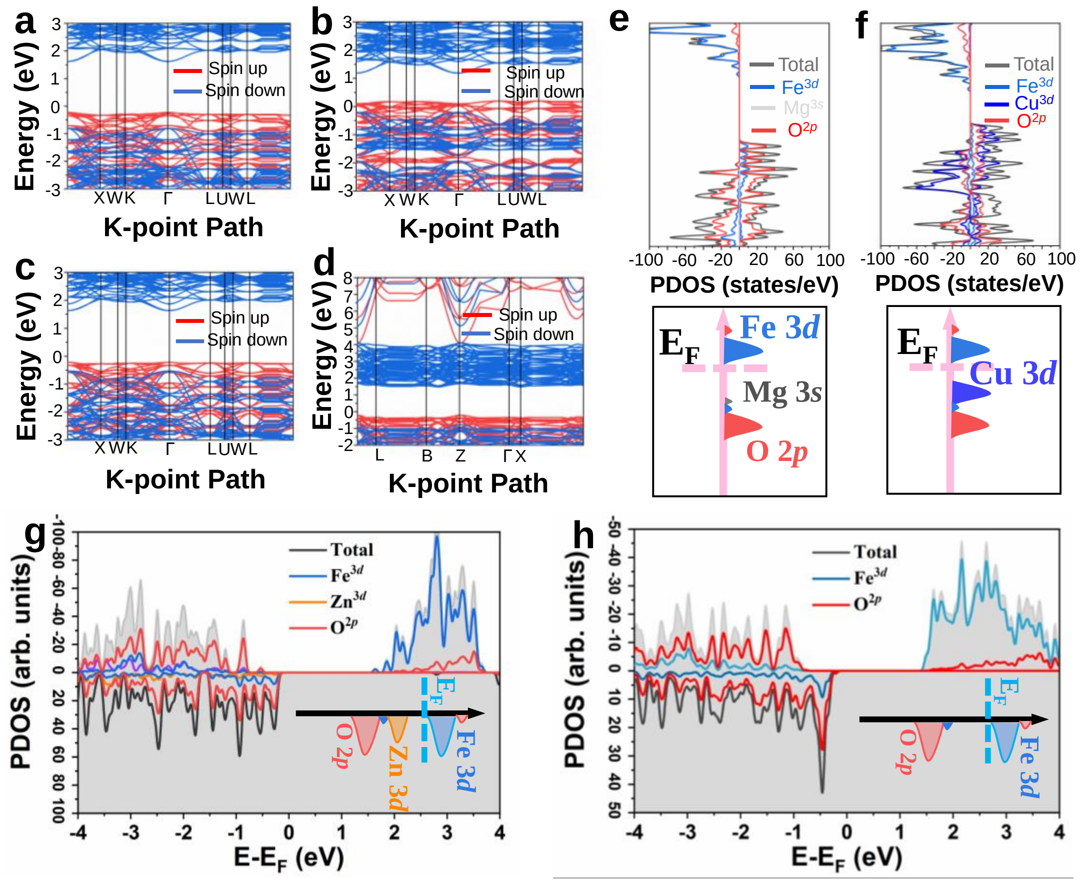

Supplementary Fig. 2. The calculated electronic structure and partial density of states around Fermi level. a, **e**, $MgFe_2O_4$. **b**, **f**, $CuFe_2O_4$. **c**, **g**, $ZnFe_2O_4$. **d**, **h**, α-Fe_2O_3.

Today, the global economy significantly depends on the use of fossil fuels, which entail excessive energy consumption. Renewable energy (i.e., solar, water, wind, wave, ocean thermal, wind, biomass and geothermal) is considered to be the most promising candidate to replace fossil fuel[1]. Moreover, the vision of peak carbon and carbon neutrality is also a global concern. Therefore, the development of renewable energy is an international priority. Inexhaustible and non-polluting solar energy has been attracting the attention of research scientists, and it has been conclusively shown that all of the energy consumed by humans in an entire year is less than the energy of an hour of sunlight radiating the Earth. Although solar energy is available through photovoltaics combined with electrolyser technology, it is not economical due to the high costs of system components.

Photoelectrochemical (PEC) tandem cells have attracted intensive interest during the past decades as an appealing technology at lower cost and higher efficiency because

light absorption and target product generation are integrated on the semiconductor[2–5]. However, the cost, intrinsic properties of semiconductors and lack of large-scale preparation technology are the primary factors restricting the development of PEC. Semiconductors with natural abundance, chemical stability, non-toxicity, high resistance to photo-corrosion, and low production costs are the most suitable candidates among all the investigated semiconductors[6–8]. Iron-based oxide semiconductors stand out and have recently been widely studied[9–16]. In addition to the small bandgaps, the stability in alkaline and weakly acidic media, has motivated the study of spinel ferrites such as ternary metal oxides (MFe_2O_4, M=Mg, Ca, Cu, Zn, etc. bandgap energy, E_g = 1.4 – 2.1 eV)[17]. The oxide ions are arranged in a cubic close-packed lattice and the cations M and Fe occupy some or all of the octahedral and tetrahedral sites in the spinel ferrites. The charge of M is +2, and the charge of Fe is +3, but the existence of other combinations is not ruled out[18]. The above composition can realize the compatibility of structural stability and defect-tolerance.

However, the performance of spinel ferrites films in light-driven water oxidation has remained poor compared with traditional semiconductors (Supplementary Fig. 1, α-Fe_2O_3 > $ZnFe_2O_4$> $CuFe_2O_4$> $MgFe_2O_4$), and the ionic radius and lattice constant have no strong relationship with the performance unlike some reports in other field[19] Therefore, density functional theory (DFT) calculations (Supplementary Fig. 2) were performed to understand the electronic structures. Oxygen ions principally determine the valence band, while Fe ions determine the conduction band, with a small contribution of the oxygen ions for α-Fe_2O_3. Taking $ZnFe_2O_4$ as an example, Zn states from −1 eV (the value of the Fermi level has been translated to zero) in valence band, while in the conduction band, the Zn state contribution is almost negligible. Therefore, based on a comparison with the contribution of M^{2+} to the valence band, controlling the saturation photocurrents of these materials by introducing heteroatoms is difficult due to the dominant photogenerated carrier bulk recombination[20]. The previous heterojunction strategy can enhance the separation efficiency of photogenerated carriers, but it is limited by development due to the cumbersome fabrication and ambiguous interface.

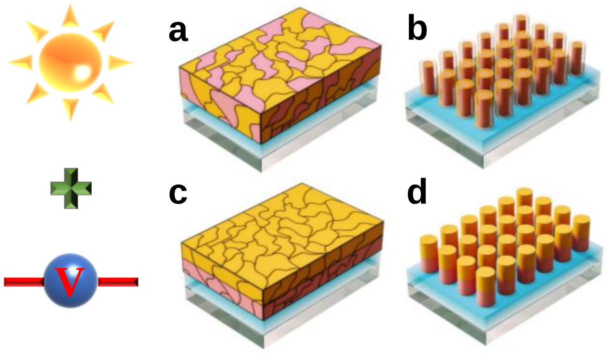

Supplementary Fig. 3. Schematic diagrams of several representative photoelectrode with heterojunction structure. **a**, Hybrid planar heterojunction. **b**, Nanoarray heterojunction with core-shell. **c**, Longitudinal hybrid heterojunction. **d**, Longitudinal nanoarray heterojunction.

Common manufacturing technology of a photoelectrode film includes spin-coating, electrodeposition, photoelectron deposition, electrophoresis, screen printing, hydrothermal, sol-gel methods, and particle transfer methods, which can lead to porous or nanostructures heterojunction photoelectrodes[21] (Supplementary Fig. 3). Taking the hydrothermal method as an example, the nanoarray core-shell heterojunction and longitudinal nanoarray heterojunction (Supplementary Fig. 3b and d) usually require two or more semiconductors to be prepared separately[22], so it usually requires multi-step operation, resulting in poor repeatability, which outweigh its advantages. Due to the scattering effect inside the sample, the irradiation of the sample may be non-uniform, and the charge effect will affect the measurement of the plate potential and space charge width because of a large amount of bulk defect and surface states. The complex atomic layer deposition and other preparation techniques were used to construct the laminated heterojunction film (Supplementary Fig. 3c), which also exhibits the above shortcomings. Based on the above considerations, we are inspired by the Turing structure to try to construct it (Supplementary Fig. 3a) on the photoelectrode film. Our focus is on the influence of the Turing interface formed by this structure on its photoelectrochemical performance. Particularly, the spinel ferrites as ternary metal oxides with different proportions of M and Fe can be prepared by a simple spray pyrolysis system, then the subsequent annealing process will directly lead to the formation of MFe_2O_4 and α-Fe_2O_3 interfaces.

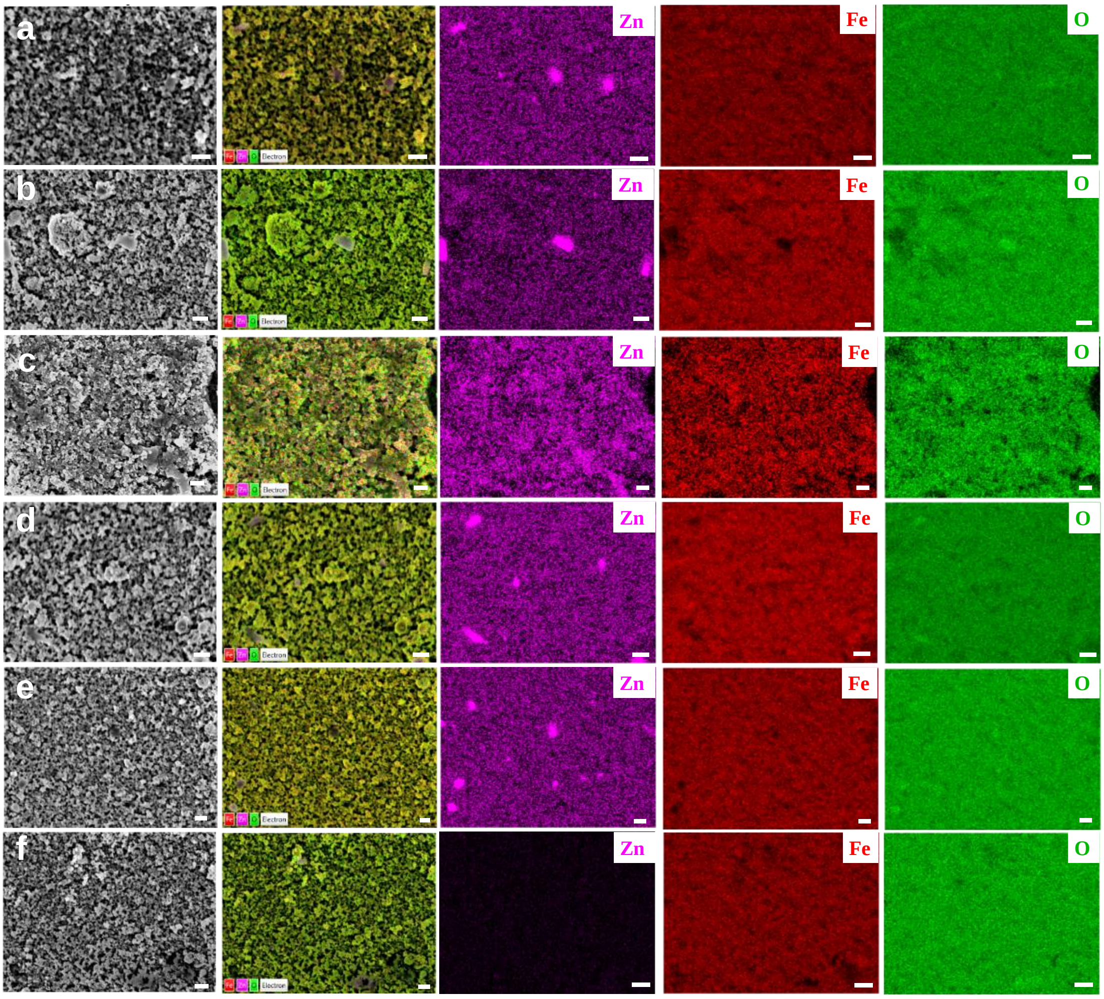

Supplementary Fig. 4. SEM images and elemental mapping of conventional dual-phase interface films by electrophoretic deposition. a, $ZnFe_2O_4$ film. **b**, Zn: Fe=1:2.4 film. **c**, Zn: Fe=1:3 film. **d**, Zn: Fe=1:4 film. **e**, Zn: Fe=1:6 film. **f**, α-Fe_2O_3 film. Scale bars: 500 nm.

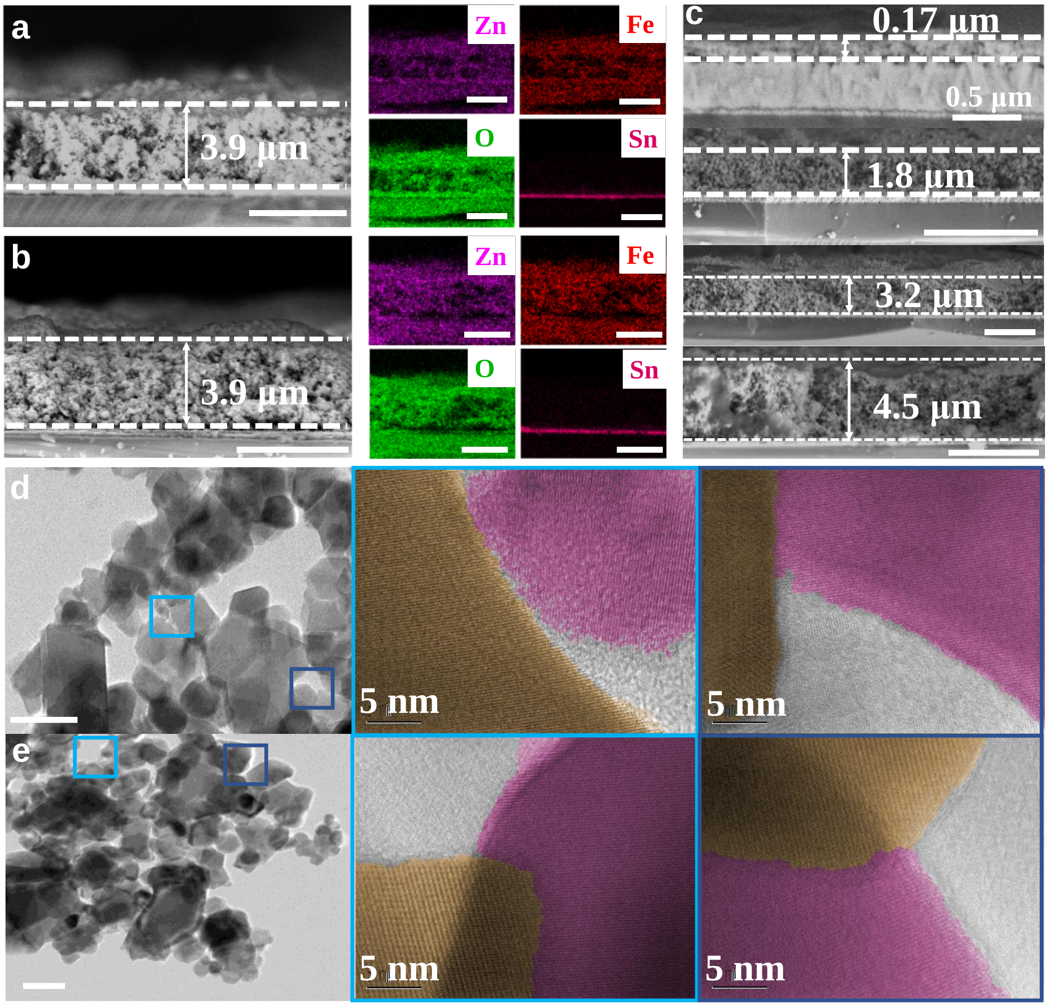

Supplementary Fig. 5. Side-view SEM images, elemental mapping and TEM images of conventional dual-phase interface film. **a**, Side-view SEM images and elemental mapping of Zn: Fe=1:3 film. **b**, Side-view SEM images and elemental mapping of Zn: Fe=1:4 film. **c**, The film thickness by changing the deposition time. **d**, TEM image and HR-TEM image of Zn: Fe=1:3 film (Scrape it off from the conventional dual-phase interface film by electrophoretic deposition, $n_{ZnFe_2O_4}$: $n_{\alpha\text{-}Fe_2O_3}$=2:1). **e**, TEM image and HR-TEM image of Zn: Fe=1:4 film (Scrape it off from the conventional dual-phase interface film by electrophoretic deposition, $n_{ZnFe_2O_4}$: $n_{\alpha\text{-}Fe_2O_3}$=1:1). $ZnFe_2O_4$ is yellow, while α-Fe_2O_3 is pink. Scale bars: **a**, **b**, **c**, 5 µm; **d**, **e**, 20 nm.

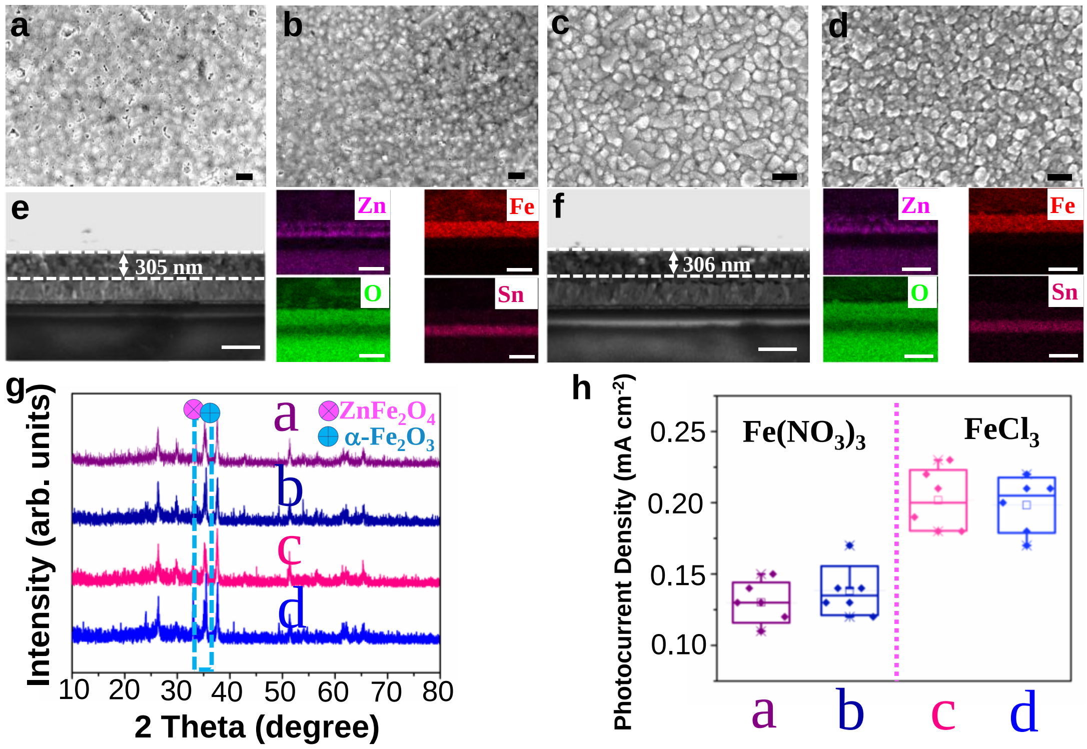

Supplementary Fig. 6. SEM images and XRD patterns of non-Turing dual-phase interface films (Zn: Fe=1:3 and Zn: Fe=1:4) prepared from inorganic metal source. **a**, **b**, $Fe(NO_3)_3$. **c**, **d**, $FeCl_3$. **e**, Side-view SEM images and elemental mapping of Zn: Fe=1:3 film prepared by $Fe(NO_3)_3$. **f**, Side-view SEM images and elemental mapping of Zn: Fe=1:3 film prepared by $FeCl_3$. **g**, XRD patterns for **a**, **b**, **c** and **d**. **h**, Photocurrent density (1.6 V vs. RHE, AM 1.5G, 100 mW cm^{-2}) for **a**, **b**, **c** and **d**. Scale bars: **a**, **b**, **c**, **d**, 100 nm; **e**, **f**, 500 nm.

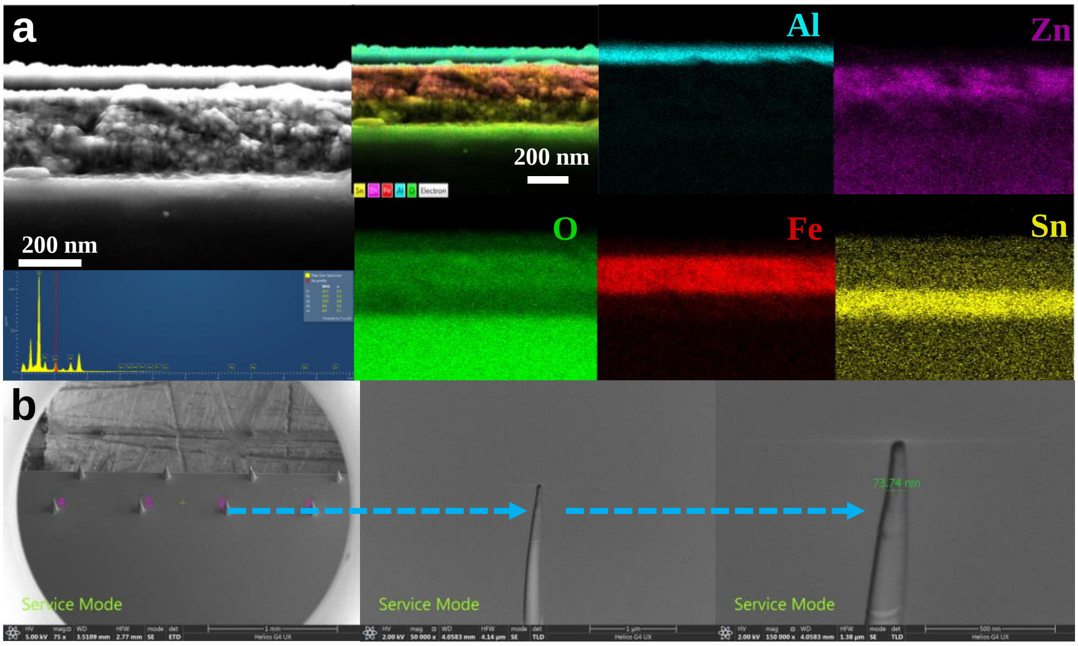

Supplementary Fig. 7. Preparation of APT tip samples. a, Side-view SEM and elemental mapping images of Al/$ZnFe_2O_4$/α-Fe_2O_3 film (Zn: Fe=1:3, The Turing interface film). **b**, SEM of the tip for the APT test.

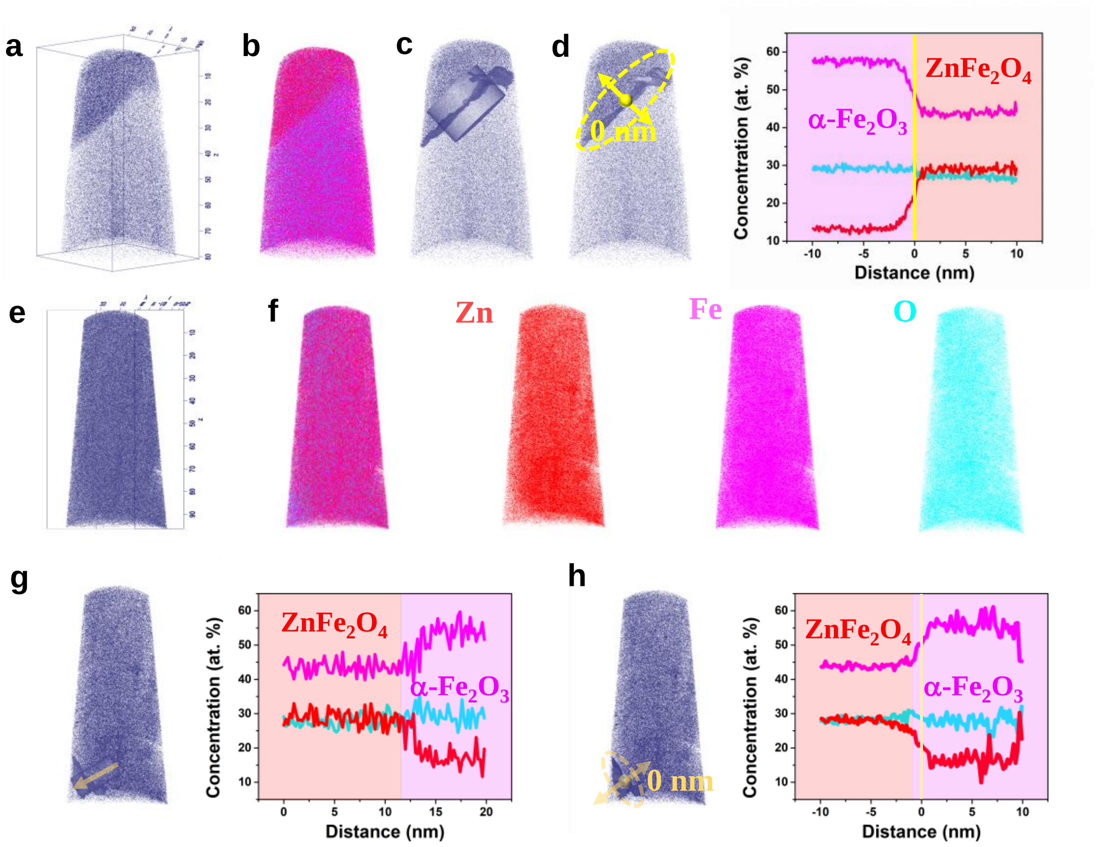

Supplementary Fig. 8. APT test of the Turing interface film (Zn: Fe=1:3). a, APT test of center of the Turing interface film (Zn: Fe=1:3). **b**, Combined and individual atom maps. **c**, One-dimensional concentration profile taken along the cylinder (as shown on the left). **d**, Proxigram, based on the circled 23 at% Zn iso-concentration surface between $ZnFe_2O_4$ and α-Fe_2O_3. **e**, APT test of edge of the Turing interface film (Zn: Fe=1:3). **f**, Combined and individual atom maps. **g**, One-dimensional concentration profile taken along the cylinder (as shown on the left). **h**, Proxigram, based on the circled 23 at% Zn iso-concentration surface between $ZnFe_2O_4$ and α-Fe_2O_3.

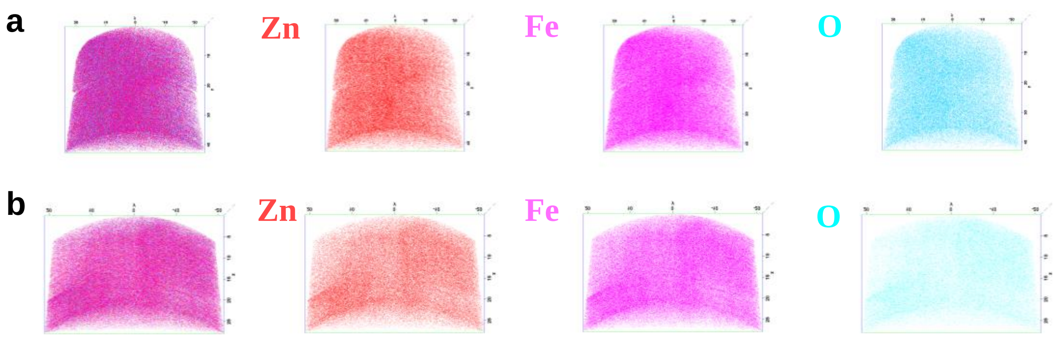

Supplementary Fig. 9. APT test of the $ZnFe_2O_4$ film. a, APT test of (20× 20 × 40 nm^3) of center of $ZnFe_2O_4$ film. **b**, APT test of (20× 20 × 25 nm^3) of edge of $ZnFe_2O_4$ film.

The local chemical composition and phase interface were evaluated in more detail with aid of the APT (Supplementary Fig. 7 and 8), which provided interface information on the phase distribution in three dimensions. Atomic-scale elemental distributions of center (20× 20 × 80 nm^3) and edge (20× 20 × 90 nm^3) of the Turing interface film (Zn: Fe=1: 3) are shown (Supplementary Fig. 10a and e), respectively. Consistent with EDX and SAED data, the corresponding one-dimensional concentration profile across the separating phases reveals the formation of $ZnFe_2O_4$ phase and α-Fe_2O_3 phase (Supplementary Fig. 8c and g). Using the Zn iso-concentration surface as the interface (Supplementary Fig. 8d), the distribution of Zn is detected in the α-Fe_2O_3 phase, indicating the existence of more new interfaces between $ZnFe_2O_4$ phase and α-Fe_2O_3 phase. Especially, it can be clearly observed that the concentration of Zn element returns to the original concentration, which provides sufficient evidence for multiple interfaces. Unlike some other methods that are prone to parasitic phases, there is no parasitic phase in the $ZnFe_2O_4$ film (Supplementary Fig. 9), which indicates that the Turing structure of the film comes from concentration modulation.

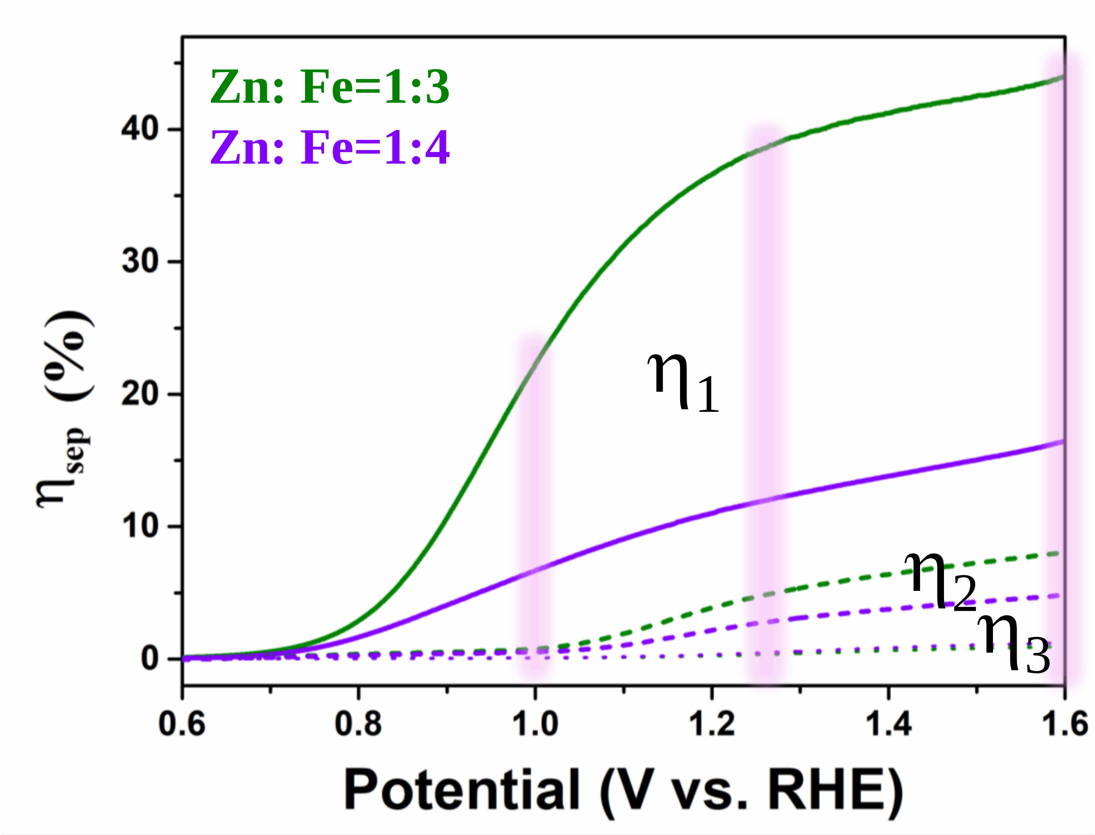

Supplementary Fig. 10. Charge separation efficiency of the photoelectrodes. η_1: The Turing interface film; η_2: non-Turing dual-phase interface film; η_3: the conventional dual-phase interface film.

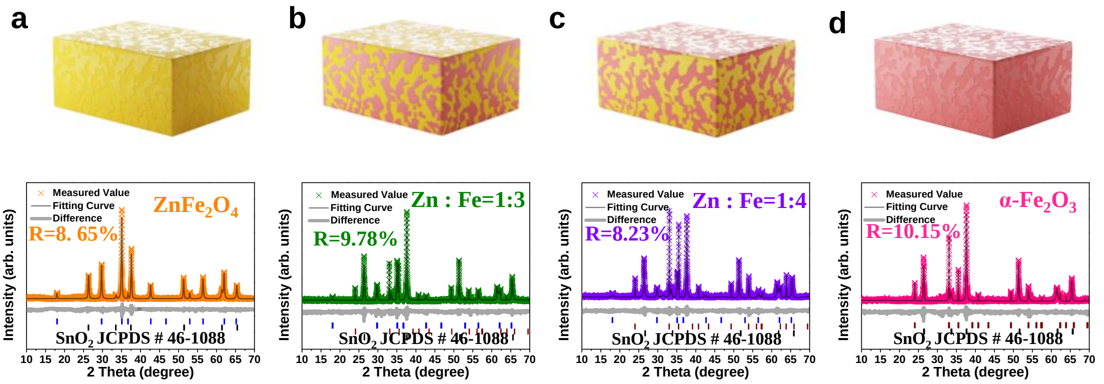

Supplementary Fig. 11. Schematic diagram of spatial structure and Rietveld refinement of XRD patterns. a, $ZnFe_2O_4$ film. **b**, Turing interface film (Zn: Fe=1:3). **c**, Turing interface film (Zn: Fe=1:4). **d**, α-Fe_2O_3 film.

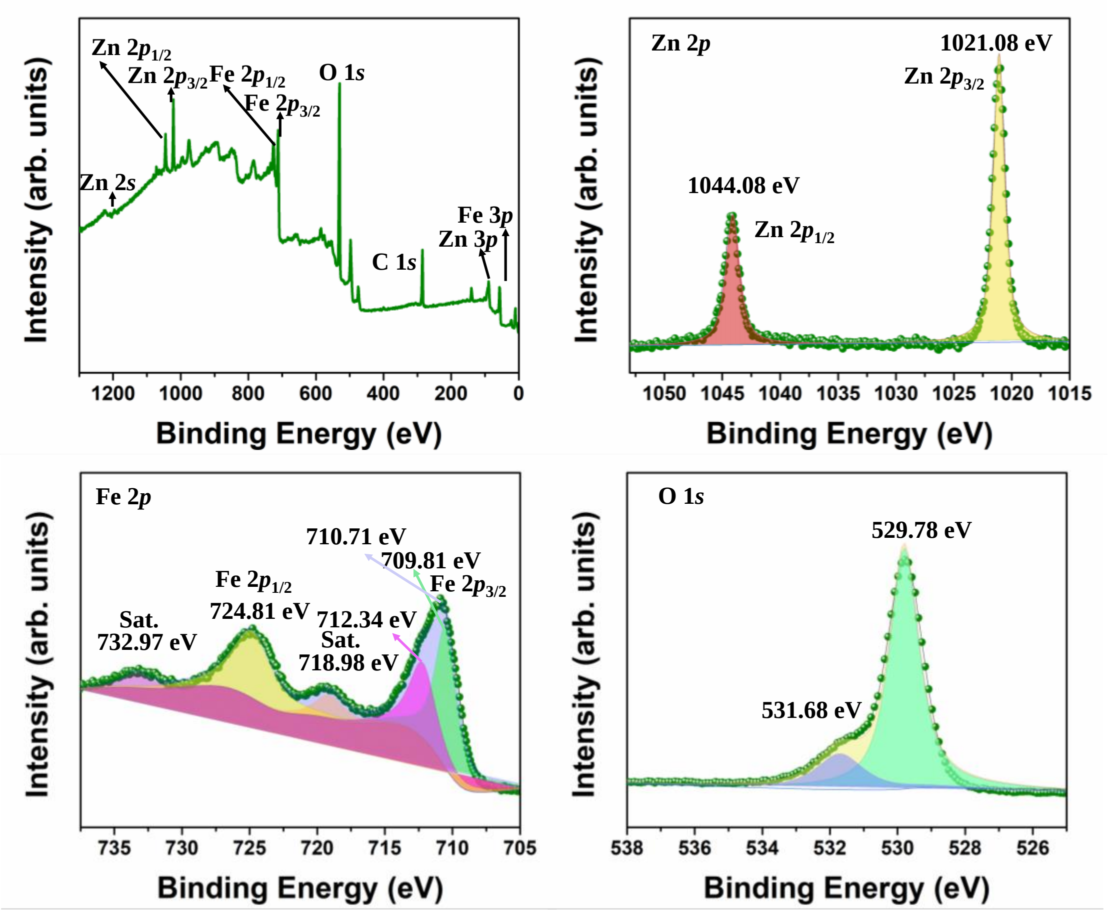

Supplementary Fig. 12. XPS data. High-resolution XPS signals of Zn 2*p*, Fe 2*p*, O 1*s* of the Turing interface film (Zn: Fe=1:3).

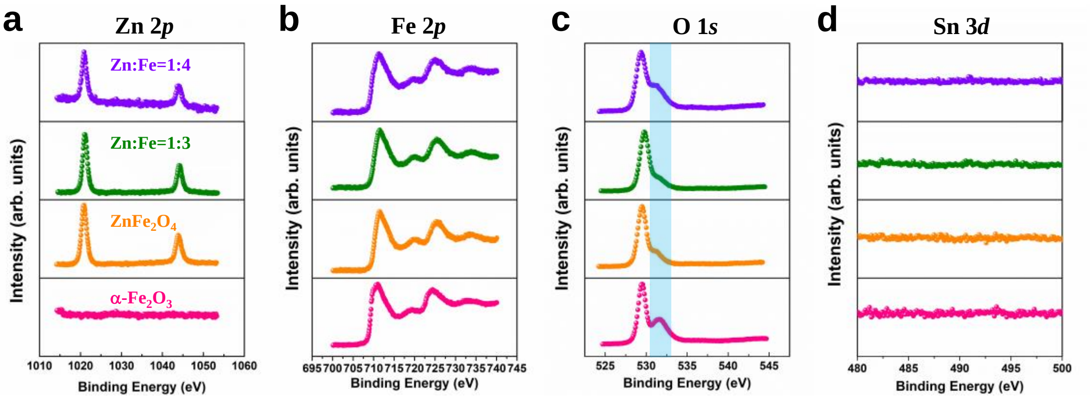

Supplementary Fig. 13. High-resolution XPS signals of Zn 2*p*, Fe 2*p*, O 1*s* and Sn 3*d*. **a**, Overlay of Zn 2*p*. **b**, Fe 2*p*. **c**, O 1*s*. **d**, Sn 3*d* XPS spectra.

X-ray photoelectron spectroscopy (XPS) is further used to study the surface chemical composition of the samples (Supplementary Fig. 12 and 13). The peaks centered at 1044.08 and 1021.08 eV are attributed to the Zn $2p_{1/2}$ and Zn $2p_{3/2}$ of Zn^{2+}, respectively. There are two main peaks of Fe $2p_{1/2}$ (724.81 eV) and Fe $2p_{3/2}$ (709.81 eV) accompanied by two satellite peaks at the binding energies of 718.98 and 732.97 eV, which are the typical values of Fe^{3+} in $ZnFe_2O_4$ and α-Fe_2O_3. Noteworthily, the absence of satellite peak at around 715 eV in the Fe 2*p* region suggest that there is no Fe^{2+}. The peaks at 529.78 and 531 eV correspond to the lattice oxygen and O-H bonds, respectively. The O-H bonds decreases significantly in comparison with that of the pure sample, suggesting possible decrease of surface states, which is beneficial for improving PEC performance. Through the fitting XPS comparison, the composition is also consistent with the proportion of the original addition. Furthermore, the 3*d* signal of Sn (Supplementary Fig. 13d) was not detected, eliminating the interference of Sn doped.

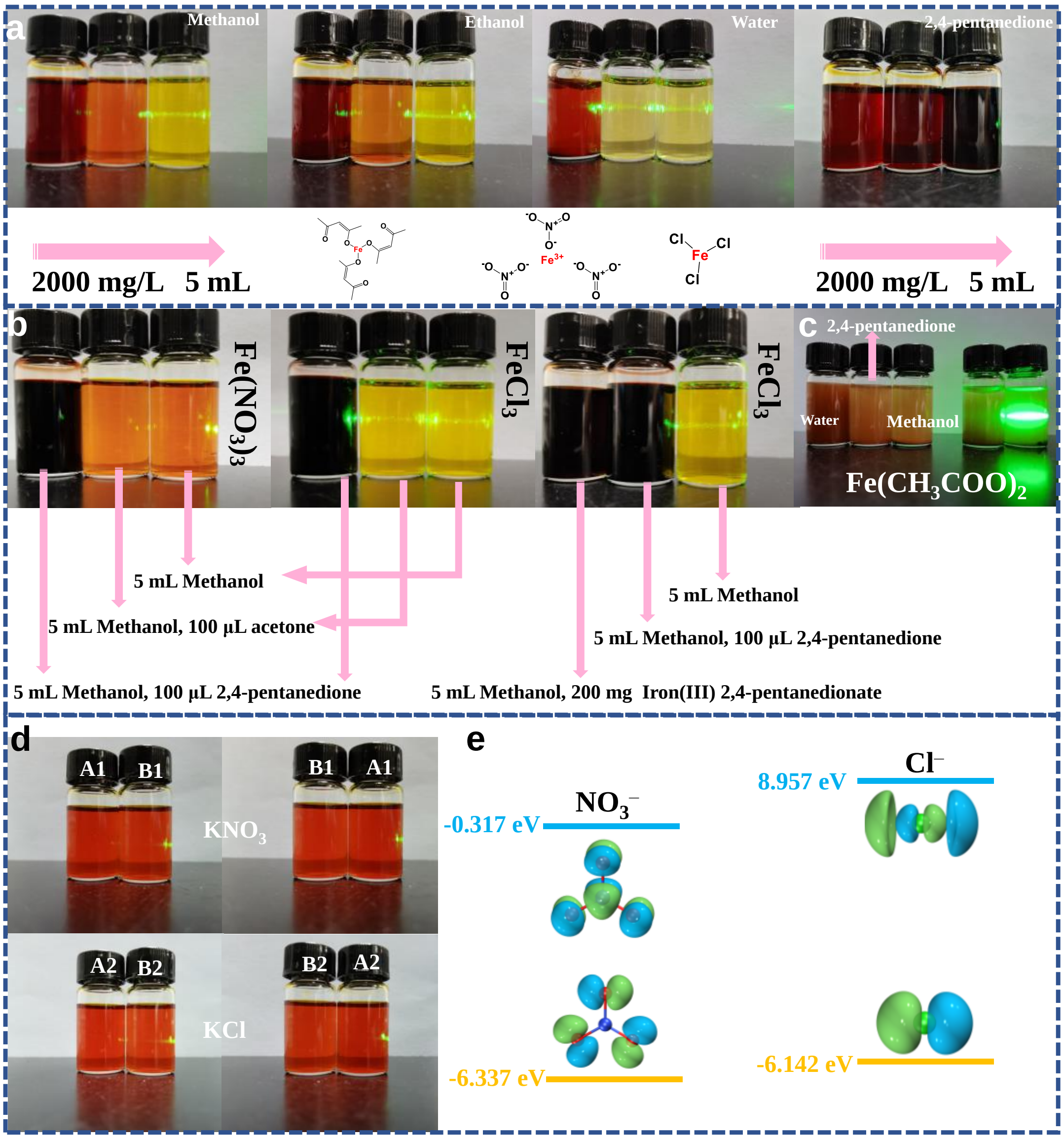

Supplementary Fig. 14. Understanding of re-coordination mechanism based on Tyndall effect. a, Tyndall effect of three raw materials (Iron (III) 2,4-pentanedionate, ferric nitrate and ferric chloride) in different solvents (Methanol, ethanol, water and 2,4-pentanedionate). **b**, Effect of different iron sources in methanol by adding acetone and 2,4-pentanedionate. **c**, Ferrous acetate shows obvious colloidal properties in different solvents. **d**, Adding the same concentration of chloride ions and nitrate ions to a solution of a certain concentration of iron (III) 2,4-pentanedionate can observe that the solution exhibits an obvious Tyndall effect, and the chloride ions are more obvious. **e**, Frontier molecular orbitals for NO_3^- and Cl^- obtained by the B3LYP/6-311G (d, p) method.

Raw materials weighed according to the same concentration of iron are dissolved

in different solutions (Methanol, ethanol, water and 2,4-pentanedionate). It can be clearly seen that ferric nitrate and ferric chloride do not exhibit colloidal properties in 2,4-pentanedionate, but exhibit obvious colloidal properties in the remaining solutions, which is mainly due to the alcoholysis of ferric ion. In iron (III) 2,4-pentanedionate ($Fe[C_5H_7O_2]_3$), iron is in a complex state and therefore does not exhibit colloidal properties (Supplementary Fig. 14a). The comparison reveals the main effect of 2,4-pentanedionate, not acetone, and also verifies its strong complexing ability in a pure 2, 4-pentanedionate solution (Supplementary Fig. 14b). The colloidal properties of ferrous acetate in different solvents was reflected (Supplementary Fig. 14c). The chemical hardness of chloride ion is greater than that of nitrate ion from experiments and calculations (Supplementary Fig. 14d and e).

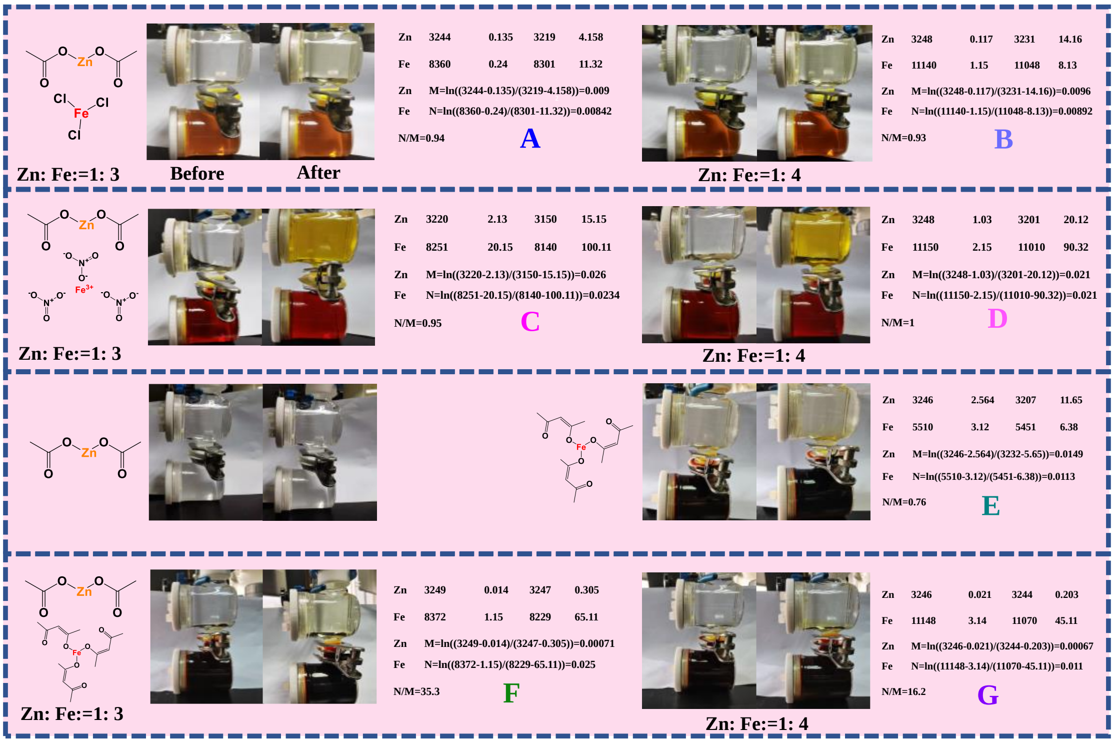

Supplementary Fig. 15. Ratio of diffusion coefficient. M, N= $\ln(c_1-c_2/c_3-c_4)$, c_1 and c_3 are the initial and final concentrations; c_2 and c_4 are the initial and final concentrations in the upper compartment).

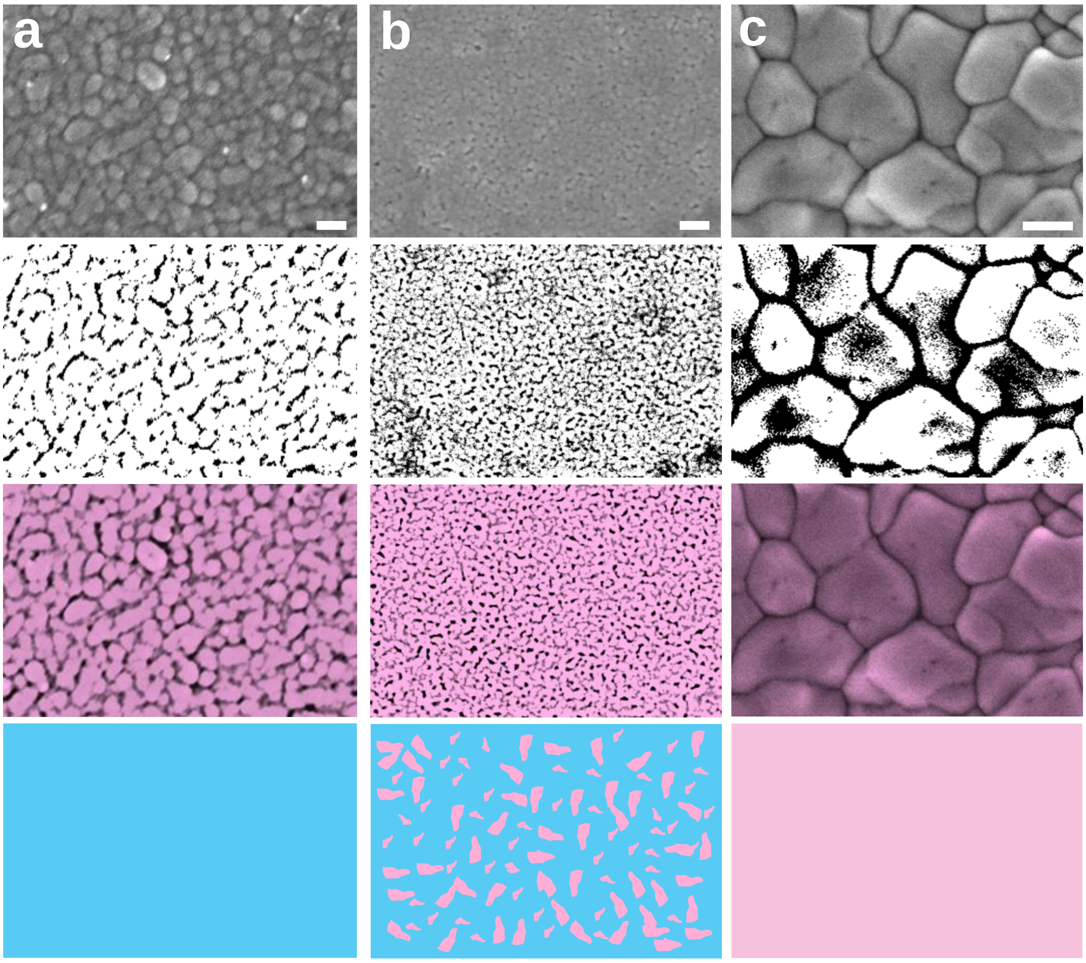

Supplementary Fig. 16. SEM images of Turing structure formation process. a, $ZnFe_2O_4$ film. **b**, Turing interface film (Zn: Fe=1:3). **c**, α-Fe_2O_3 film. In the fourth row of the figure, blue represents $ZnFe_2O_4$ and pink represents α-Fe_2O_3. Scale bars: 100 nm.

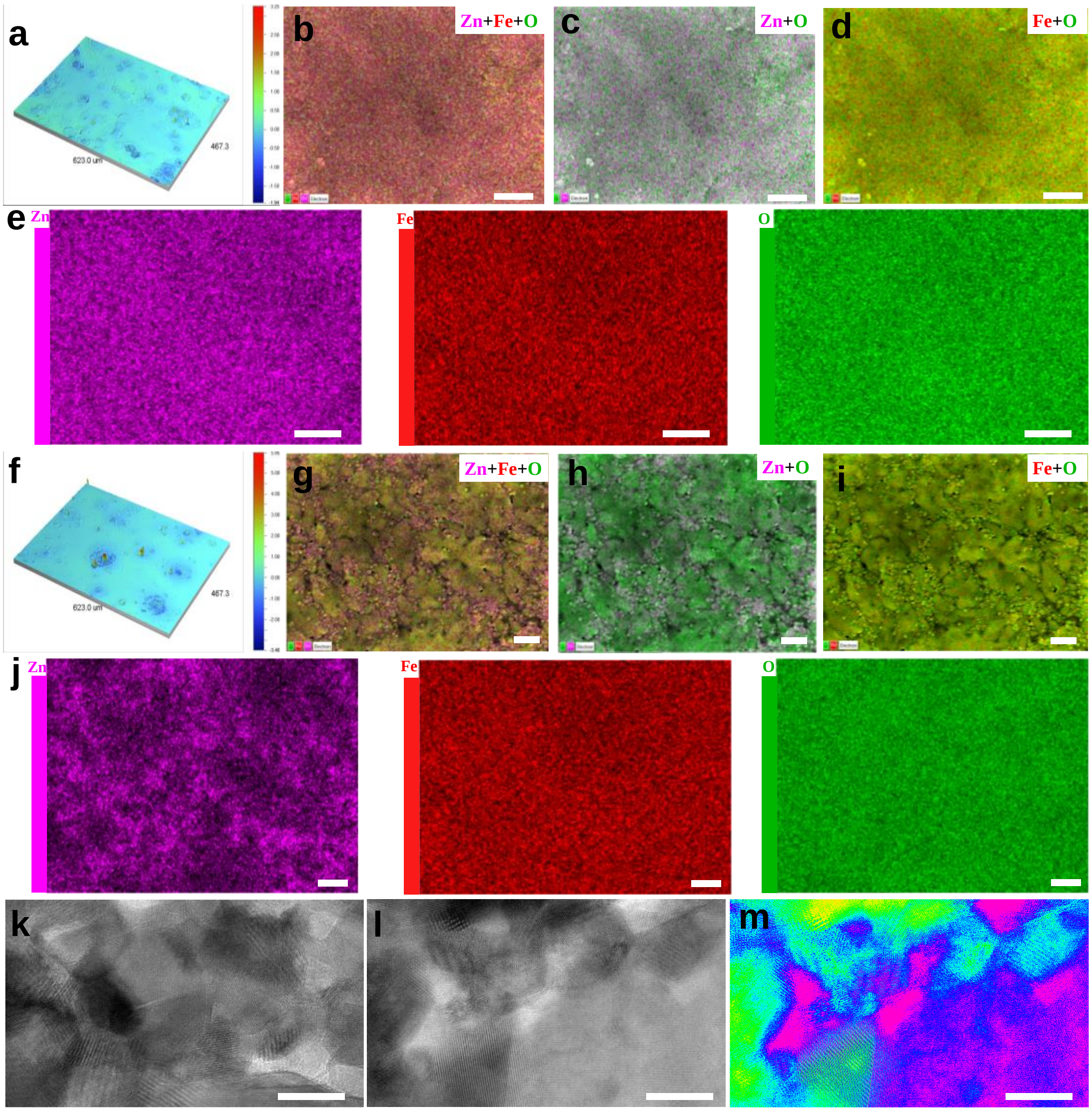

Supplementary Fig. 17. SEM images, elemental mapping and HAADF-STEM images of Turing interface film. a, Surface roughness of the Turing interface film (Zn: Fe=1:3) (Ra= 8.3 nm) measured by step profiler. **b**, **c**, **d**, **e**, Elemental mapping for the Turing interface film (Zn: Fe=1:3). **f**, Surface roughness of the Turing interface film (Zn: Fe=1:4) (Ra= 8.7 nm) measured by step profiler. **g**, **h**, **i**, **j**, Elemental mapping for the Turing interface film (Zn: Fe=1:4). **k**, HAADF-STEM image of the Turing interface film (Zn: Fe=1:3). **l**, HAADF-STEM image of the Turing interface film (Zn: Fe=1:4). **m**, False-colored HAADF-STEM image. Purple represents α-Fe_2O_3 and green represents $ZnFe_2O_4$ by the crystal plane analysis in **l**. Scale bars: **b**, **c**, **d**, **e**, **g**, **h**, **i**, **j**, 250 nm; **k**, **l**, **m**, 20 nm.

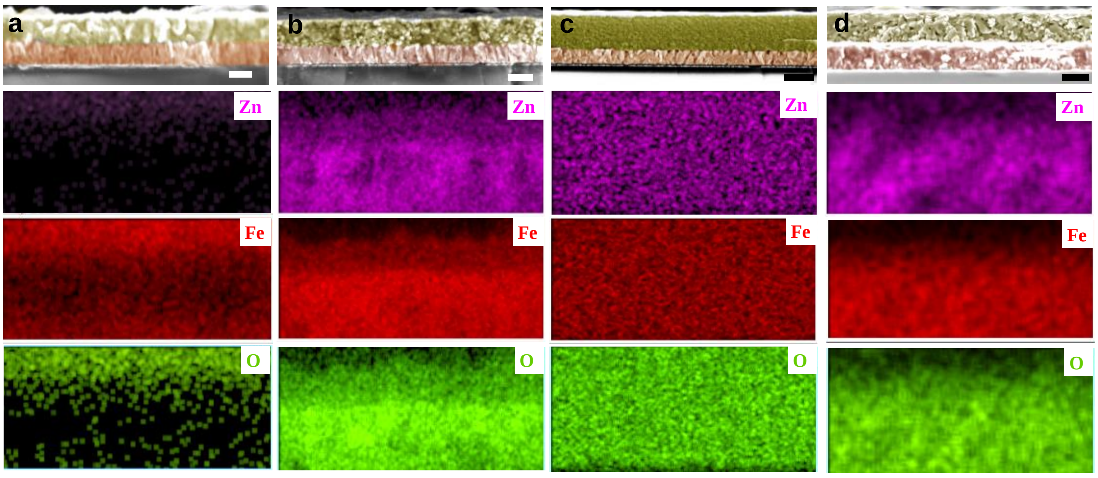

Supplementary Fig. 18. Side-view SEM images and elemental mapping. a, α-Fe_2O_3 film. **b**, $ZnFe_2O_4$ film. **c**, Turing interface film (Zn: Fe=1:3). **d**, Turing interface film (Zn: Fe=1:4). Scale bars: **a**, **b**, **c**, **d**, 300 nm.

As the concentration changes, more obvious phase separation appears, and the Turing interface is clearer (Supplementary Fig. 16), which shows the detailed SEM images, TEM images, elemental mappings (Supplementary Fig. 17) and cross-sections of different samples (Supplementary Fig. 18), and the thickness of the films is about 300 nm.

Supplementary Note 1. Numerical Simulation

Reaction-diffusion model for pattern formation have received increasing attention from theoretical biology and applied mathematics. The equations are of the following form:

$$\begin{cases} \frac{\partial u}{\partial t} = f(u,v) + d_u \Delta u \\ \frac{\partial v}{\partial t} = g(u,v) + d_v \Delta v \end{cases} \tag{1}$$

where u and v is the vector of concentration, the functions $f(u,\ v)$ and $g(u,\ v)$ represent the reaction kinetics (diffusion term), d_u and d_v are the diffusion coefficients of u and v, respectively. Researchers have found examples of Turing-like mechanisms in the distribution of species in ecosystems, such as the predator-prey model. In our experiment, substance A (Fe) and substance B (Zn) with large difference in diffusion coefficient correspond to inhibitor (predator) and activator (prey), respectively. This type of model takes the form[23]:

$$\begin{cases} \frac{du}{dt} = ru\left(1 - \frac{u}{K}\right) - \frac{muv}{u+a} \\ \frac{dv}{dt} = \frac{buv}{u+a} - cv \end{cases} \tag{2}$$

Applying the scaling to system: $rt \to t,\ \frac{u}{K} \to u,\ \frac{m}{rK} v \to v,\ \frac{a}{K} \to \alpha,\ \frac{b}{r} \to \beta,\ \frac{c}{r} \to \gamma$

Then

$$\begin{cases} \frac{du}{dt} = u(1-u) - \frac{uv}{u+\alpha} \\ \frac{dv}{dt} = \frac{\beta uv}{u+\alpha} - \gamma v \end{cases} \tag{3}$$

$$\begin{cases} u_t = d_A \Delta u + u(1-u) - \frac{uv}{u+\alpha} & x \in \Omega, t > 0 \\ v_t = d_B \Delta v + \frac{\beta uv}{u+\alpha} - \gamma v & x \in \Omega, t > 0 \\ \frac{\partial u}{\partial n} = \frac{\partial v}{\partial n} = 0 & x \in \partial\Omega, t > 0 \\ u(x,0) = u_0 \geq 0 \quad v(x,0) = v_0 \geq 0 & x \in \Omega \end{cases} \tag{4}$$

where u and v represent the diffusion functions of B and A in the experimental system respectively, d_A and d_B are the diffusion coefficients of A and B, respectively. Ω is a fixed bounded domain (FTO substrate boundary), n is the outward unit normal vector of the boundary, Δ is Laplace operator, $\frac{\partial u}{\partial n} = \frac{\partial v}{\partial n} = 0$ is the homogeneous boundary condition, $u(x,0) = u_0 \geq 0$ and $v(x,0) = v_0 \geq 0$ are continuous functions.

The positive equilibrium point $E^*(u^*, v^*)$ can be obtained by judging the instability of equation 4. $u^* = \frac{\alpha\gamma}{\beta-\gamma}$ $\quad v^* = (\alpha + u^*)(1 - u^*)$ $\quad \beta > \gamma$ $\quad \alpha < \frac{\beta-\gamma}{\gamma}$

A time perturbation (t) is applied around E^*.

$$\begin{cases} \hat{u}(r,t) = u_0 e^{\lambda t} e^{i\overrightarrow{kr}} & \hat{v}(r,t) = v_0 e^{\lambda t} e^{i\overrightarrow{kr}} \\ |\hat{u}(r,t)| \ll u^* & |\hat{v}(r,t)| \ll v^* \end{cases} \tag{5}$$

where $\vec{r}$ is space vector.

$$\begin{cases} g(u) = 1 - u \quad p(u) = \frac{u}{\alpha+u} \\ h(u) = \frac{ug(u)}{p(u)} = (1-u)(\alpha+u) \\ f_u = p(u^*)h'(u^*) \quad f_v = -p(u^*) \\ g_u = \frac{\gamma^2}{\beta} \quad g_v = -\gamma \quad d = \frac{d_B}{d_A} \quad D = \begin{pmatrix} 1 & 0 \\ 0 & d \end{pmatrix} \end{cases} \tag{6}$$

The Jacobian matrix at the positive equilibrium is shown below:

$$J(E^*) = \begin{pmatrix} p(u^*)h'(u^*) & -p(u^*) \\ \frac{\gamma^2}{\beta} & -\gamma \end{pmatrix} \tag{7}$$

We can derive from the linear system:

$$|\lambda I - J + Dk^2| = 0 \tag{8}$$

The characteristic function of equation 5 is as follows:

$$\begin{cases} \lambda^2 + \lambda[k^2(1+d) - (f_u + g_v)] + h(k^2) = 0 \\ h(k^2) = dk^4 - (df_u + g_v)k^2 + |J| \\ |J| = \gamma p(u^*)\left[\frac{\gamma}{\beta} - h'(E^*)\right] \end{cases} \tag{9}$$

The condition for a spatial mode defined by k to be unstable and thus to form a pattern in equation 9. $Re(\lambda)$ is a function of $h(k^2)$, and $h(k^2)$ is closely related with d. Therefore, $d(d = \frac{d_B}{d_A})$ determines the range of $Re(\lambda)$ and the stability of the diffusion system. Next, we use numerical simulation[24] to verify whether the experimental system is unstable. First, we simplify equation 4 to the following form:

$$\begin{cases} f(u,v) = u(1-u) - \frac{uv}{u+\alpha} \\ g(u,v) = \frac{\beta uv}{u+\alpha} - \gamma v \end{cases} \tag{10}$$

For the two-dimensional approximations, we use a uniform subdivision of the square by finite difference method.

$$\begin{cases} \Omega = [A,B] \times [A,B] \quad (x_i, y_j) = (ih + A, jh + A) \quad i,j = 0,\cdots,J \\ h = \frac{B-A}{J} \end{cases} \tag{11}$$

$\vec{U}_{i,j}^n = (U_{i,j}^n, V_{i,j}^n)^T$ denotes the two-dimensional approximation at the point (x_i, y_j, t_n). We also carry out a uniform subdivision of the time interval [0, T] with time levels $t_n = n\Delta t$, $n = 1, \cdots, N$, so the time step is $\Delta t = \frac{T}{N}$. h is space step. Two-dimensional linear schemes are revealed as the following general form. For $n = 1, \cdots, N$ and $i,j = 0, \cdots, J$ find $\{U_{i,j}^n, V_{i,j}^n\}$

$$\begin{cases} \partial_n U_{i,j}^n = \Delta h U_{i,j}^n + \hat{f}(U_{i,j}^n, U_{i,j}^{n-1}) \\ \partial_n V_{i,j}^n = d\Delta h V_{i,j}^n + \hat{g}(U_{i,j}^n, U_{i,j}^{n-1}) \end{cases} \tag{12}$$

$U_{i,j}^0 \coloneqq u_0(x_i, y_j)$ and $V_{i,j}^0 \coloneqq v_0(x_i, y_j)$ can be understood as the initial position of B and A entering the solution.

Description in the MATLAB:

Function $[X, Y, U, V, reu, rev] = fd2d\_predator\_prey(\alpha, \beta, \gamma, d, a, b, h, T, \Delta t)$.

where X and Y are plane coordinates in simulation space (solution), U and V are spatiotemporal response of two diffusing substances, reu and rev represent the result of each iteration, α, β and γ represent the parameter in formula 10, d represent the ratio of the diffusion coefficients of two substances, a and b represent spatial positive domain (solution), h is space step, T is the maximum time for system simulation and Δt is time step.

We simulated the experiment with fixed parameters. $\alpha = 0.4, \beta = 2.0, \ \gamma = 0.6, a = 0, b = 400, h = 1, \Delta t = \frac{1}{3}, \ d = \frac{1}{30}$ or $d = \frac{1}{20}$

$$\begin{cases} U_{i,j}^0 = \frac{6}{35} - 2 * 10^{-7} * (X(i,j) - 0.1 * Y(i,j) - 225) * \\ (X(i,j) - 0.1 * Y(i,j) - 675) \\ V_{i,j}^0 = \frac{116}{245} - 3 * 10^{-5} * (X(i,j) - 450) - 1.2 * 10^{-4} * (Y(i,j) - 150) \end{cases} \tag{13}$$

$$\begin{cases} U_{i,j}^0 = \frac{6}{35} - 2 * 10^{-7} * (X(i,j) - 180)(X(i,j) - 520) - 6 * 10^{-7} * \\ (Y(i,j) - 80)(X(i,j) - 200) \\ V_{i,j}^0 = \frac{116}{245} - 3 \times 10^{-5} * (X(i,j) - 350) - 6 * 10^{-5} * (Y(i,j) - 235) \end{cases} \tag{14}$$

When $T = 1, T = 5, T = 10, \cdots, T = 1000$, the simulated Turing pattern can be obtained.

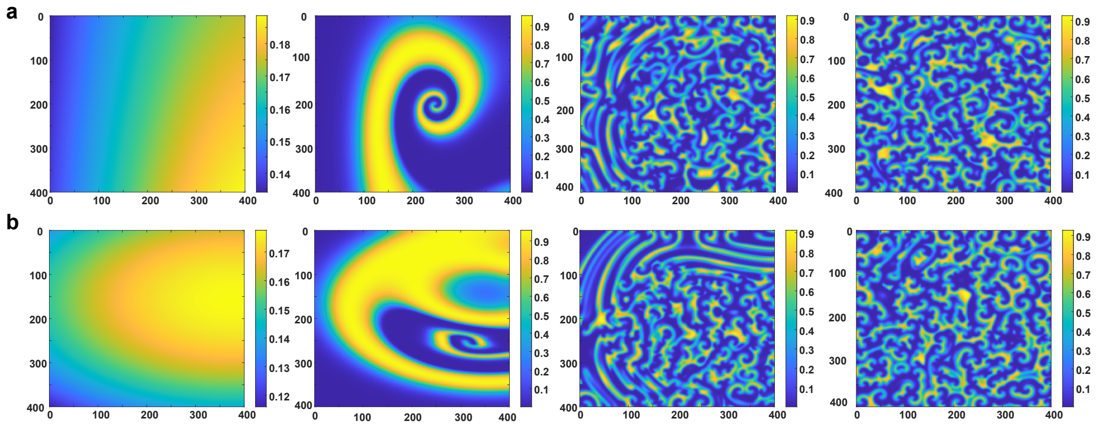

Supplementary Fig. 19. Numerical simulation. a, Patterns exhibited by prey and predator of the system at T =1, 100, 600 and 1000, respectively. $d = \frac{1}{30}$, and the initial data is in line with the equation 13. **b**, Patterns exhibited by prey and predator of the system at T =1, 100, 600 and 1000, respectively. $d = \frac{1}{20}$, and the initial data is in line with the equation 14.

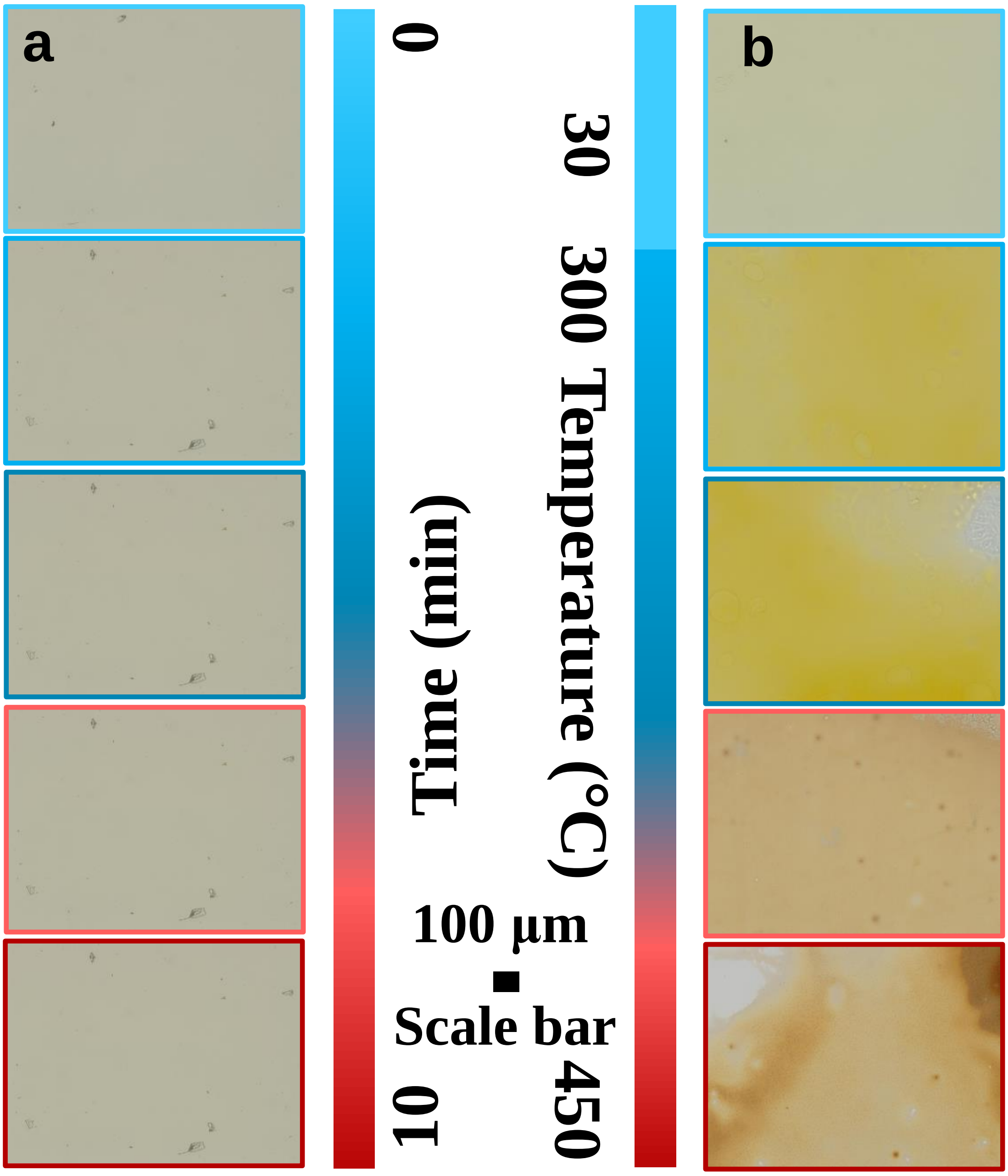

Supplementary Fig. 20. Optical observation of shape evolution of the droplets. a, The shape evolution of the droplets on the FTO for A solution (Supplementary Fig. 15) at room temperature. **b**, The shape evolution of the droplets on the FTO for A solution at different temperatures.

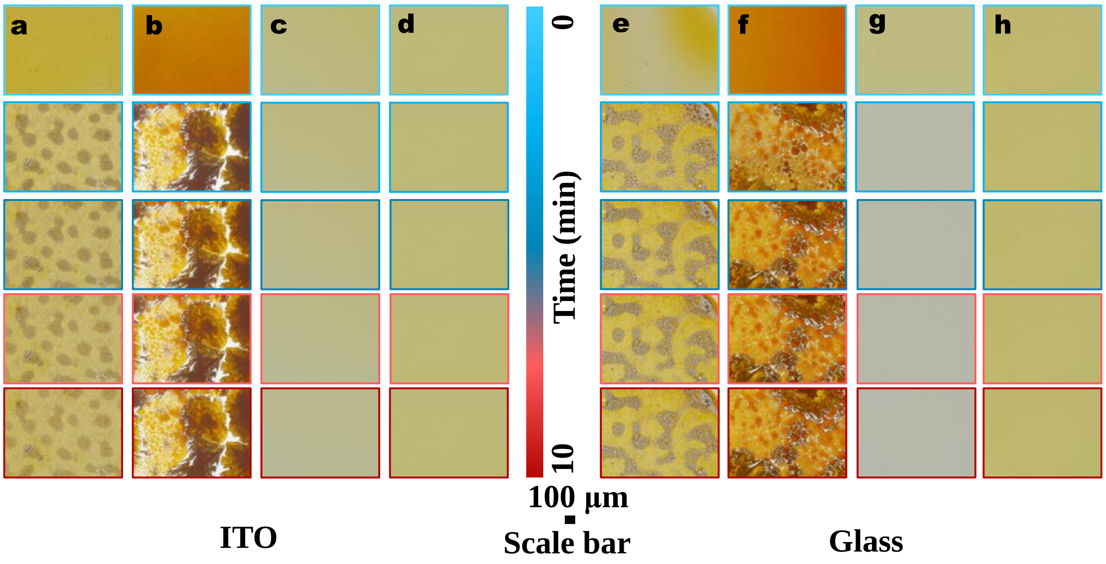

Supplementary Fig. 21. *In situ* optical observation of shape evolution of the droplets. a, **b**, **c**, **d**, The shape evolution of the droplets on the ITO for F, G, C and A solution (Supplementary Fig. 15) at room temperature, respectively. **e**, **f**, **g**, **h**, The shape evolution of the droplets on the glass for F, G, C and A solution (Supplementary Fig. 15) at room temperature, respectively.

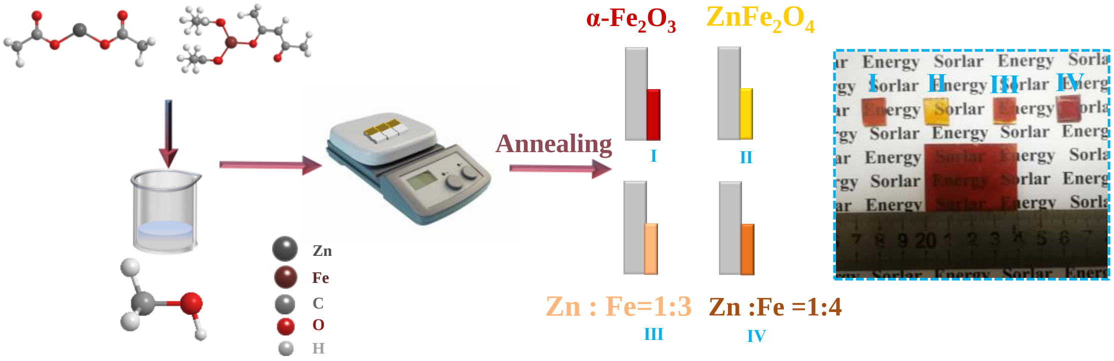

Supplementary Fig. 22. Schematic illustration for the preparation of photoelectrode films.

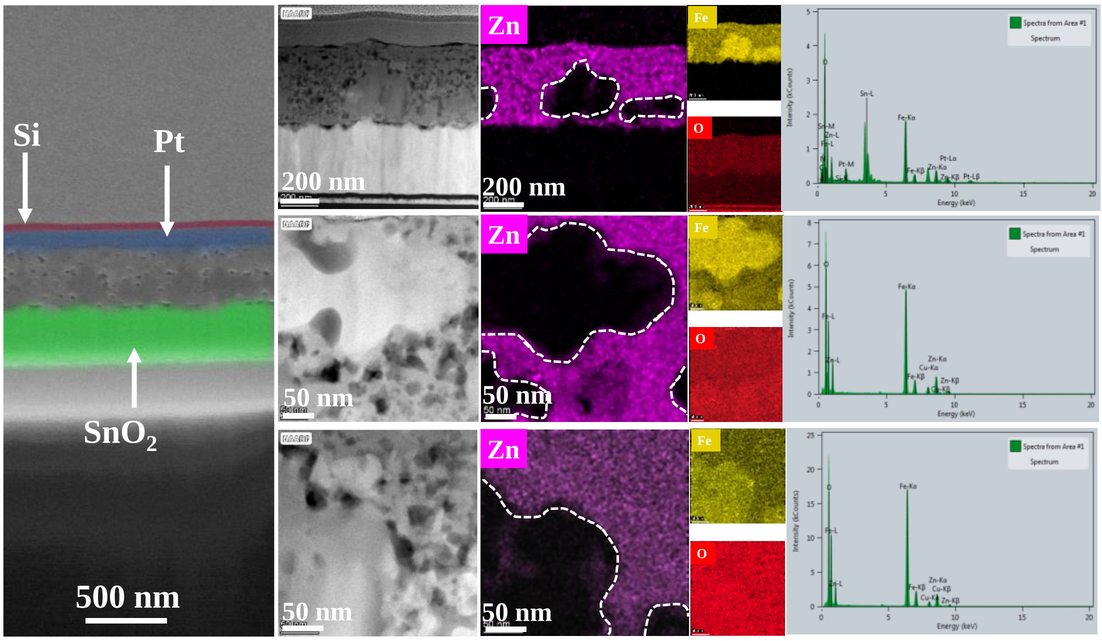

Supplementary Fig. 23. HAADF-STEM images. HAADF-STEM images and corresponding elemental mapping results in different positions for the Turing interface film (Zn: Fe=1:3).

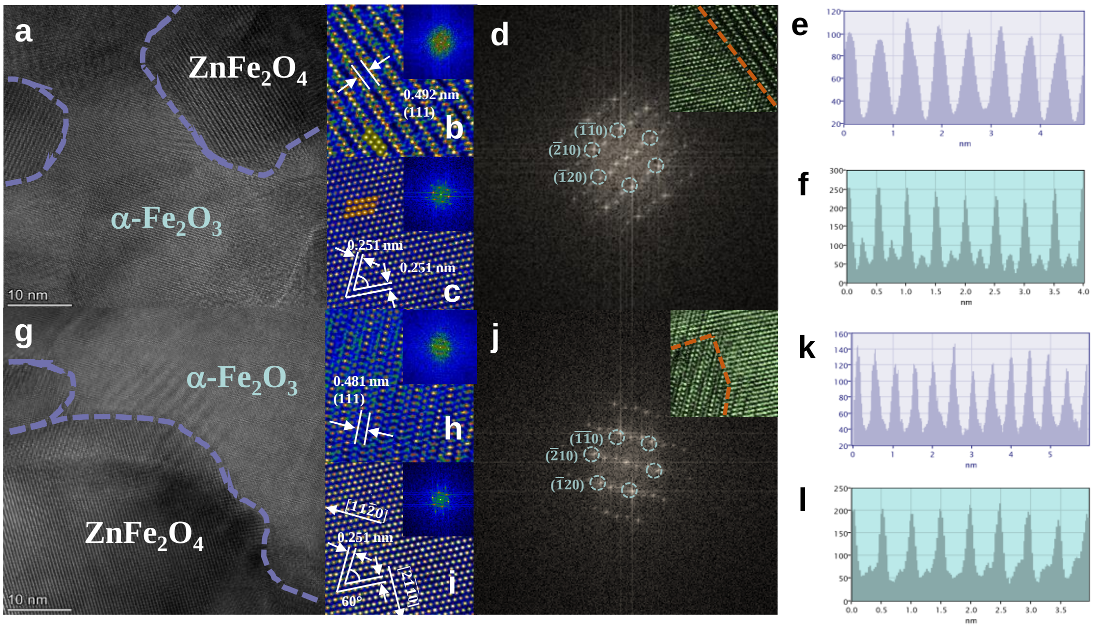

Supplementary Fig. 24. HAADF-STEM images. a, **g**, HAADF-STEM images showing the atomic ordering at the interface of $ZnFe_2O_4$ and α-Fe_2O_3. **b**, **h**, Colored inverse Fourier transform power spectrum (FFT) shows the atomic ordering of $ZnFe_2O_4$ phase. The upper right corner shows the colored FFT (the inset shows the atomic model of Zn visualized. **c**, **i**, The corresponded colored inverse Fourier transform power spectrum (FFT) shows the atomic ordering of α-Fe_2O_3 phase as visualized along the [001] direction. The upper right corner shows the colored FFT. (The atomic model of Fe visualized from the [001] direction). **d**, **j**, Colored FFT and the corresponded colored inverse Fourier transform power spectrum (FFT) shows the atomic ordering between $ZnFe_2O_4$ and α-Fe_2O_3 interface as visualized along the [001] direction. **e**, **k**, Line profile from $ZnFe_2O_4$ in **b** and **h**. **f**, **l**, Line profile from α-Fe_2O_3 in **c** and **i**.

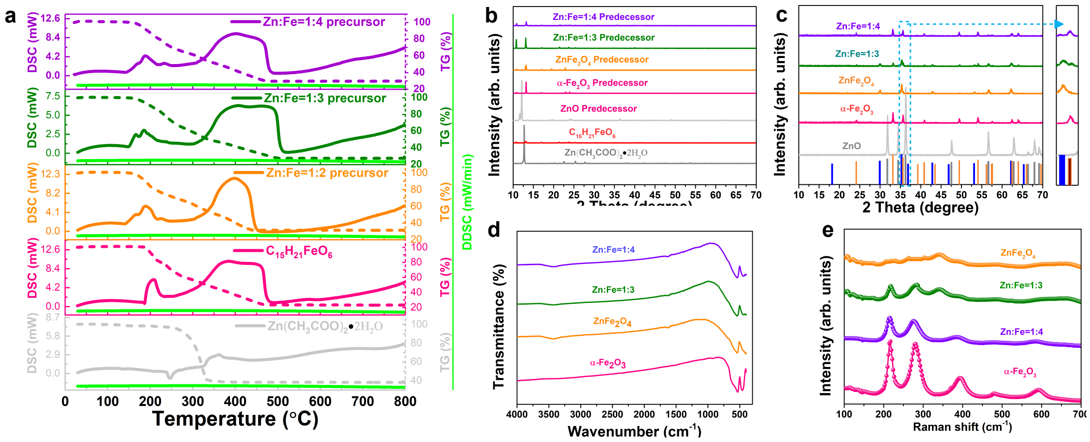

Supplementary Fig. 25. Composition analysis. a, TG-DSC curves of the precursors in different proportions. **b**, XRD patterns for the precursors. **c**, XRD patterns of powder after calcination. **d**, FT-IR spectra for powder. **e**, Raman spectra for the films.

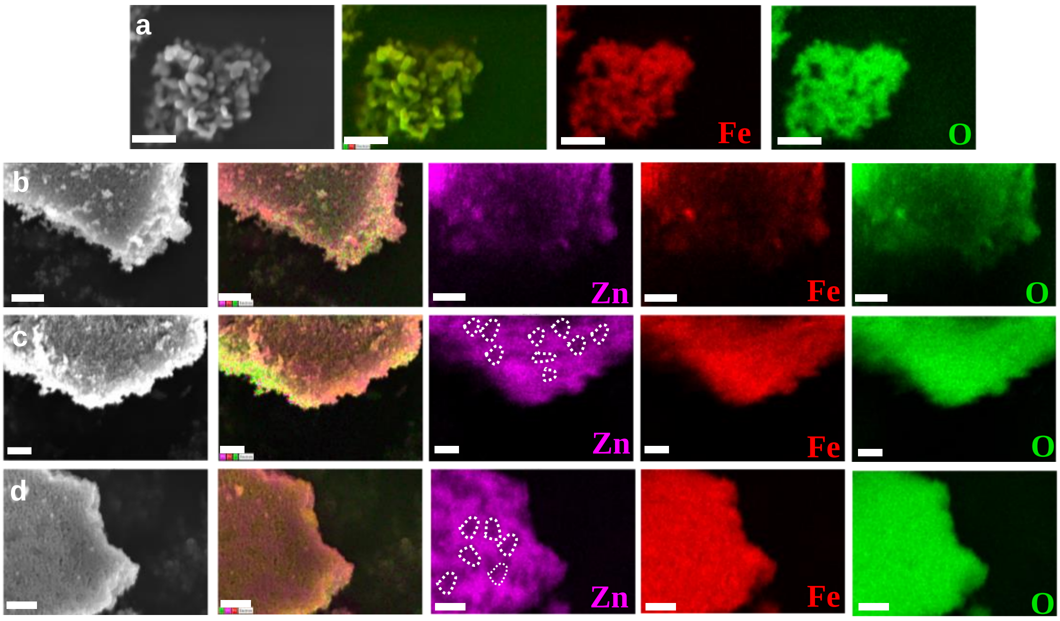

Supplementary Fig. 26. Elemental mappings. a, α-Fe_2O_3 powder. **b**, $ZnFe_2O_4$ powder. **c**, $ZnFe_2O_4$/α-Fe_2O_3 powder (Zn: Fe=1:3). **d**, $ZnFe_2O_4$/α-Fe_2O_3 powder (Zn: Fe=1:4). Scale bars: 500 nm.

The powder prepared by almost the same process as spray pyrolysis to prepare the film in order to study the film formation process. The TG-DSC, XRD, FT-IR and SEM images of different samples were shown (Supplementary Fig. 25 and 26). It can be clearly concluded from the curves that the decomposition of the raw materials corresponds to the production of oxides, and then the oxides undergo solid-phase reaction to form spinel ferrite. The excess iron exists in the form of iron oxide.

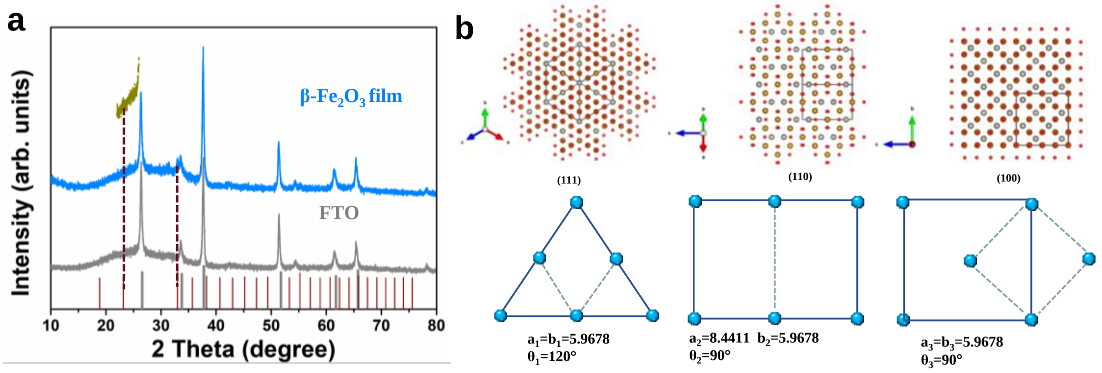

Supplementary Fig. 27. Structural analysis. a, XRD patterns for the unannealed film for the only iron source ($Fe[C_5H_7O_2]_3$). **b**, Corresponding (111), (110) and (100) crystal planes and crystal plane parameters of cubic $ZnFe_2O_4$.

The XRD patterns of β-Fe_2O_3 powder, α-Fe_2O_3 powder and the unannealed film were shown (Supplementary Fig. 27a). The reaction of β-Fe_2O_3 was as an intermediate phase, which can be concluded from the above that the unannealed film contains cubic phase β-Fe_2O_3.

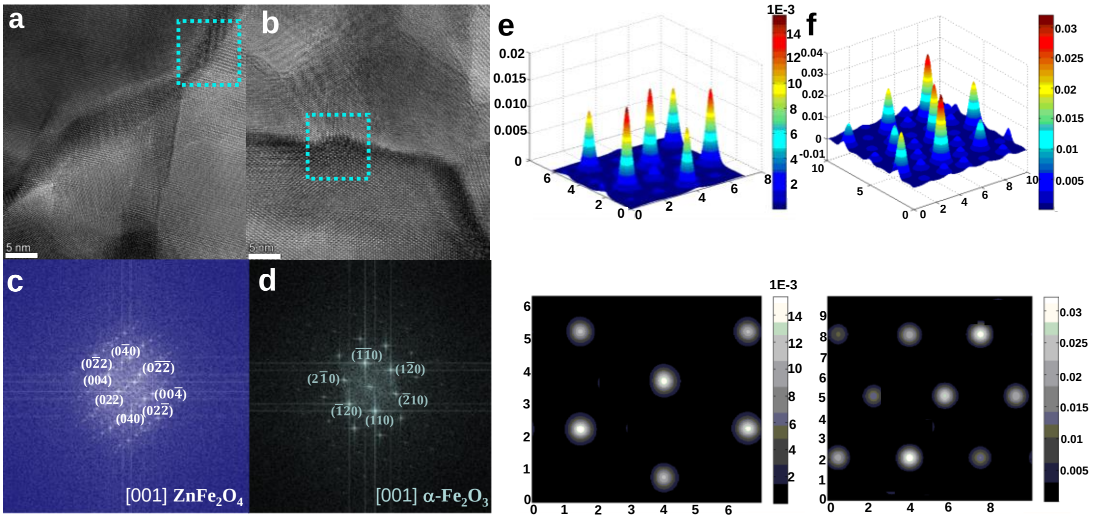

Supplementary Fig. 28. HAADF-STEM images. a, **b**, HAADF-STEM images showing the atomic ordering at the interface of $ZnFe_2O_4$ and α-Fe_2O_3. **c**, **d**, Corresponding colored FFT spectrum indicates that the crystallizes in the $ZnFe_2O_4$ and α-Fe_2O_3 phase as visualized along the [001] direction. Atom columns simulated by using QSTEM software. **e**, α-Fe_2O_3 (001). **f**, $ZnFe_2O_4$ (111). Scale bars: **a**, **b**, 5 nm.

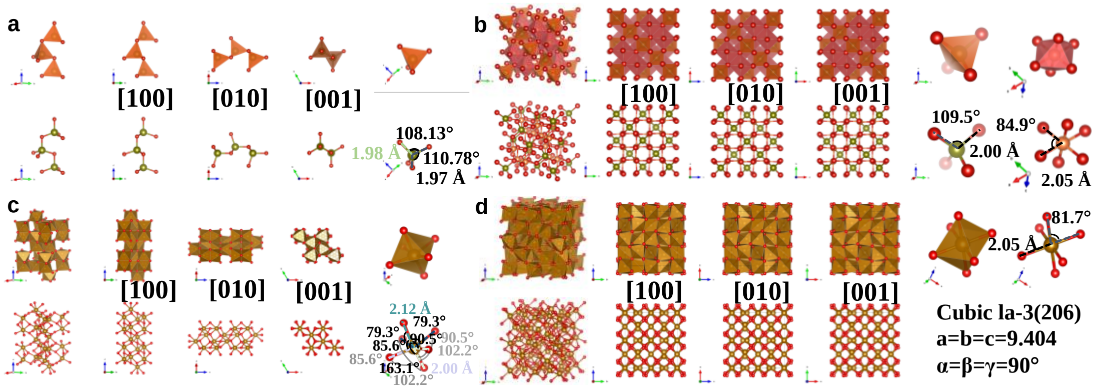

Supplementary Fig. 29. Crystal structure. a, ZnO. **b**, $ZnFe_2O_4$. **c**, α-Fe_2O_3. **d**, β-Fe_2O_3.

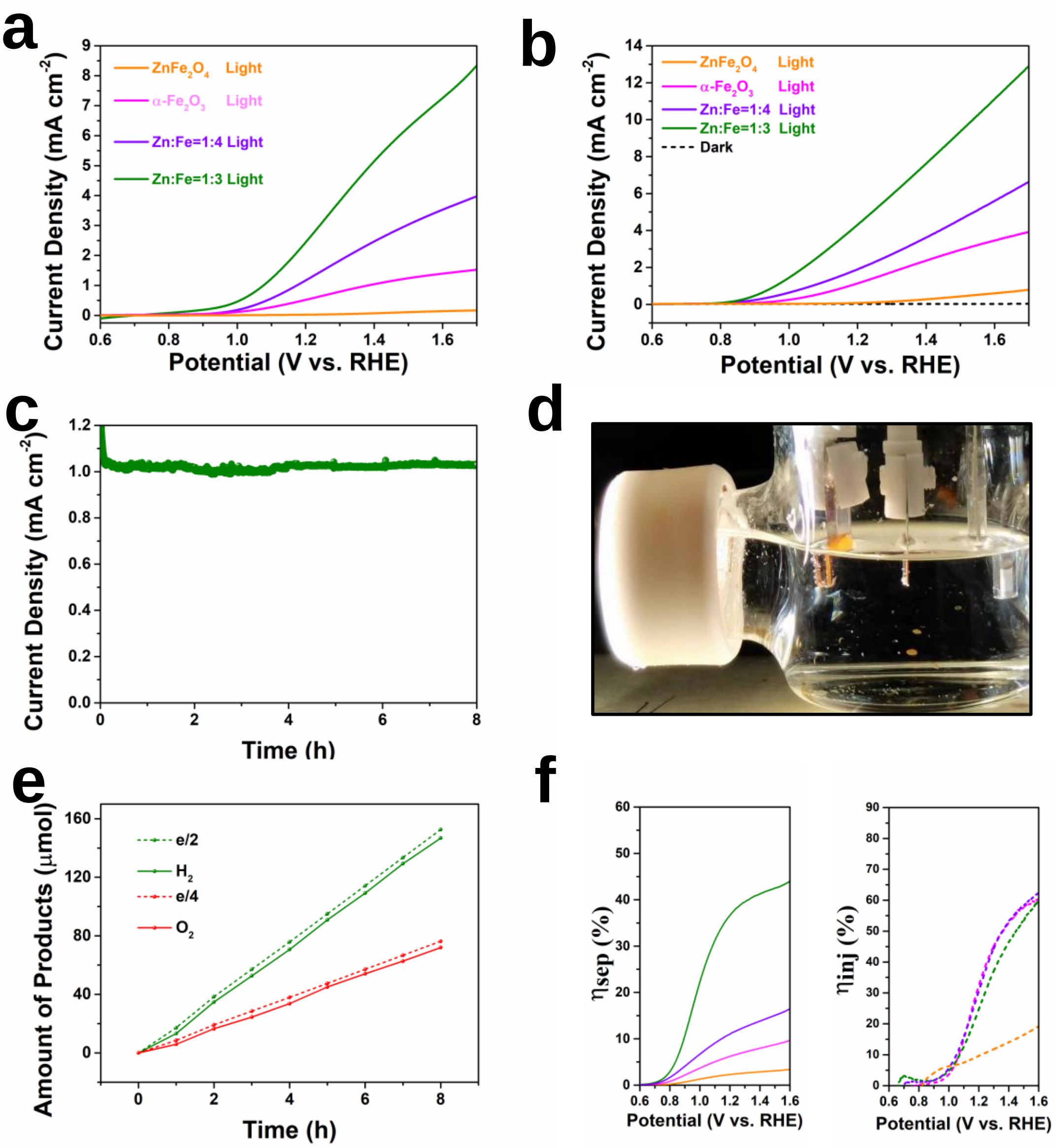

Supplementary Fig. 30. Photoelectrochemical activities for the Turing interface films. a, **b**, Current density-potential profiles of different films measured under LED illumination (296 mW cm^{-2}) and Xe lamp equipped with a convex 533 lens (500 mW cm^{-2}), respectively. **c**, Stability test of the Turing interface film (Zn: Fe=1:3) with an effective area of 1 cm^2 for 8h under AM 1.5 G illumination (100 mW cm^{-2}) at 1.23 V vs. RHE. **d**, A photograph of water-splitting device. **e**, Gas evolution for PEC water splitting of the Turing interface film (Zn: Fe=1:3) with an effective area of 1 cm^2 were performed at 1.23 V RHE under AM 1.5 G illumination in 1M NaOH electrolyte. **f**, Charge separation efficiency and charge injection efficiencies of the photoelectrodes.

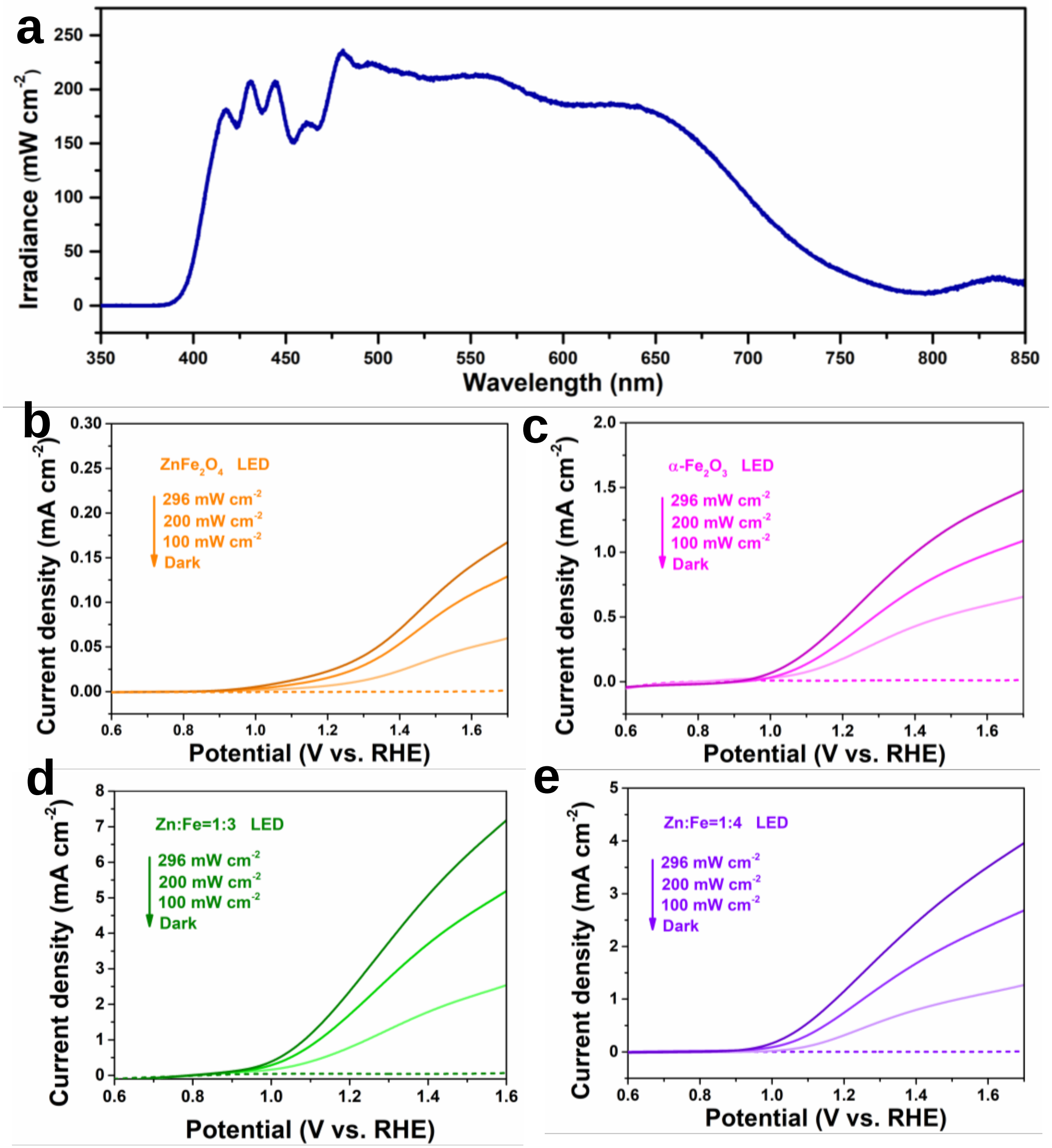

Supplementary Fig. 31. Photoelectrochemical activities under LED illumination. **a,** Spectral irradiance of a LED light. **b**, **c**, **d**, **e**, Current density-potential profiles of different films measured under LED illumination.

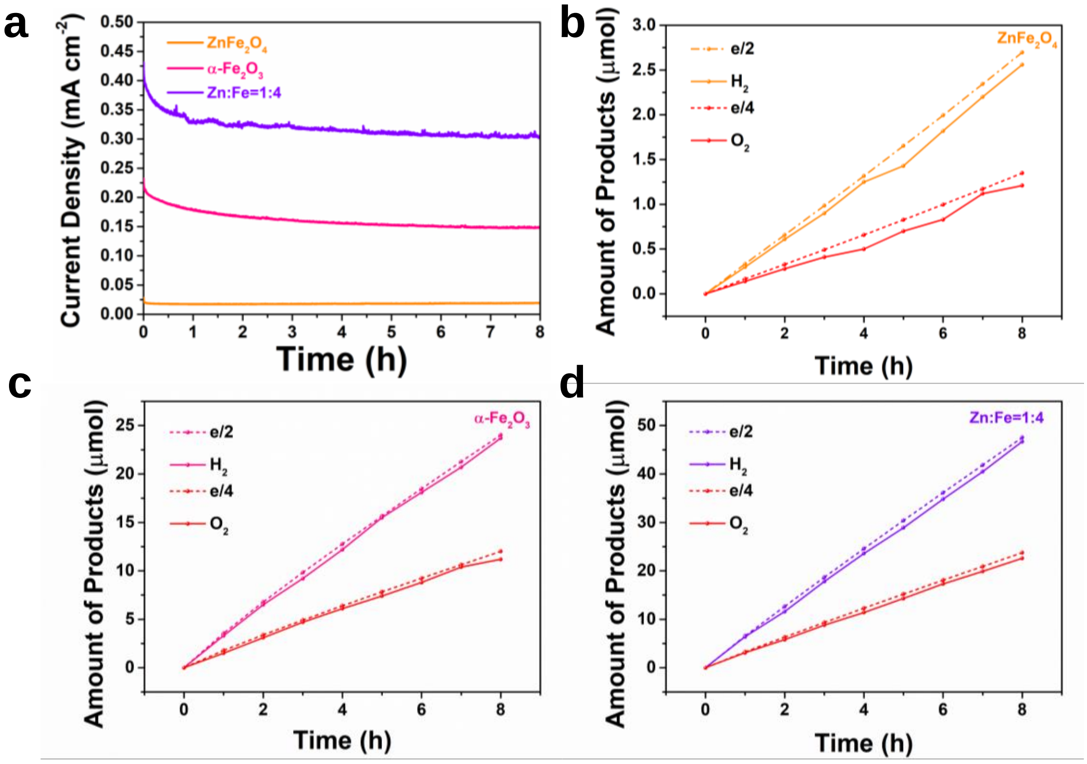

Supplementary Fig. 32. Faraday efficiency. a, Stability test of α-Fe_2O_3, $ZnFe_2O_4$ and Zn: Fe=1:4 film with an effective area of 1 cm^2 for 8h. **b**, **c**, **d**, Gas evolution curves under AM 1.5 G illumination (100 mW cm^{-2}) at 1.23 V vs. RHE.

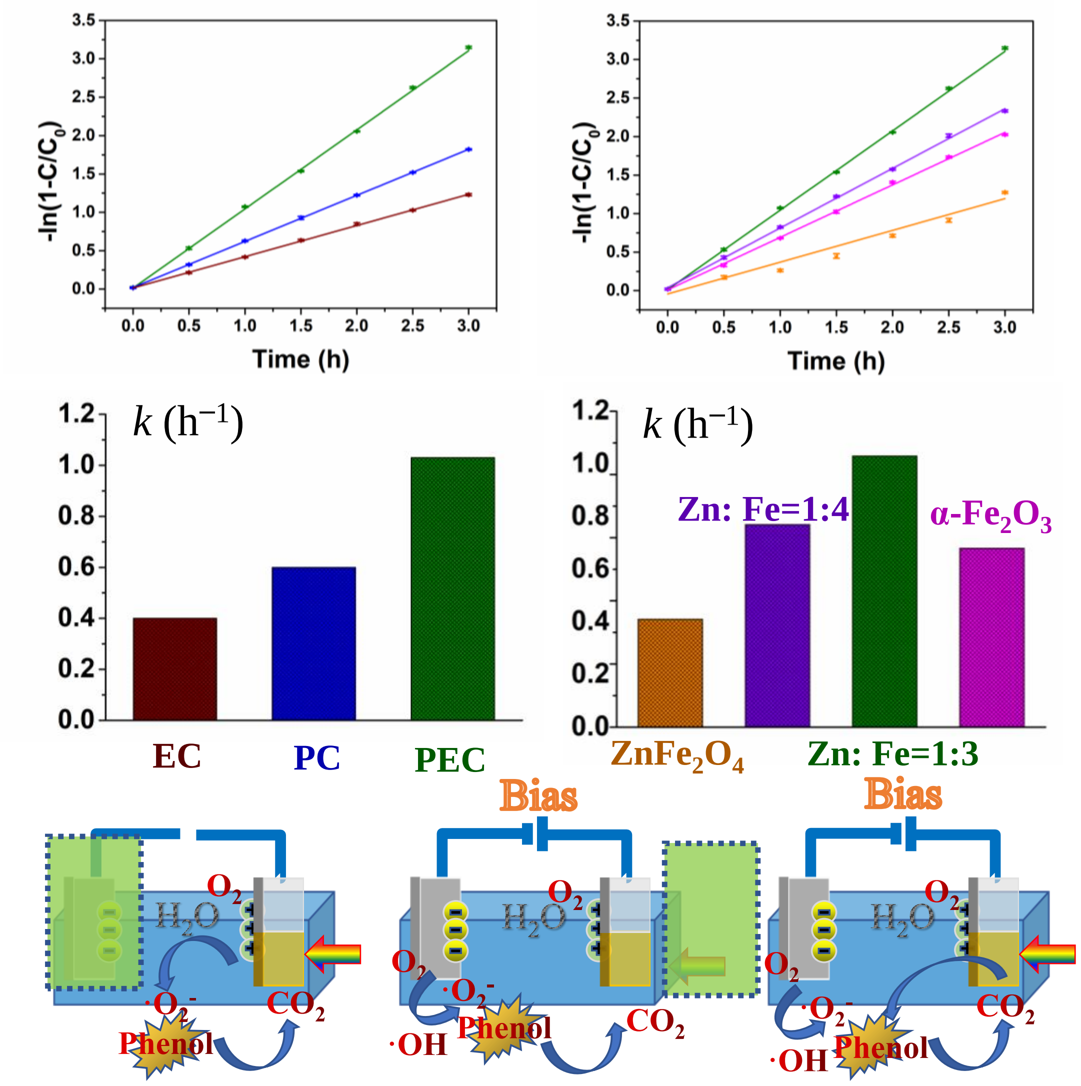

Supplementary Fig. 33. Photoelectrocatalytic process for the removal of phenol. The first order kinetics curve fitting for EC, PC and PEC removal of phenol by the Turing interface film (Zn: Fe=1:3) (AM 1.5G, 100 mW cm^{-2}, 1.6 V applied voltage). Schematic description of the mechanism for the PC, EC and PEC processes for the removal of phenol[25].

The PEC degradation rate constant of the Turing interface film (Zn: Fe=1:3) was 1.03 h^{-1} under AM 1.5 G and an applied voltage of 1.6 V, which was 1.72 times, 2.28 times higher than PC and EC, respectively (Supplementary Fig. 33).

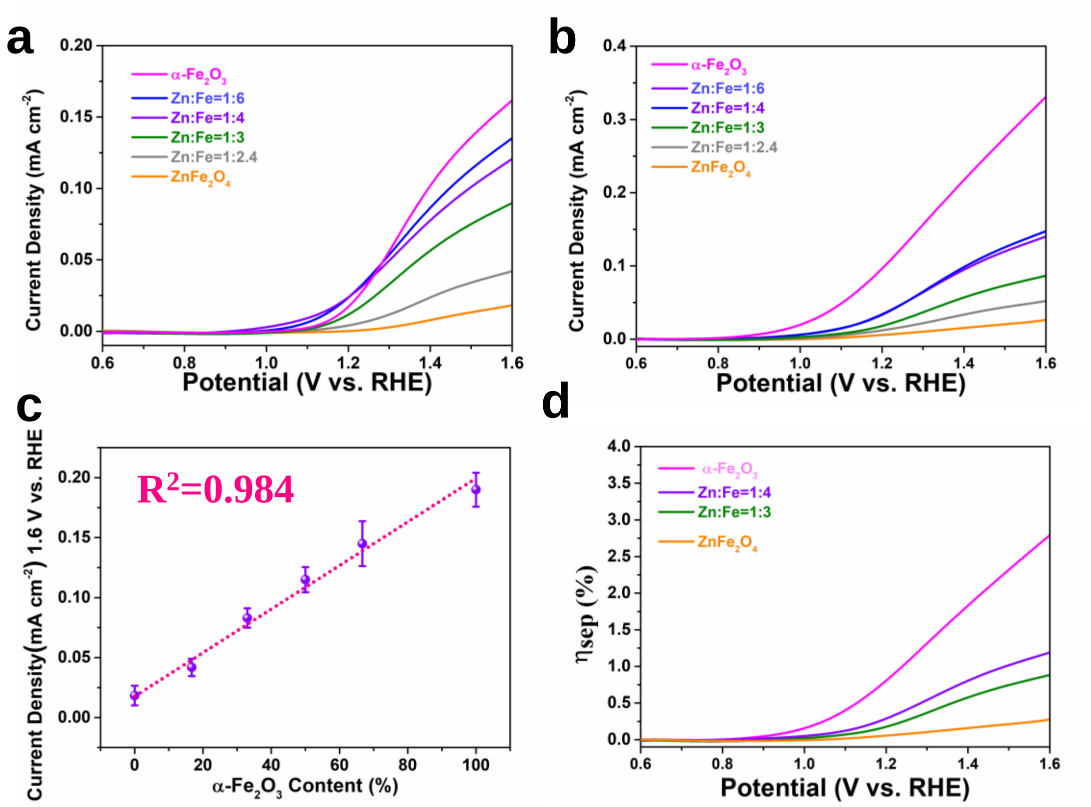

Supplementary Fig. 34. Photoelectrochemical activities for the conventional dual-phase interface films. a, Current density-potential profiles of the conventional dual-phase interface films under AM 1.5 G illumination (100 mW cm^{-2}) in 1 M NaOH electrolyte. **b**, Current density-potential profiles of the conventional dual-phase interface films under AM 1.5 G illumination (100 mW cm^{-2}) in 1 M NaOH electrolyte with 0.1 M Na_2SO_3. **c**, Curves of relationship between photocurrents at 1.6 V vs. RHE and α-Fe_2O_3 content. **d**, Charge separation efficiency of the photoelectrodes.

The performance of the conventional dual-phase interface film prepared by physical mixing and then electrophoretic deposition was shown (Supplementary Fig. 34a and b). According to the performance, the linear relationship diagram is drawn (Fig. Supplementary Fig. 34c), which can be observed that its performance increases with the α-Fe_2O_3 content. It is obviously different from the Turing interface film, mainly due to the low interface quality, which makes the built-in electric field weaker.

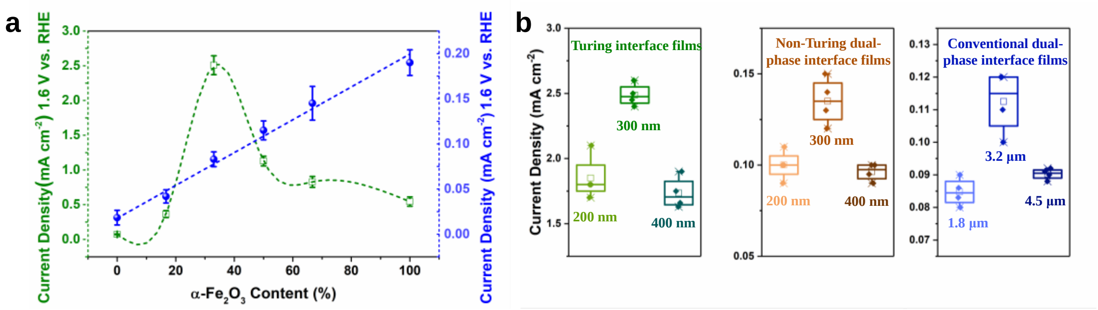

Supplementary Fig. 35. The relationship between photocurrent and α-Fe_2O_3 content and film thickness. a, Curves of relationship between photocurrents at 1.6 V vs. RHE and α-Fe_2O_3 content (the Turing interface films and the conventional dual-phase interface films). **b**, Curves of relationship between photocurrents at 1.6 V vs. RHE and film thickness.

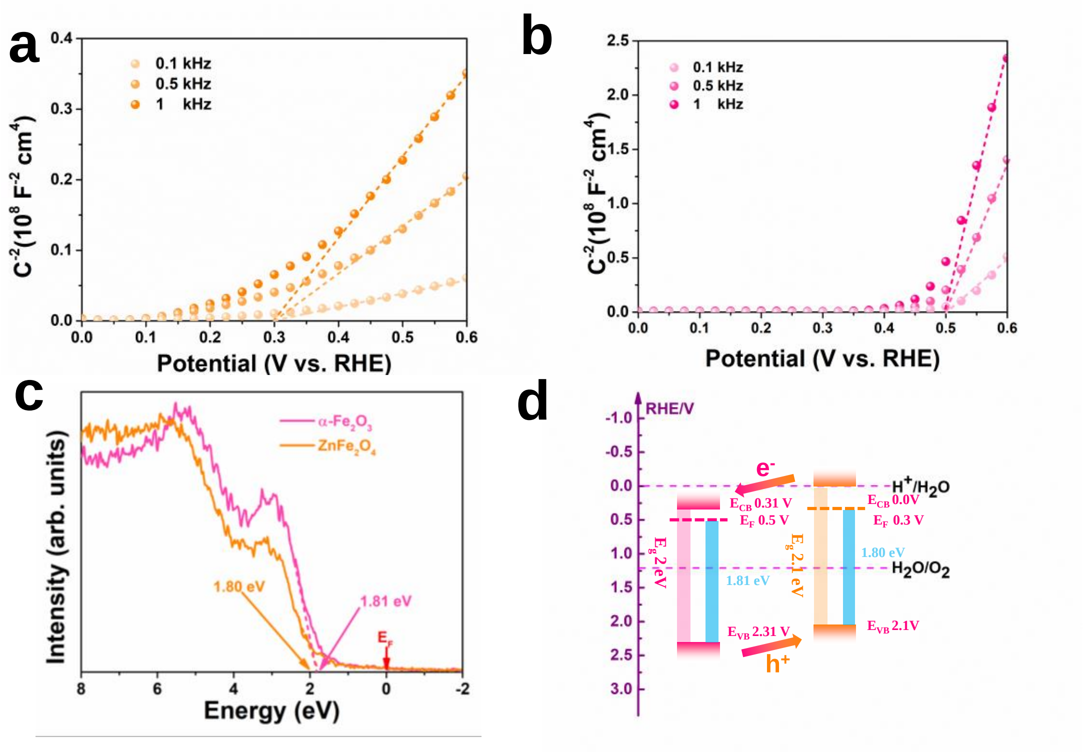

Supplementary Fig. 36. Band edge position. a, Mott-Schottky plot of $ZnFe_2O_4$. **b**, Mott-Schottky plot of α-Fe_2O_3. **c**, Valence band spectra of α-Fe_2O_3 and $ZnFe_2O_4$. **d**, Band structures of $ZnFe_2O_4$ and α-Fe_2O_3.

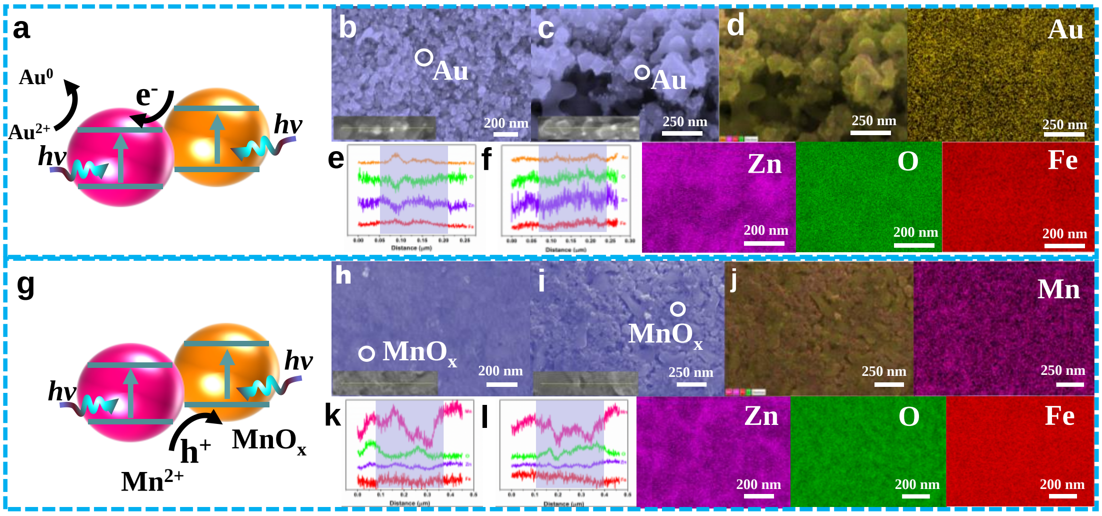

Supplementary Fig. 37. Selective photodeposition. a–f, Photoreductive deposition of Au on α-Fe_2O_3. **g–l**, Photooxidative deposition of MnO_x on $ZnFe_2O_4$.

In order to deeply understand the specific positions of the conduction band and valence band of semiconductors. Mott Schottky is used to determine the positions of the Fermi energy levels of each semiconductor (Supplementary Fig. 36a and b), and the difference between the valence band top and Fermi energy levels is determined with the help of the valence band spectrum of XPS (Supplementary Fig. 36c), then the position of the conduction band bottom is determined according to its band gap (Supplementary Fig. 36d). From the perspective of thermodynamics, they can be combined into a typical type II heterojunction, which significantly prevents the electron-hole recombination. Spatial separation of electrons and holes has also been confirmed by selective photo deposition experiments (Supplementary Fig. 37). It can be observed that photoreduced Au is deposited on α-Fe_2O_3 (Supplementary Fig. 37e and f). Moreover, MnO_x is deposited on $ZnFe_2O_4$ (Supplementary Fig. 37k and l).

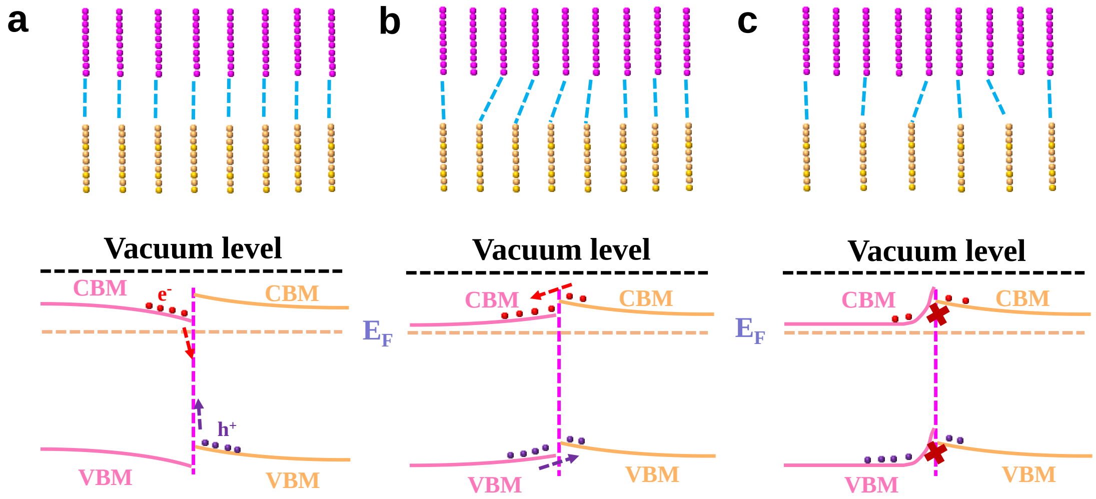

Supplementary Fig. 38. Interface states. a, Balanced energy band diagram of n-n heterojunction without interface states (coherent interface). **b**, Schematic diagram of the energy band of the n-n heterojunction when the interface state is considered (semi-coherent interface). **c**, Schematic diagram of the energy band of the n-n heterojunction when the interface states are considered (incoherent interface).

The schematic diagrams of the energy band structure including interface states were shown (Supplementary Fig. 38). There is a strict lattice match between n-type semiconductors and the balance of the Fermi level is reached, the energy band with a smaller crystallographic constant will bend downwards, thus forming the interface (Supplementary Fig. 38a), which makes the electrons and holes generated between the semiconductors form a partial recombination. Although the separation of carriers can also be achieved, the carriers cannot be employed efficiently. For the incoherent interface, due to the acceptor effect of the dangling bond, the band with the smaller crystallographic constant will be bent upward, which will increase the carrier transmission barrier, which is also disadvantageous (Supplementary Fig. 38c). The semi-coherent interface makes the acceptor action of the dangling bond form an upward band bending. The separation of carriers will not sacrifice the partial recombination of carriers. Therefore, it provides a new idea for the design of efficient n-n heterojunction.

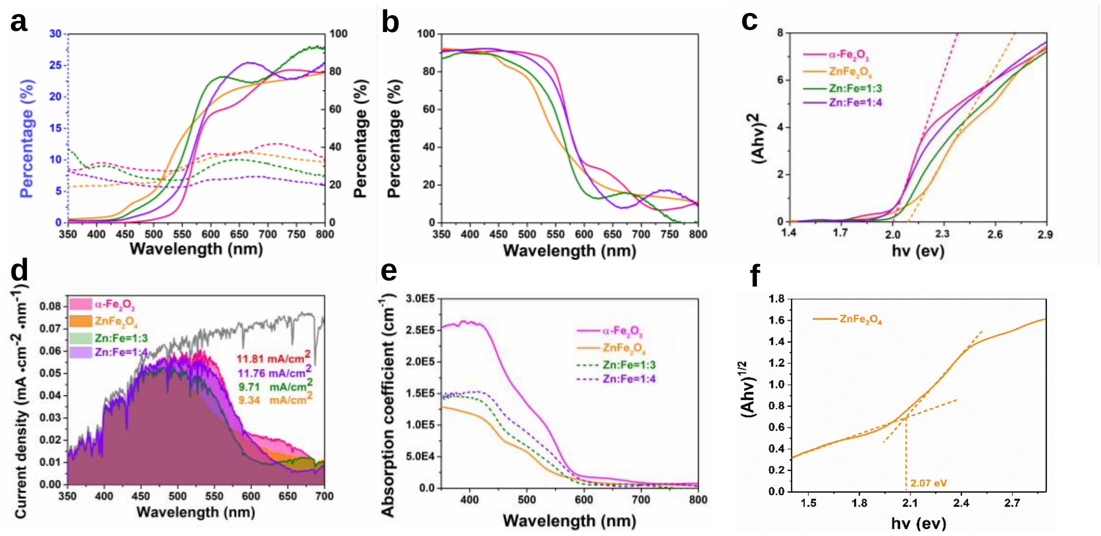

Supplementary Fig. 39. Optical properties of thin films. a, Reflection and transmission spectra. **b**, **c**, Light harvesting efficiency spectra and Tauc plot for different films. **d**, Theoretical photocurrent densities with respect to wavelengths, involving maximum photocurrents at various wavelengths for different films. **e**, Absorption coefficient spectra. **f**, Tauc plots for the direct case.

Here, the photocurrent density is the product of the theoretical photocurrent density J_{abs} under light absorption, the separation efficiency of the photogenerated charge (η_{sep}) and the injection efficiency (η_{inj}). The increase in photon utilization may be one of the reasons for the increase in the photocurrent. Thus, the absolute light absorption efficiency spectra (Supplementary Fig. 39) are studied for $ZnFe_2O_4$, Zn: Fe=1:3, Zn: Fe=1:4 and α-Fe_2O_3 films. By integrating the light harvesting efficiency spectra and the sun solar spectrum for wavelengths below 700 nm, the maximum attainable photocurrent for the present Zn: Fe=1:3 film is low comparing with α-Fe_2O_3. The influence of light absorption characteristics on the improvement of photoelectric performance is also eliminated. By calculating the light absorption coefficient (Supplementary Fig. 39e), it can also be seen that at the level of 10^5 cm^{-1}, α-Fe_2O_3 film exhibits a wider absorption. We also calculated that the value in the case of direct band gap is 2.07 eV, which is not different from that in the case of indirect band gap (Supplementary Fig. 39f).

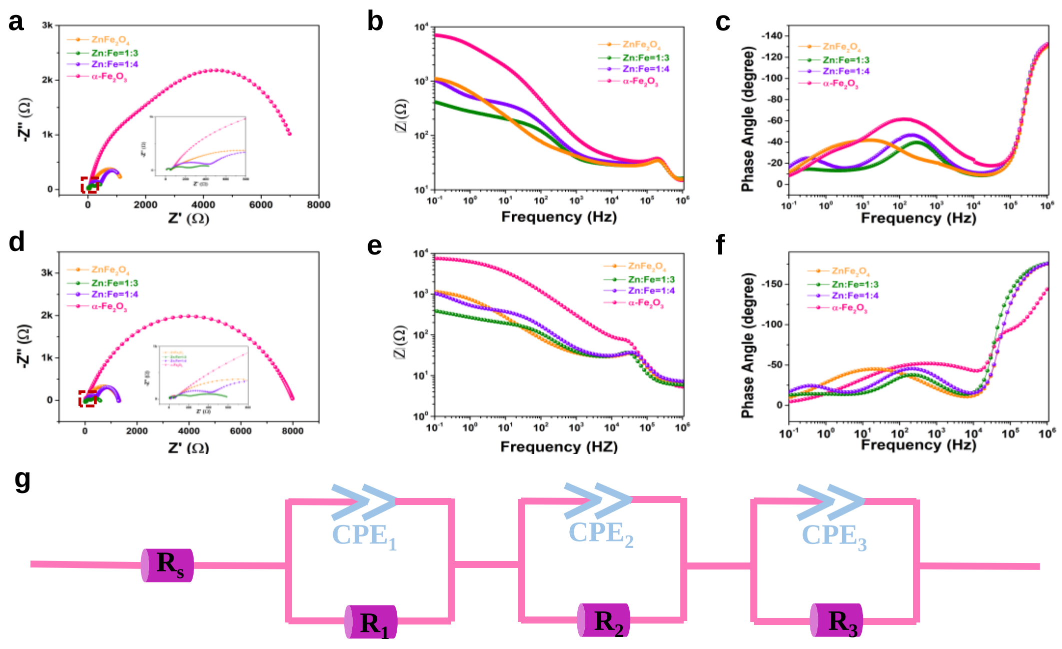

Supplementary Fig. 40. Photoelectrochemical impedance. a, PEIS spectra of different samples under illumination in 1 M NaOH electrolyte at 1.6 V vs RHE. **b**, Bode magnitude. **c**, Angle plots. **d**, The fitting results. **e**, Bode magnitude. **f**, Angle plots. **g**, Equivalent circuit used to fit the Nyquist plots.

The Nyquist impedance of $ZnFe_2O_4$, the Turing interface film (Zn: Fe=1:3), the Turing interface film (Zn: Fe=1:4) and α-Fe_2O_3 were carried out under illumination at 1.6 V vs RHE (Supplementary Fig. 40). In the equivalent circuit, Rs, resistance is associated with the electric contacts of the electrode, electrolyte, etc. R_1 is the interfacial resistance and of the FTO/bulk sample interface. CPE_1 is the constant phase element representing the capacitance of the FTO/bulk sample interface. R_2 and CPE_2 are the bulk resistance and constant phase element representing the capacitance of the bulk sample of the sample. R_3 and CPE_3 characterize the charge-transfer resistance and the surface-state capacitance at the electrode/electrolyte interfaces, respectively. According to the results of the fitting (Supplementary Table 4), the Turing interface film (Zn: Fe=1:3) has the smallest bulk resistance and the smallest charge transfer resistance under illumination, which provides direct evidence for the heterojunction formed by the high-quality interface.

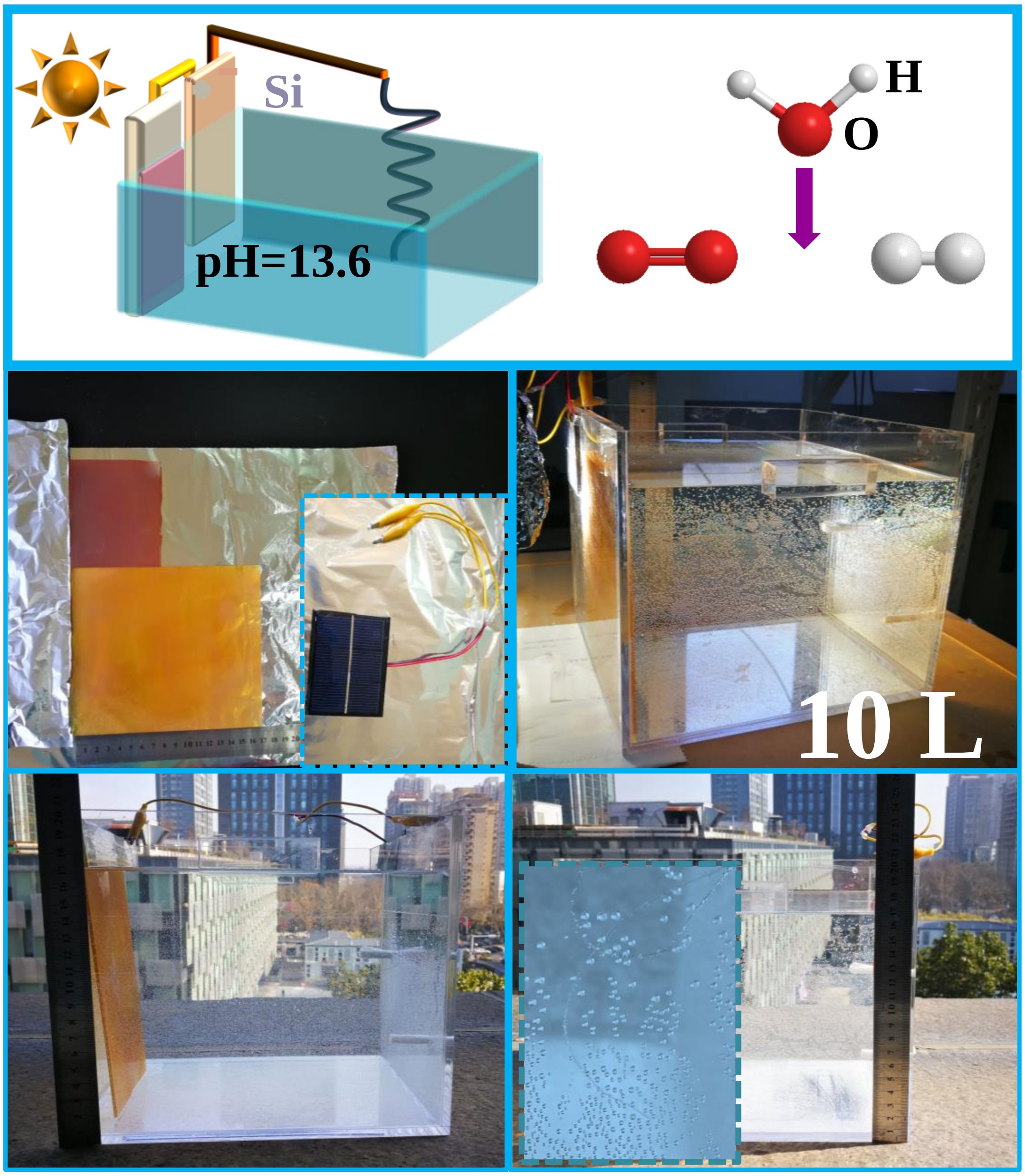

Supplementary Fig. 41. Photoelectrochemical activities. Some photos showing a commercial silicon solar cell-driven photoelectrolysis (17cm × 15cm electrode) of water (1 M NaOH, 10 L) running indoor and outdoor.

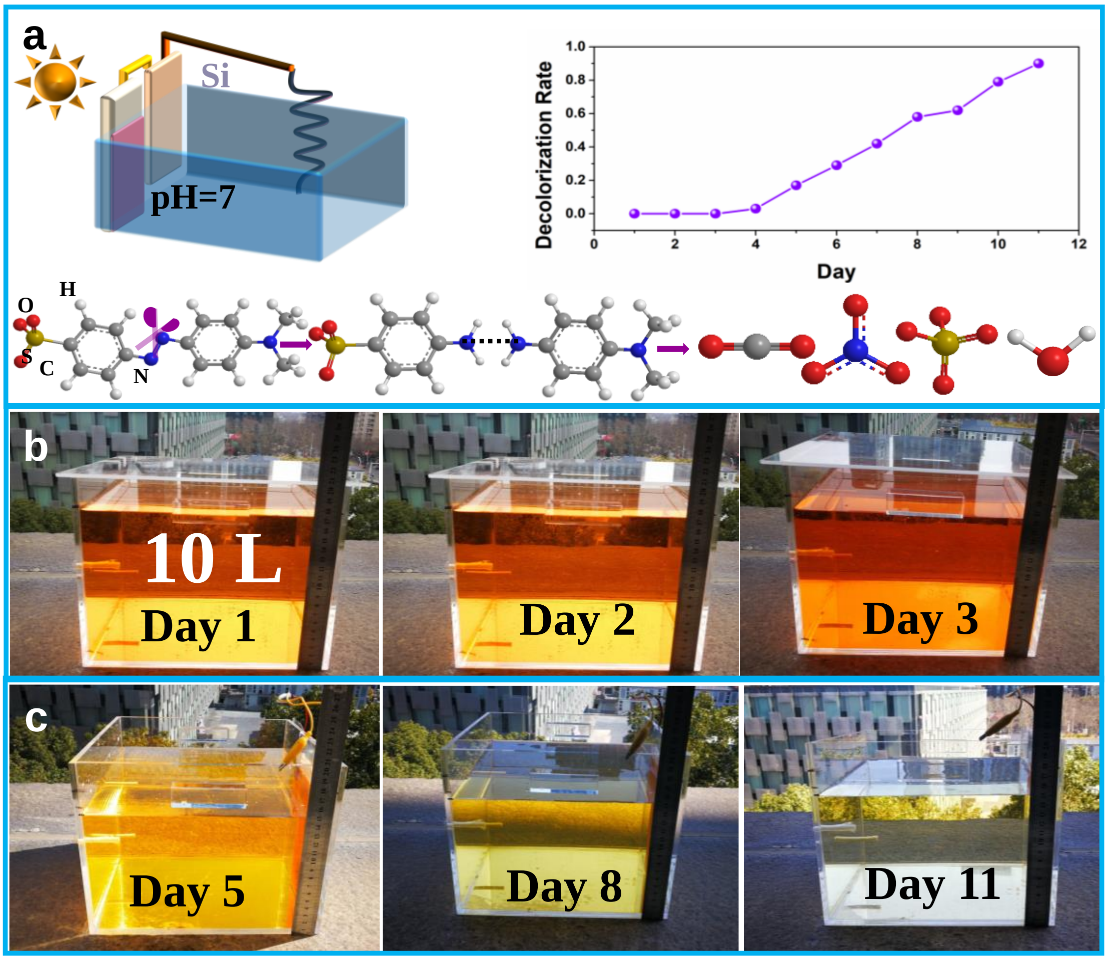

Supplementary Fig. 42. **Photoelectrochemical wastewater treatment. a**, Schematic diagram of outdoor photoelectric oxidation. **b**, Pictures show that organic pollutant not decompose outdoor without employing PEC technology for three days. **c**, Pictures show a commercial silicon solar cell-driven photoelectric oxidation (17cm×15cm electrode) of Azo wastewater (1M Na_2SO_4, 10 mg/L Azo wastewater, 10 L) running outdoor.

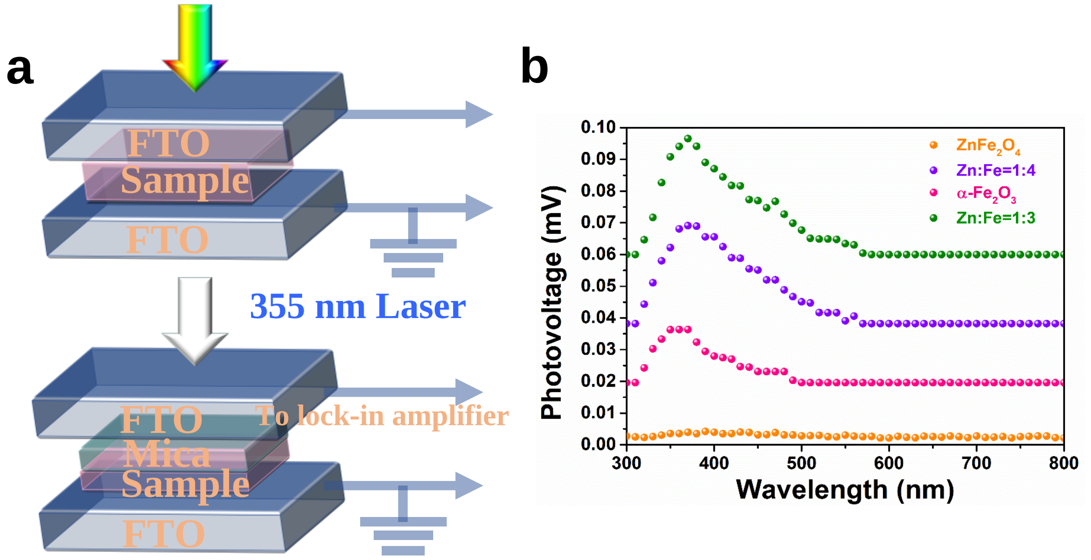

Supplementary Fig. 43. SPV and TPV. a, Schematic diagram of SPV and TPV signals. **b**, SPV.

The SPV spectra of different specimens was observed, plotted as a function of wavelength (Supplementary Fig. 43). Note that pristine $ZnFe_2O_4$ and α-Fe_2O_3 show weak SPV response in the ultraviolet region of 300–400 nm due to its intrinsic transition. However, a remarkable strong SPV signal is measured for the Turing interface film (Zn: Fe=1:3) existing stronger interaction between $ZnFe_2O_4$ and α-Fe_2O_3, which leads to generation of the electron-hole pairs efficiently separated, resulting in an improved photovoltaic response, strongly demonstrating that the significant separation and transfer of photo-generated charges appeared in spatial with the aid of strong intrinsic electric-field.

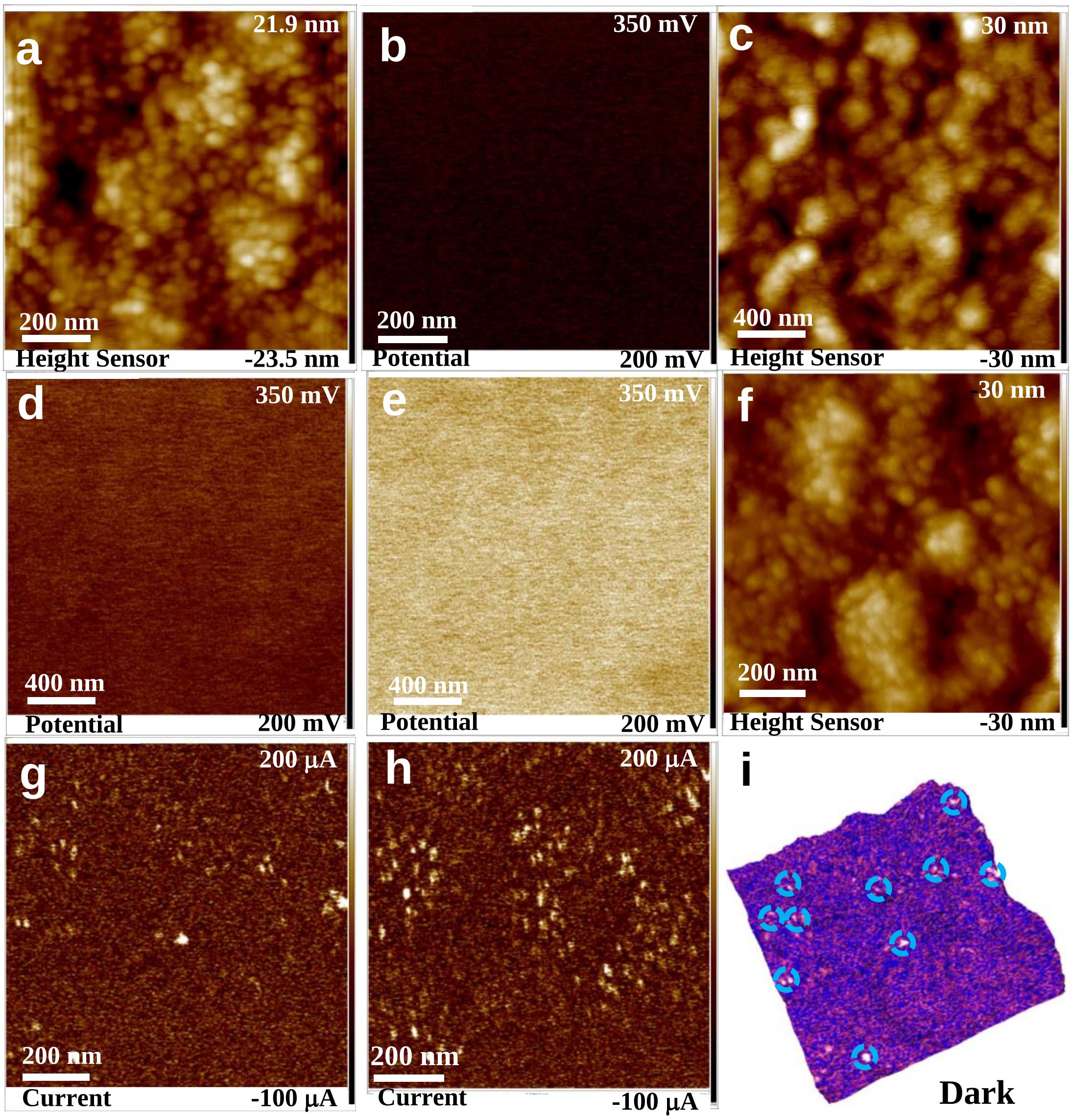

Supplementary Fig. 44. KPFM and C-AFM. **a**, Topographic AFM image of the Turing interface film (Zn: Fe=1:3) for a V_{CPD}. **b**, V_{CPD} image in the dark for the Turing interface film (Zn: Fe=1:3). **c**, AFM image of α-Fe_2O_3 film. **d**, V_{CPD} image of α-Fe_2O_3 film in the dark. **e**, V_{CPD} image of α-Fe_2O_3 film under light irradiation. **f**, Topographic AFM image of the Turing interface film (Zn: Fe=1:3) for current measurement. **g**, Current image of the Turing interface film (Zn: Fe=1:3) in the dark (1 V bias). **h**, Current image of the Turing interface film (Zn: Fe=1:3) under 405 nm light irradiation (1 V bias). **i**, Current images of the Turing interface film (Zn: Fe=1:3) overlaid with (3D) in the dark (1 V bias).

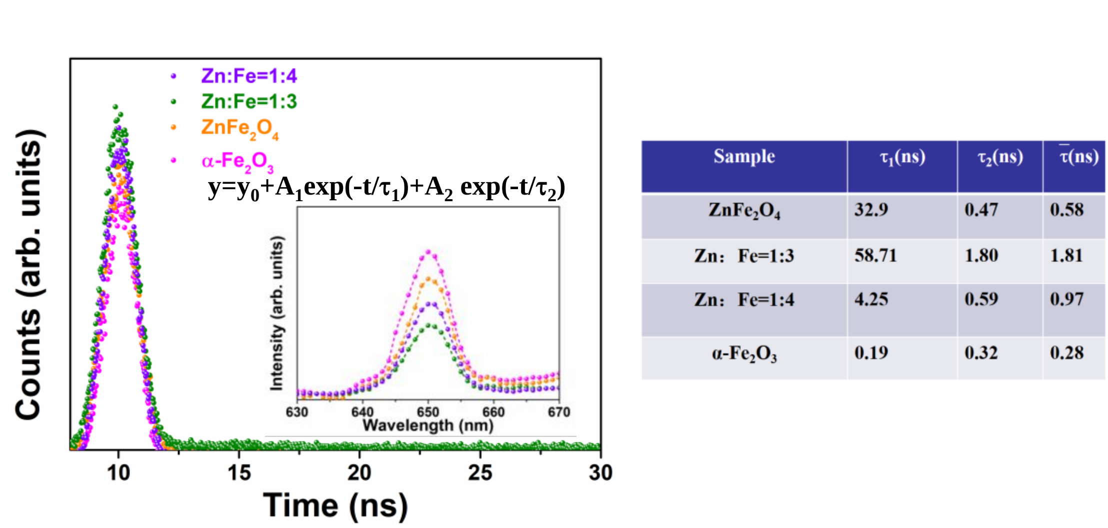

Sample	τ_1(ns)	τ_2(ns)	$\bar{\tau}$(ns)
$ZnFe_2O_4$	32.9	0.47	0.58
Zn：Fe=1:3	58.71	1.80	1.81
Zn：Fe=1:4	4.25	0.59	0.97
α-Fe_2O_3	0.19	0.32	0.28

Supplementary Fig. 45. PL and TRPL. Time-resolved PL spectra of different films. Inset shows the steady-state PL spectra.

The room-temperature photoluminescence (PL) and time-resolved transient photoluminescence delay (TRPL) were employed to further elucidate the charge carrier dynamics of the photoelectrodes, which show a narrow emission band centered at ≈650 nm due to the similar bandgap of $ZnFe_2O_4$ and α-Fe_2O_3. The Turing interface film (Zn: Fe=1:3) displays the lowest PL intensity and much longer lifetime compared with other samples, which shows high carrier separation efficiency and charge transfer efficiency[26].

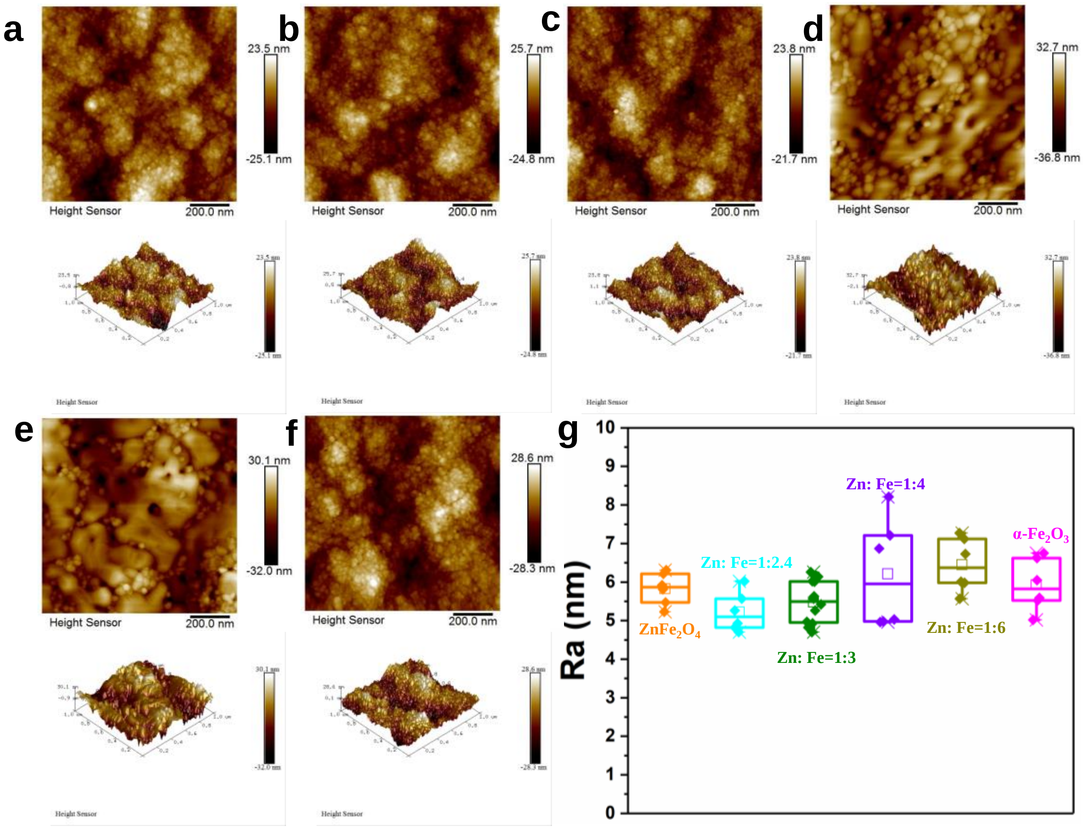

Supplementary Fig. 46. AFM micrographs (2D and 3D) prepared from $Fe[C_5H_7O_2]_3$. **a**, $ZnFe_2O_4$ film. **b**, Zn: Fe=1:2.4 film. **c**, Turing interface film (Zn: Fe=1:3). **d**, Turing interface film (Zn: Fe=1:4). **e**, Turing interface film (Zn: Fe=1:6). **f**, α-Fe_2O_3 film. **g**, Average surface roughness (Ra).

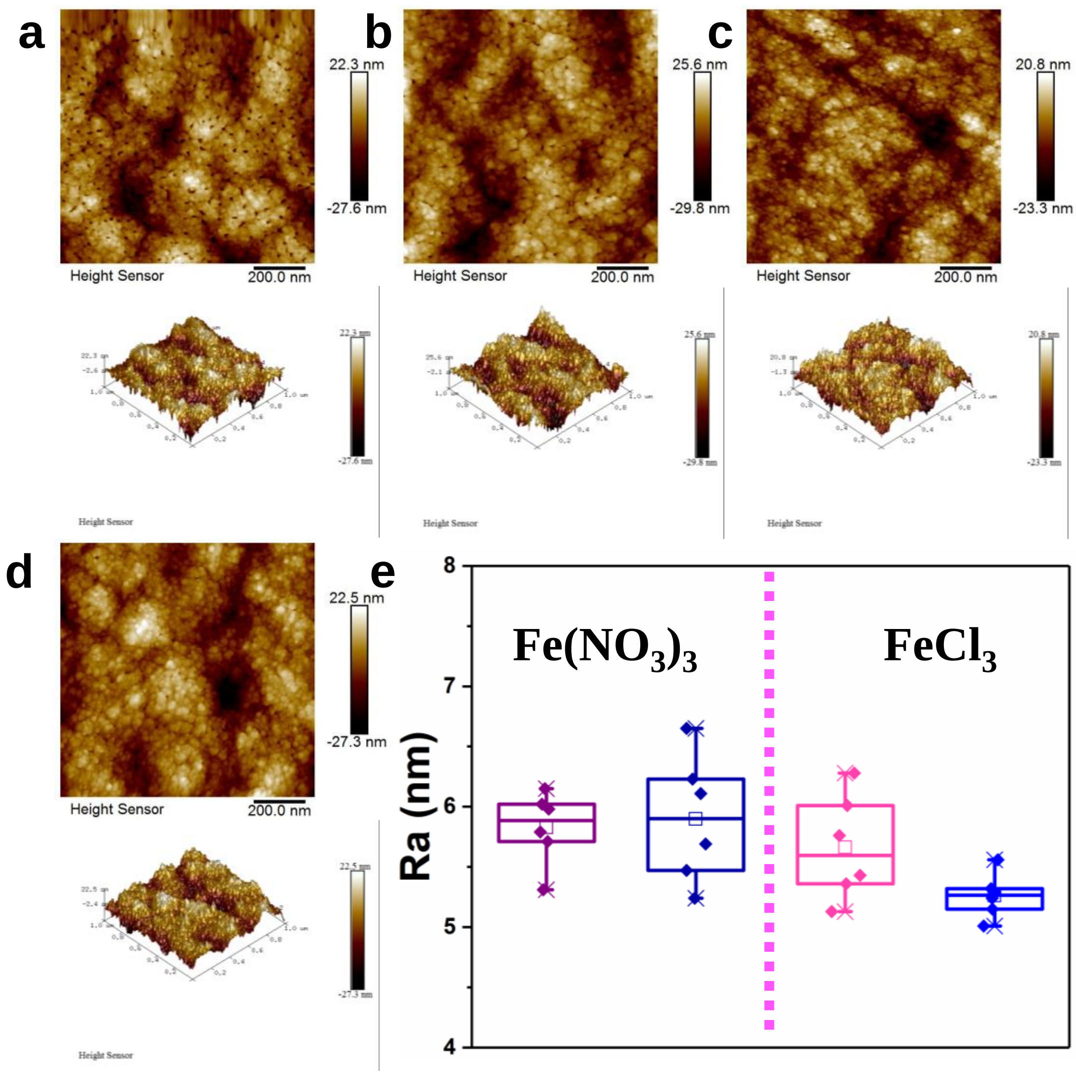

Supplementary Fig. 47. AFM micrographs (2D and 3D) of non-Turing dual-phase interface films (Zn: Fe=1:3 and Zn: Fe=1:4) prepared from inorganic metal source. **a**, **b**, $Fe(NO_3)_3$. **c**, **d**, $FeCl_3$. **e**, Average surface roughness (Ra).

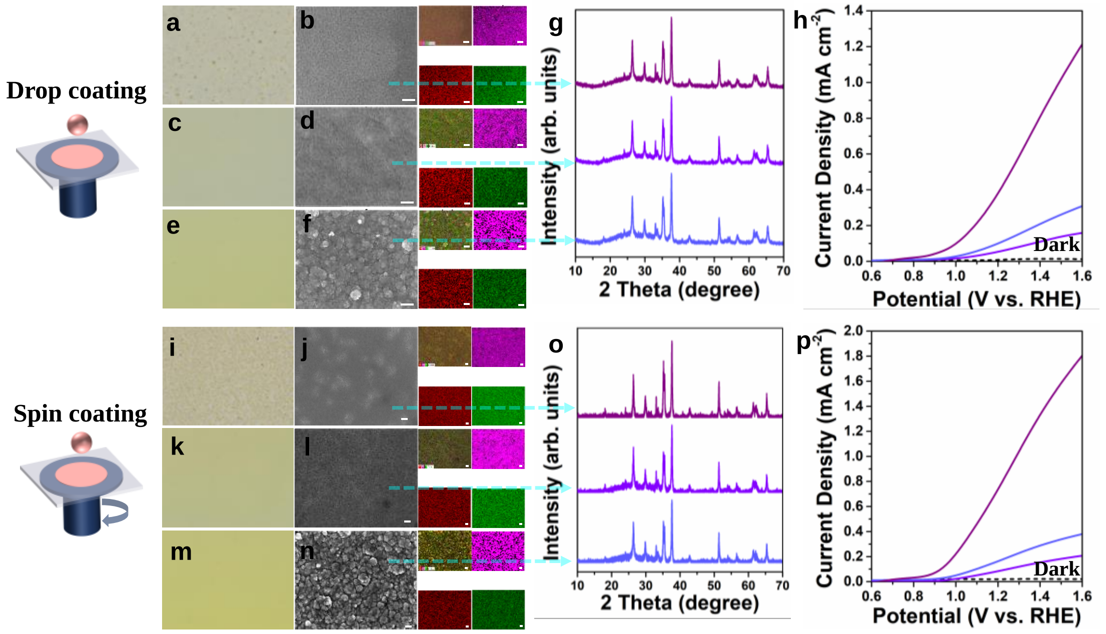

Supplementary Fig. 48. Different fabrication strategies were used to verify the homogeneous solution characteristics of Turing structure and non-Turing structure. Drop coating: a, The shape evolution of droplets (Zn: Fe=1:3, $Fe[C_5H_7O_2]_3$). **b**, SEM image and elemental mapping of Turing structure film for above solution. **c**, The shape evolution of droplets (Zn: Fe=1:3, $Fe(NO_3)_3$). **d**, SEM image and elemental mapping of non-Turing dual-phase interface film for above solution. **e**, The shape evolution of droplets (Zn: Fe=1:3, $FeCl_3$). **f**, SEM image and elemental mapping of non-Turing dual-phase interface films for above solution. **g**, XRD patterns for Turing structure film and non-Turing structure film. **h**, Current density-potential profiles of Turing structure film and non-Turing structure film**. Spin coating: i,** The shape evolution of droplets (Zn: Fe=1:3, $Fe[C_5H_7O_2]_3$). **j**, SEM image and elemental mapping of Turing structure film for above solution. **k**, The shape evolution of droplets (Zn: Fe=1:3, $Fe(NO_3)_3$). **l**, SEM image and elemental mapping of non-Turing dual-phase interface films for above solution. **m**, The shape evolution of droplets (Zn: Fe=1:3, $FeCl_3$). **n**, SEM image and elemental mapping of non-Turing dual-phase interface films for above solution. **o**, XRD patterns for Turing structure film and non-Turing structure film. **p**, Current density-potential profiles of Turing structure film and non-Turing structure film**.** Scale bars: **b**, **d**, **f**, **j**, **l**, **n**, 200 nm.

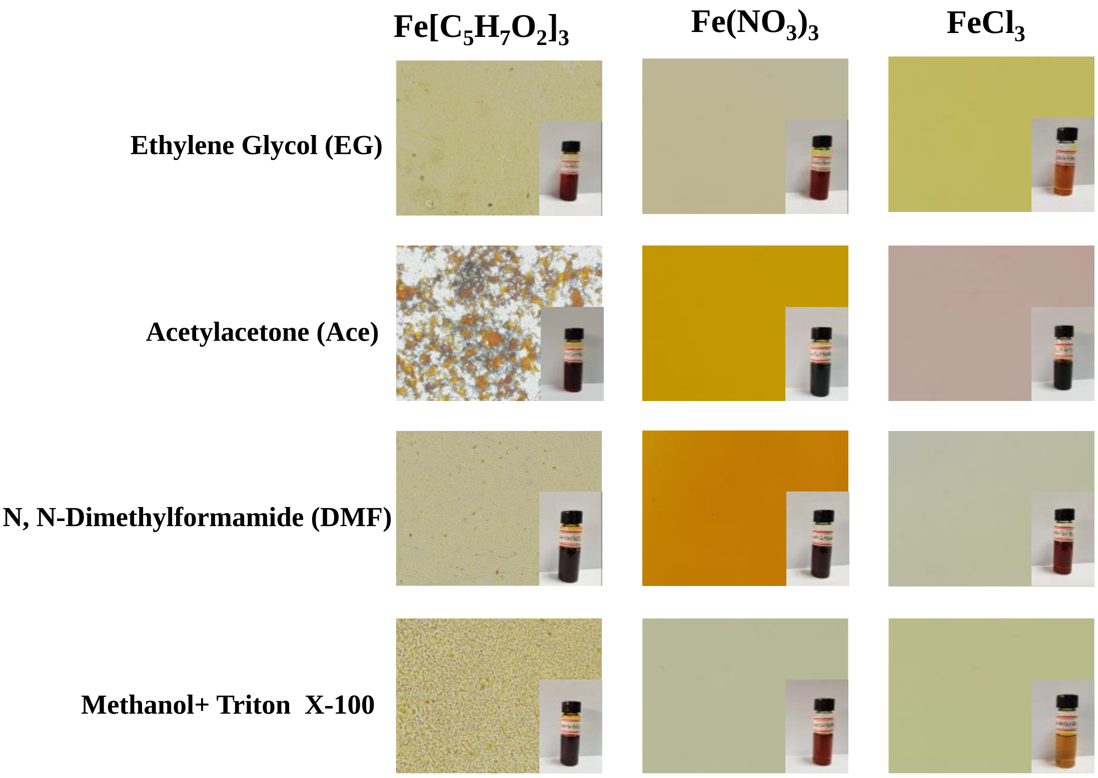

Supplementary Fig. 49. The shape evolution of droplets with different solvents.

In order to further verify that the formation of Turing structure is determined by intrinsic property of the solution rather than an artifact that can appear due to the fabrication process, we used the drop coating and spin coating technique to prepare high-performance Turing structure films. The above conclusions are furtherly summarized from Supplementary Fig. 48. Once the difference of diffusion coefficient of related substances in homogeneous solution is modulated, Turing structure films can be obtained by either drop coating or spin coating process. Furthermore, the Turing structure films performances were found to be better than that of non-Turing structure films. Nevertheless, there is a gap between the properties of the films prepared by the above process and the films prepared by spray pyrolysis process. It is mainly due to the characteristics of spin coating and drop coating process, and we have not optimized the technology. For the drop coating process, although Turing structure film can also be prepared, the film area is usually small and the thickness is not easy to control. For the spin coating technique, the viscosity of the solution needs to be considered. If the process is optimized, a film with performance comparable to that of the spray pyrolysis film can be obtained. Accordingly, here we try to improve the spin coating technique

and give researchers more inspiration.

By comparing the droplet evolution of mixed solutions composed of iron sources and $Zn(CH_3COO)_2$ (Supplementary Fig. 49), we can draw the following conclusion: $Fe(NO_3)_3$ and $FeCl_3$ in four solvents show a diffusion coefficient difference close to 1, which does not appear regular spots or strips structure. $Fe[C_5H_7O_2]_3$ is used as a source of iron, and ethylene glycol or acetylacetone as a solvent, which will not form a Turing structure. While it exhibits a regular structure in DMF solution. Simultaneously, introducing small amount of surfactant into the methanol solution does not affect the diffusion coefficient of the ions, so as to adjust the viscosity of the solution, it will be easier to spin coating into a high-quality film. This further confirms that the Turing structures of film is the intrinsic property of the solution.

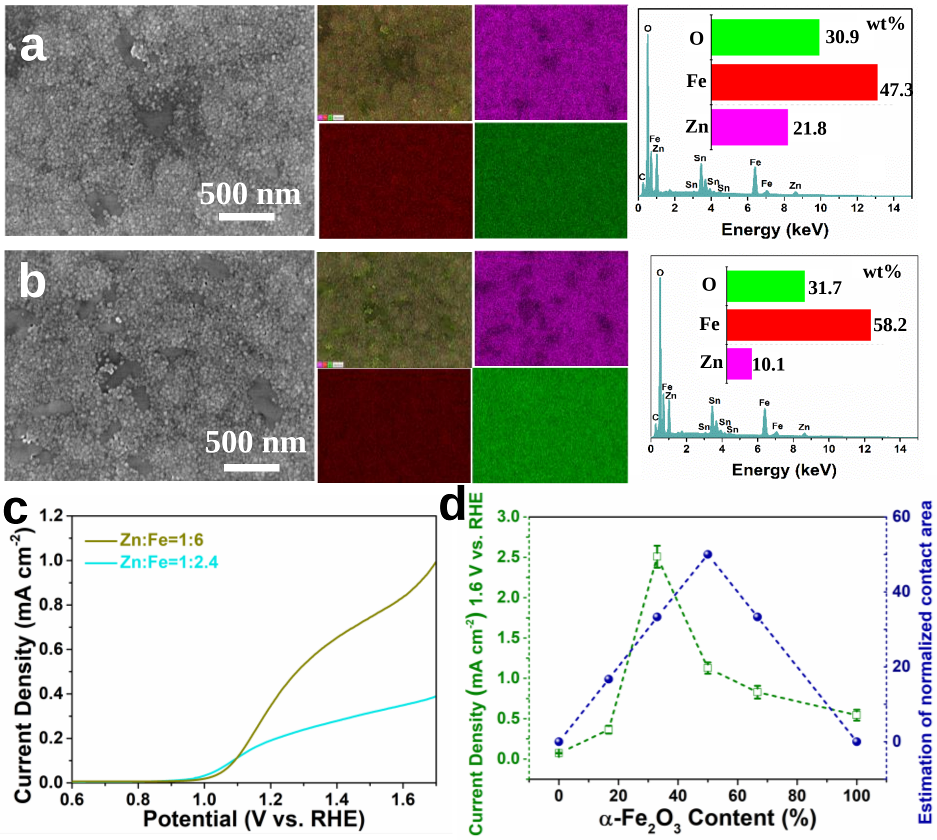

Supplementary Fig. 50. Elemental mappings. a, Elemental mappings of Zn: Fe=1:2.4 film. **b**, Zn: Fe=1:6 film. **c**, Current density-potential profiles of different films measured under AM 1.5 G illumination (100 m W cm^{-2}). **d**, Photocurrent density (1.6 V vs. RHE, AM 1.5G, 100 mW cm^{-2}) and the estimation of normalized contact area depending on the α-Fe_2O_3 content.

In order to further clarify the relationship of the concentration gradient, the other thin films with a concentration ratio were prepared (Supplementary Fig. 50a and b). The photocurrent density (AM 1.5G, 100 mW cm^{-2}) at 1.6 V vs. RHE and the estimation of normalized contact area depending on the α-Fe_2O_3 content are shown (Supplementary Fig. 50c and d). As expected, the variation trend is very similar, which also provides more sufficient evidence that the increase of contact area can more effectively promote the Turing interface. Based on the above analysis, it can be concluded that the effective contact and construction of the interface can achieve the maximum effective separation of photogenerated electrons and holes. However, which is indeed neglected by many studies.

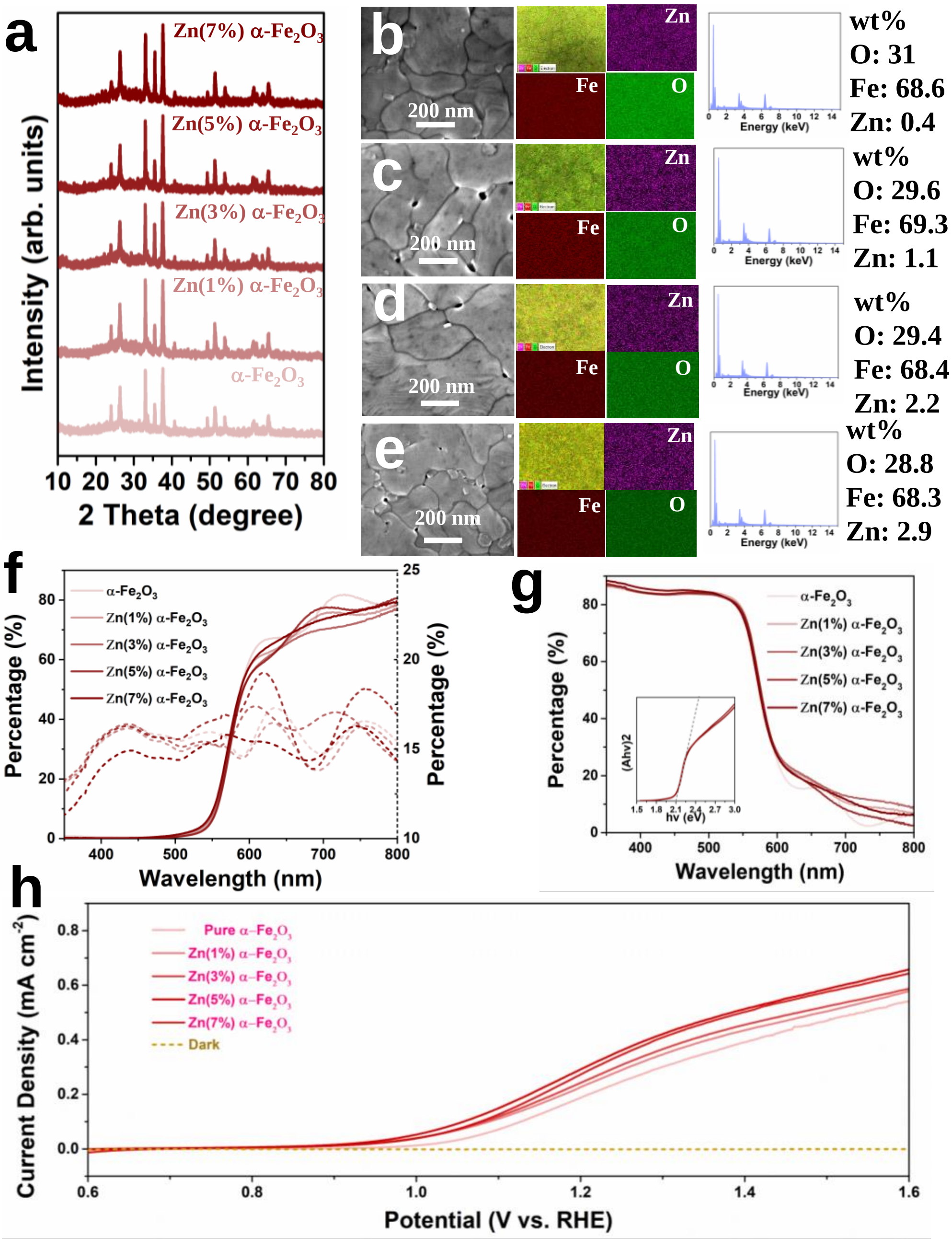

Supplementary Fig. 51. SEM images, elemental mappings and photoelectrochemical activities of Zn-doped α-Fe_2O_3 films. a, XRD patterns for the Zn-doped α-Fe_2O_3 films. **b–e**, Elemental mappings of the Zn-doped α-Fe_2O_3 films. **f**, Reflection and transmission spectra of the Zn-doped α-Fe_2O_3 films. **g**, Light harvesting efficiency spectra and Tauc plot for different films. **h**, Current density-potential profiles of the Zn-doped α-Fe_2O_3 films under AM 1.5 G illumination (100 mW cm^{-2}).

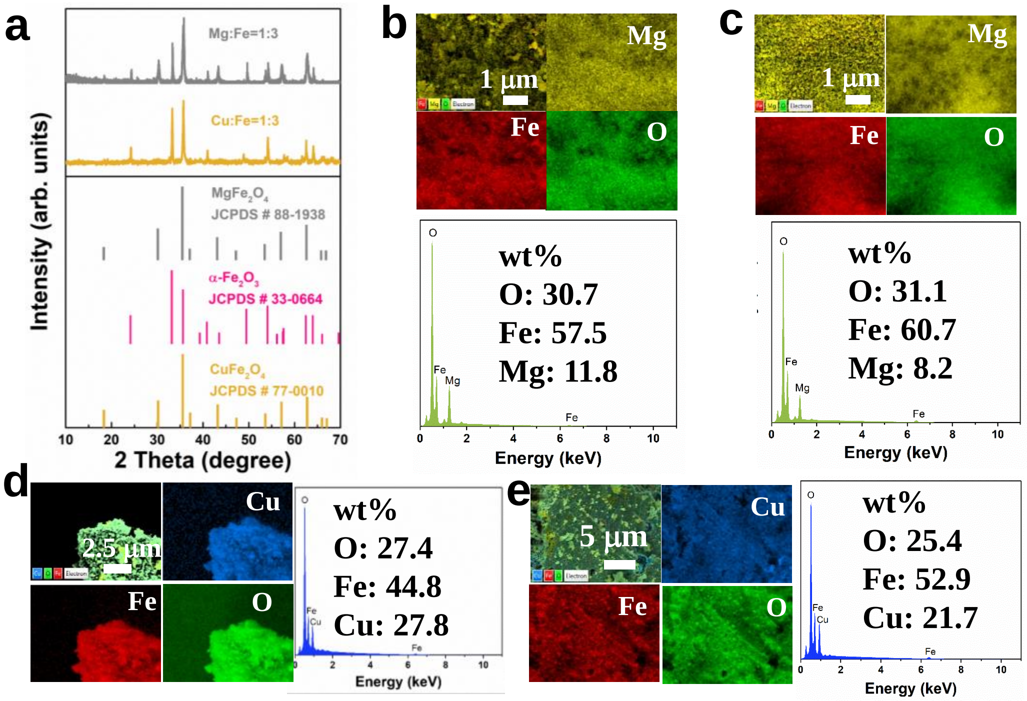

Supplementary Fig. 52. XRD patterns, SEM images and elemental mappings. a, XRD patterns for the Mg: Fe=1:3 and Cu: Fe=1:3 powder. **b**, **c**, Elemental mappings of $MgFe_2O_4$. **d**, **e**, Elemental mappings of $CuFe_2O_4$.

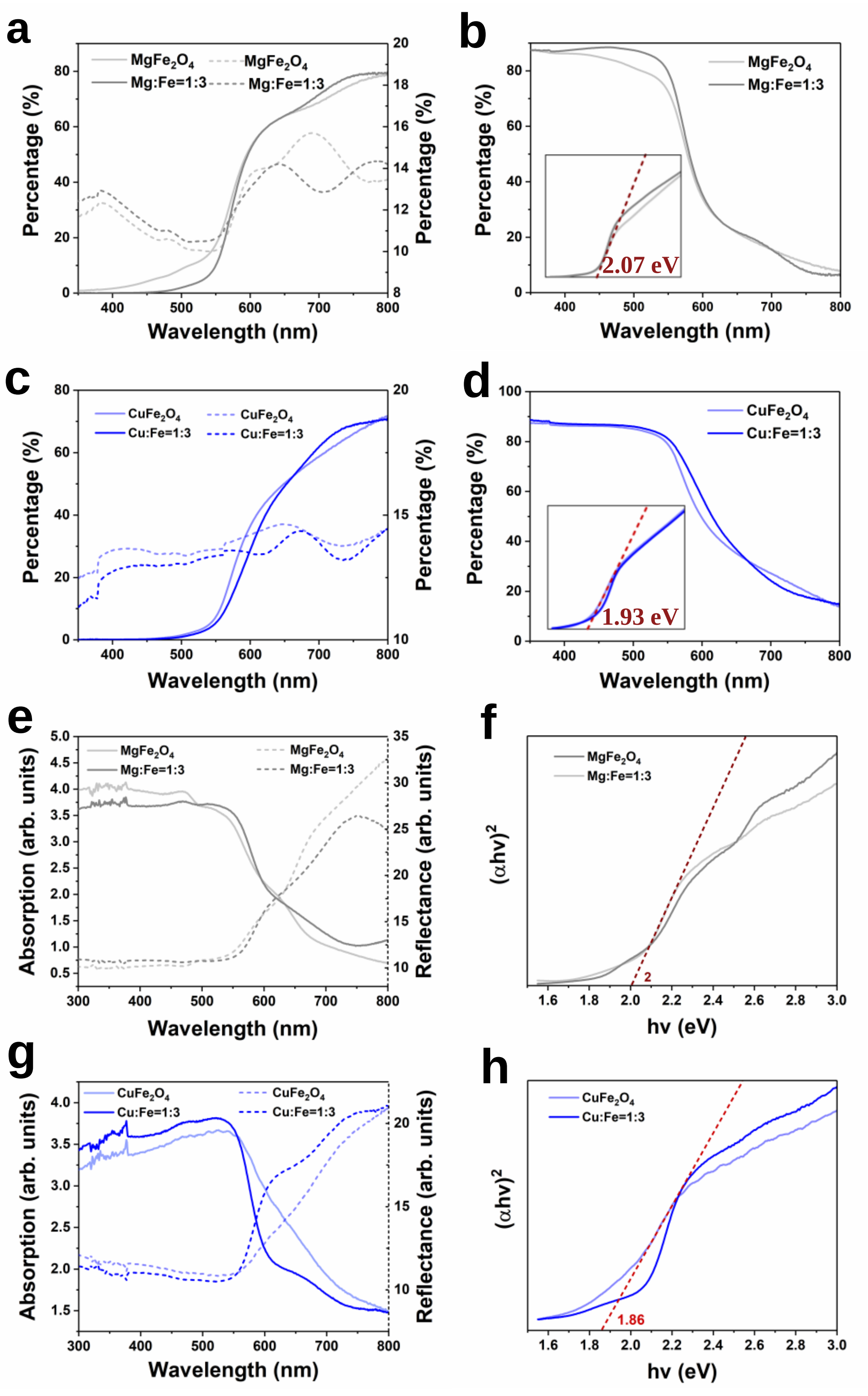

Supplementary Fig. 53. Optical properties of the samples. a–d, Ultraviolet-visible light absorption spectra of and Tauc plots for the $MgFe_2O_4$, Mg: Fe=1:3, $CuFe_2O_4$ and Cu: Fe=1:3 powder. **e–h**, Reflection and transmission spectra, light harvesting efficiency spectra and for $MgFe_2O_4$, Turing interface film (Mg: Fe=1:3), $CuFe_2O_4$ and Turing interface film (Cu: Fe=1:3).

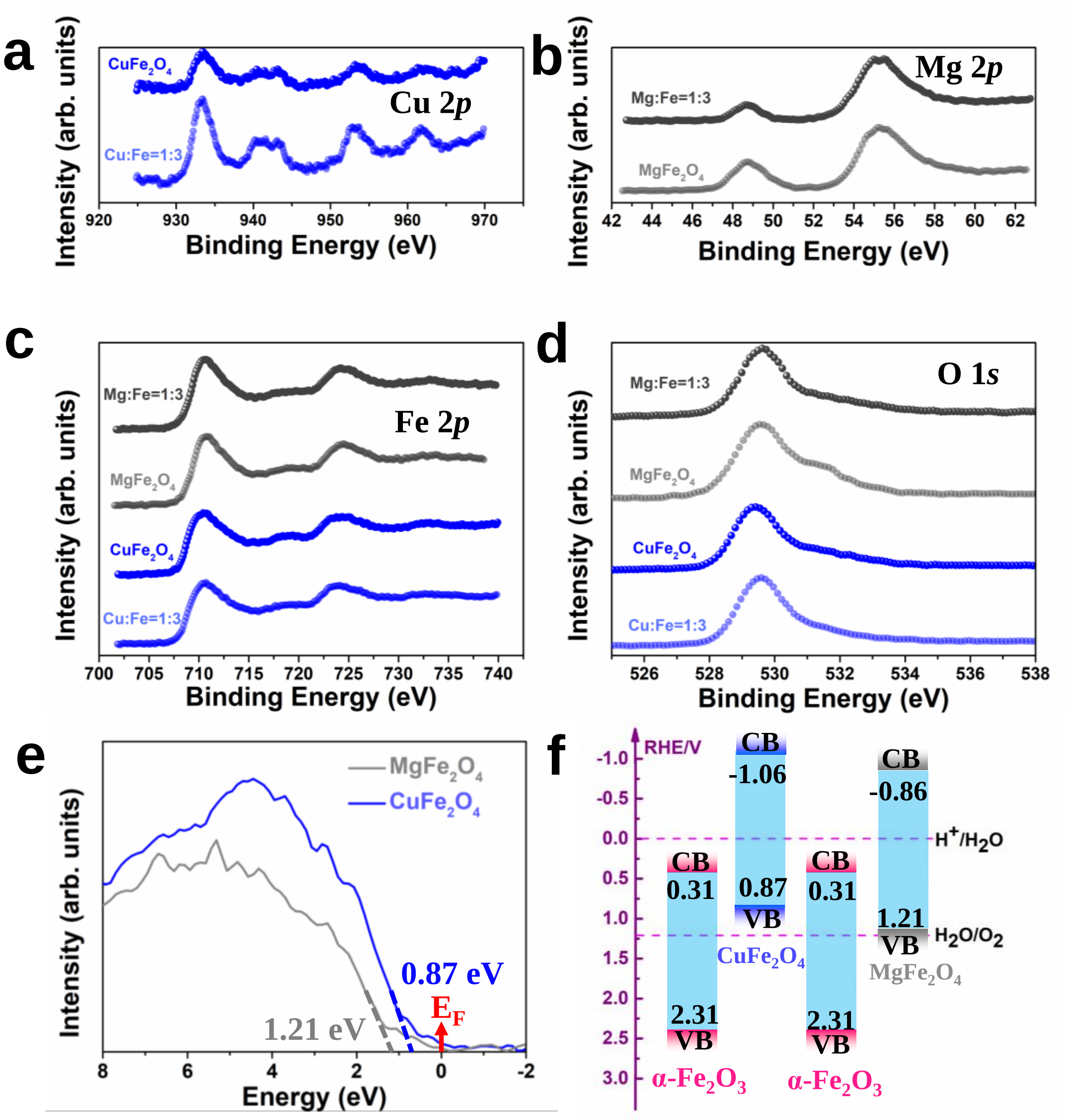

Supplementary Fig. 54. XPS spectra and band edge position of the samples. a, Cu 2*p*. **b**, Mg 2*p*. **c**, Fe 2*p*. **d**, O 1*s*. **e**, Valence band spectra of $MgFe_2O_4$ and $CuFe_2O_4$ films. **f**, Band structures of $MgFe_2O_4$ and $CuFe_2O_4$.

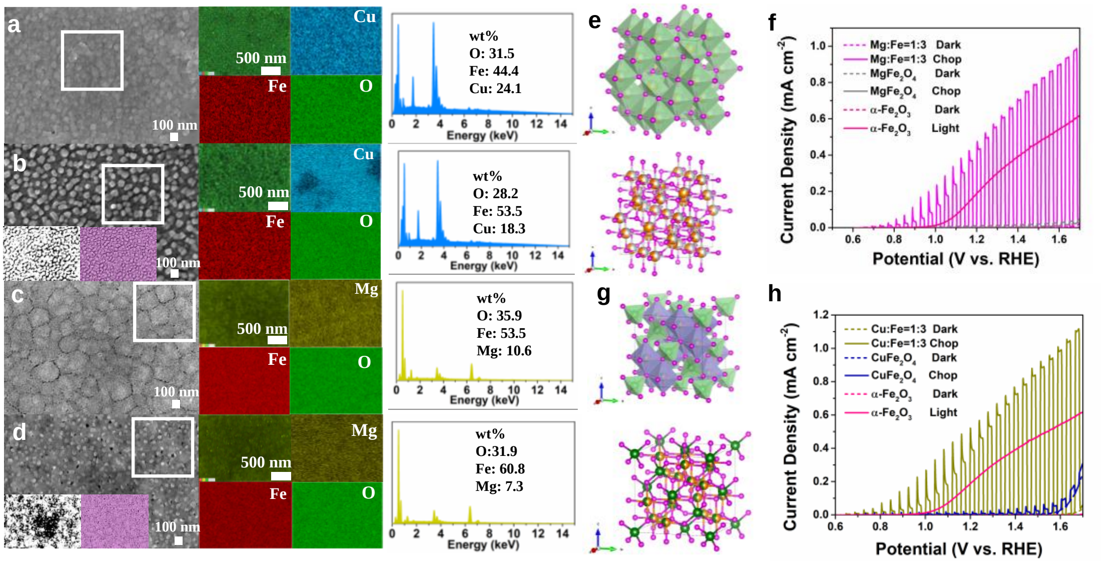

Supplementary Fig. 55. SEM images and elemental mappings of Turing interface film. a, $CuFe_2O_4$. **b**, Turing interface film **(**Cu: Fe=1:3**)**, the illustration shows the pseudo-color mode. **c**, $MgFe_2O_4$. **d**, Turing interface film (Mg: Fe=1:3), the illustration shows the pseudo-color mode. **e**, Crystallographic images of $MgFe_2O_4$. **f**, *J-V* curves of photoelectrode films of $MgFe_2O_4$. **g**, Crystallographic images of $CuFe_2O_4$. **h**, *J-V* curves of photoelectrode films of $CuFe_2O_4$.

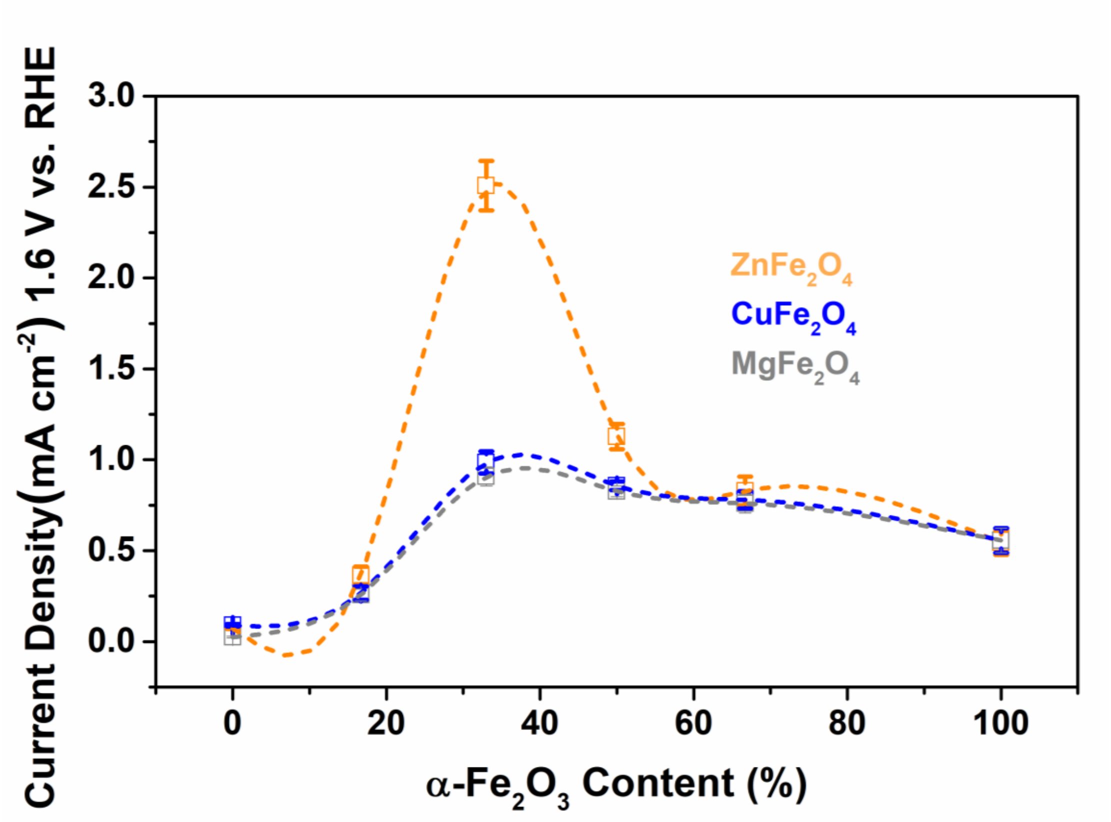

Supplementary Fig. 56. Curves of relationship between photocurrents at 1.6 V vs. RHE and α-Fe_2O_3 content.

Supplementary Table 1. Comparison of our films to other iron-based films in recent years.

Materials	Solution	Cocatalyst	Light Source	Current Density at 1.6 V_{RHE} (mA cm^{-2})	Separation Efficiency	Ref.
Turing interface film (Zn: Fe=1:3)	1M NaOH	None	AM 1.5G (1sun) Newport Sol3A Class AAA simulator.	2.56	44.1% at 1.6 V_{RHE}, 37.1% at 1.23 V_{RHE}	This work
Turing interface film (Cu: Fe=1:3)				1.01	None	
Turing interface film (Mg: Fe=1:3)				0.96	None	
$ZnFe_2O_4$(H)	1M NaOH	Nickel-iron oxide	A filtered 450 W Xenon-arc lamp (Muller Elektronik), calibrated to provide 1 Sun illumination	1.7	35% at 1.6 V_{RHE}	17
$ZnFe_2O_4/Al_2O_3$	1M NaOH	None	An array of white LED (Cree X Lamp MC-E Color) (≈0.66 sun)	~0.8	None	27
$ZnFe_2O_4$(P)+M	1M NaOH	None	1 Sun illumination produced by a 300 W xenon lamp	1.06 at 1.57 V_{RHE}	None	28
(Ti/Sn) $ZnFe_2O_4$ nanorod	1M KOH	Nickel-iron oxide	A solar simulator (91160, Oriel) with an filter (1 Sun)	~0.36 at 1.4 V_{RHE}	None	29
$CuFe_2O_4$	1M NaOH	None	A Xenon arc lamp (Newport 66921, 450 W), calibrated to AM 1.5G (1 sun)	0.5	None	30
$MgFe_2O_4$(H)	1M NaOH	Nickel-iron oxide	A450 W Muller Elektronik Xenon-arc lamp coupled with a KG-1 filter (Schott) (1 sun)	0.25	2.5% at 1.6 V_{RHE}	20
Co_3O_4/Fe_2O_3 p-n Nanorod Arrays	1M KOH	Co-Pi	A 300 W Xenon arc lamp equipped with an AM 1.5G filter (1 sun)	~3.4	23.0% at 1.23 V_{RHE}	31
Ta: Fe_2O_3@Fe_2O_3	1M KOH	NiFe$(OH)_x$	A solar simulator (91160, Oriel) equipped with an air mass (AM) 1.5 G filter (1 sun)	3.22 at 1.23 V_{RHE}	35.1% at 1.25 V_{RHE}	32
Fe_2O_3@$FeNbO_4$ nanorod	1M NaOH	$FeNiO_x$	1-sun condition by a solar simulator (91160, Oriel) with an AM 1.5 G filter.	~3.2	26% at 1.23 V_{RHE}	33
Ti: Si-Fe_2O_3	1M NaOH	$NiFeO_x$	AM 1.5 illumination (1 sun)	4 at 1.23 V_{RHE}	~20% at 1.23 V_{RHE}	34

Supplementary Table 2. Quality of raw materials used in the preparation of the conventional dual-phase interface films.

m (mg)	Zn: Fe=1:2.4	Zn: Fe=1:3	Zn: Fe=1:4	Zn: Fe=1:6
α-Fe_2O_3	44.1	37.5	30	21.5
$ZnFe_2O_4$	5.9	12.5	20	28.5

Supplementary Table 3. Solution concentrations for diffusion coefficient test.

	Zn	Fe	Zn: Fe=1:2	Zn: Fe=1:2.4	Zn: Fe=1:3	Zn: Fe=1:4	Zn: Fe=1:6
Concentration (mg/L)	3250	5600	3250	3250	3250	3250	3250
			5600	7280	8400	11200	16800

Supplementary Table 4. Photoelectrochemical impedance spectroscopic curves.

	α-Fe_2O_3	$ZnFe_2O_4$	The Turing interface (Zn: Fe=1:3)	The Turing interface (Zn: Fe=1:4)
R_s(Ω)	6	6	6	7
R_1(Ω)	33	31	30	33
CPE_1-T(F)	8.058E−10	6.031E−10	6.819E−10	6.758E-10
CPE_1-P	1.43	1.41	1.441	1.396
R_2(Ω)	3370	580	133	390
CPE_2-T(F)	1.7427E−5	5.095E−4	4.4533 E−5	4.0248E-5
CPE_2-P	0.6554	0.6133	0.84037	0.76713
R_3(Ω)	4597	700	460	858
CPE_3-T(F)	4.185E−5	4.6599E−4	2.8588E−3	1.046E-3
CPE_3-P	0.61906	0.6695	0.45178	0.82775

CPE_1-T, CPE_2-T and CPE_3-T are the constant phase elements representing the capacitance of the FTO/bulk sample interface, the capacitance of the bulk sample of the sample and the surface-state capacitance at the electrode/electrolyte interfaces, respectively. CPE_1-P, CPE_2-P and CPE_3-P represent the extent to which CPE_1-T, CPE_2-T and CPE_3-T deviate from the pure capacitance, respectively.

Supplementary References

[1] A. Hauch, R. Küngas, P. Blennow, A. B. Hansen, J. B. Hansen, B. V. Mathiesen, M. B. Mogensen. Recent advances in solid oxide cell technology for electrolysis. Science 370, eaba6118 (2020).

[2] M. Grätzel. Photoelectrochemical cells. Nature 414, 338–344 (2001).

[3] D.A. Grave, D.S. Ellis, Y. Piekner, M. Kölbach, H. Dotan, A. Kay, P. Schnell, R. Krol, F.F. Abdi, D. Friedrich, A. Rothschild. Extraction of mobile charge carrier photogeneration yield spectrum of ultrathin-film metal oxide photoanodes for solar water splitting. Nat. Mater. 20, 833–840 (2021).

[4] T. Fang, H.T. Huang, J.Y. Feng, Y.F. Hu, Y.S. Guo, S.Y. Zhang, Z.S. Li, Z.G. Zou. Exploring facile strategies for high-oxidation-state metal nitride synthesis: carbonate-assisted one-step synthesis of Ta_3N_5 films for solar water splitting. Sci. Bull. 63, 1404–1410 (2018).

[5] J.M. Yu, J. Lee, Y. Kim, J. Song, J. Oh, S.M. Lee, M. Jeong, Y. Kim, J.H. Kwak, S. Cho, C. Yang, J.W. Jang. High-performance and stable photoelectrochemical water splitting cell with organic-photoactive-layer based photoanode. Nat. Commun. 11, 5509 (2020).

[6] S. Kment, F. Riboni, S. Pausova, L. Wang, L.Y. Wang, H. Han, Z. Hubicka, J. Krysa, P. Schmuki, R. Zboril. Photoanodes based on TiO_2 and α-Fe_2O_3 for solar water splitting-superior role of 1D nanoarchitectures and of combined heterostructures. Chem. Soc. Rev. 46, 3716–3769 (2017).

[7] K. Sivula, F.L. Formal, M. Grätzel. Solar water splitting: progress using hematite (α-Fe_2O_3) photoelectrodes. ChemSusChem 4, 432–449 (2011).

[8] M.G. Walter, E.L. Warren, J.R. M. Kone, S.W. Boettcher, Q.X. Mi, E.A. Santori, N.S. Lewis. Solar water splitting cells. Chem. Rev. 110, 6446–6473 (2010).

[9] H. Dotan, K. Sivula, M. Grätzel, A. Rothschild, S.C. Warren. Probing the photoelectrochemical properties of hematite (α-Fe_2O_3) electrodes using hydrogen peroxide as a hole scavenger, Energy Environ. Sci. 2011, 4, 958–964 (2011).

[10] S.D. Tilley, M. Cornuz, K. Sivula, M. Grätzel. Light-induced water splitting with hematite: improved nanostructure and iridium oxide catalysis. Angew. Chem. Int. Ed.

49, 6405–6408 (2010).

[11] K. Sivula, R. Krol. Semiconducting materials for photoelectrochemical energy conversion. Nat. Rev. Mater. 1, 15010 (2016).

[12] A. Duret, M. Grätzel. Visible light-induced water oxidation on mesoscopic α-Fe_2O_3 Films Made by ultrasonic spray pyrolysis. J. Phys. Chem. B 109, 17184–17191 (2005).

[13] A. Kay, I. Cesar, M. Grätzel. New benchmark for water photooxidation by nanostructured α-Fe_2O_3 Films. J. Am. Chem. Soc. 128, 15714–15721 (2006).

[14] M.T. Mayer, C. Du, D.W. Wang. Hematite/Si nanowire dual-absorber system for photoelectrochemical water splitting at low applied potentials. J. Am. Chem. Soc. 134, 12406–12409 (2012).

[15] P. Peerakiatkhajohn, J.H. Yun, H.J. Chen, M.Q. Lyu, T. Butburee, L.Z. Wang. Stable hematite nanosheet photoanodes for enhanced photoelectrochemical water splitting. Adv. Mater. 28, 6405–6410 (2016).

[16] U. Bjorksten, J. Moser, M. Grätzel. Photoelectrochemical studies on nanocrystalline hematite films. Chem. Mater. 6, 858–863 (1994).

[17] X.D. Zhu, N. Guijarro, Y.P. Liu, P. Schouwink, R.A. Wells, F.L. Formal, S. Sun, C. Gao, K. Sivula. Spinel structural disorder influences solar-water-splitting performance of $ZnFe_2O_4$ nanorod photoanodes. Adv. Mater. 30, 1801612 (2018).

[18] R. Dillert, D.H. Taffa, M. Wark, T. Bredow, D.W. Bahnemann. Photoelectrochemical water splitting and photocatalytic hydrogen production using ferrites (MFe_2O_4) under visible light irradiation. APL Mater. 3, 104001 (2015).

[19] C.Y. Shang, C. Cao, D.Y. Yu, Y. Yan, Y.T. Lin, H.L. Li, T.T. Zheng, X.P. Yan, W.C. Yu, S.M. Zhou, J. Zeng. Electron correlations engineer catalytic activity of pyrochlore iridates for acidic water oxidation. Adv. Mater. 31, 1805104 (2019).

[20] N. Guijarro, P. Bornoz, M. Prevot, X. Yu, X. Zhu, M. Johnson, X. Jeanbourquin, F. Le Formal, K. Sivula. Evaluating spinel ferrites MFe_2O_4 (M = Cu, Mg, Zn) as photoanodes for solar water oxidation: prospects and limitations. Sustain. Energy Fuels 2, 103–117 (2018).

[21] R. A. Henning, P. Uredat, C. Simon, A. Bloesser, P. Cop, M. T. Elm, R. Marschall. Characterization of MFe_2O_4 (M = Mg, Zn) thin films prepared by pulsed laser

deposition for photoelectrochemical applications. J. Phys. Chem. C 123, 18240–18247 (2019).
[22] N. Zhang, Q. Quan, M.Y. Qi, Z.R. Tang, Y.J. Xu. Hierarchically tailorable double-array film hybrids with enhanced photocatalytic and photoelectrochemical performances. Appl. Catal. B 259, 118086 (2019).
[23] G.P. Hu, Z.S. Feng. Turing instability and pattern formation in a strongly coupled diffusive predator-prey system. Int. J. Bifurcation and Chaos. 30, 2030020-1–15 (2020).
[24] M. R. Garvie. Finite-difference schemes for reaction-diffusion equations modeling predator-prey interactions in MATLAB. Bull. Math. Biol. 69, 931–956 (2007).
[25] H. Miao, J. Yang, G.L. Peng, H.Q. Li, Y.F. Zhu. Enhancement of the degradation ability for organic pollutants via the synergistic effect of photoelectrocatalysis on a self-assembled perylene diimide (SA-PDI) thin film. Sci. Bull. 64, 896–903 (2019).
[26] G.L. Yang, Y.X. Li, H.W. Lin, X.H. Ren, D. Philo, Q. Wang, Y. He, F. Ichihara, S.Q. Luo, S.Y. Wang, J.H. Ye. Constructing chemical interaction between hematite and carbon nanosheets with single active sites for efficient photo-electrochemical water oxidation. Small Methods 4, 2000577 (2020).
[27] YP. Liu, M. Xia, L. Yao, M. Mensi, D. Ren, M. Grätzel, K. Sivula, N.Guijarro. Spectroelectrochemical and chemical evidence of surface passivation at zinc ferrite ($ZnFe_2O_4$) photoanodes for solar water oxidation. Adv. Funct. Mater. 31, 2010081 (2021).
[28] W.Q. Gao, R. Peng, Y.Y. Yang, X.L. Zhao, C. Cui, X.W. Su, W. Qin, Y. Dai, Y.D. Ma, H. Liu, Y.H. Sang. Electron spin polarization-enhanced photoinduced charge separation in ferromagnetic $ZnFe_2O_4$. ACS Energy Lett. 6, 21292137 (2021).
[29] J.H. Kim, J.H. Kim, J.H. Kim, Y.K. Kim, J.S. Lee. Intentional extrinsic doping into $ZnFe_2O_4$ nanorod photoanode for enhanced photoelectrochemical water splitting. Sol. RRL 4, 1900328 (2020).
[30] Y.P. Liu, F.L. Formal, F. Boudoire, L.Yao, K. Sivula, N. Guijarro. Insights into the interfacial carrier behaviour of copper ferrite ($CuFe_2O_4$) photoanodes for solar water oxidation. J. Mater. Chem. A 7, 1669–1677 (2019).

[31] S.S. Yi, B.R. Wulan, J.M. Yan, Q.Jiang. Highly efficient photoelectrochemical water Splitting: surface modification of cobalt-phosphate-loaded Co_3O_4/Fe_2O_3 p-n heterojunction nanorod arrays. Adv. Funct. Mater. 1801902 (2019).
[32] H.M. Zhang, D.F. Li, W.J. Byun, X.L. Wang, T.J. Shin, H.Y. Jeong, H.X. Han, C. Li, J.S. Lee. Gradient tantalum-doped hematite homojunction photoanode improves both photocurrents and turn-on voltage for solar water splitting. Nat. Commun. 11, 4622 (2020).
[33] H.M. Zhang, Y.K. Kim, H.Y. Jeong, J.S. Lee. A few atomic $FeNbO_4$ overlayers on hematite nanorods: microwave-induced high temperature phase for efficient photoelectrochemical water splitting. ACS Catal. 9, 1289–1297 (2019).
[34] J.Y. Park, K.Y. Yoon, T. Kim, H. Jang, M.J. Kwak, J.Y. Kim a, J.H. Jang. A highly transparent thin film hematite with multi-element dopability for an efficient unassisted water splitting system. Nano Energy 76, 105089 (2020).
