# Supplementary material for: Homogeneous solution assembled Turing structures with near zero strain semi-coherence interface
Source: Nat Commun. 2022 May 26;13:2942. doi: 10.1038/s41467-022-30574-3 (PMC9135718; doi:10.1038/s41467-022-30574-3)
Supplement: Supplementary file 3 — Description of Additional Supplementary Files [file 41467_2022_30574_MOESM3_ESM.pdf]

Description of Additional Supplementary Files

File Name: Supplementary Movie 1

Description: It shows the numerical simulations of the Turing structures.

File Name: Supplementary Movie 2

Description: It shows the shape evolution of droplets in a homogeneous solution (A is a solution with a large difference in diffusion coefficient, B is a solution with a small difference in diffusion coefficient).
