# Supplementary material for: Homogeneous solution assembled Turing structures with near zero strain semi-coherence interface
Source: Nat Commun. 2022 May 26;13:2942. doi: 10.1038/s41467-022-30574-3 (PMC9135718; doi:10.1038/s41467-022-30574-3)
Supplement: Supplementary file 6 — Solar Cells Reporting Summary [file 41467_2022_30574_MOESM6_ESM.pdf]

nature research

Corresponding author(s): Zhaosheng Li

Solar Cells Reporting Summary

Nature Research wishes to improve the reproducibility of the work that we publish. This form is intended for publication with all accepted papers reporting the characterization of photovoltaic devices and provides structure for consistency and transparency in reporting. Some list items might not apply to an individual manuscript, but all fields must be completed for clarity.

For further information on Nature Research policies, including our data availability policy, see Authors & Referees.

Experimental design

Please check: are the following details reported in the manuscript?

1. Dimensions

Item	Yes	No	Details
Area of the tested solar cells	☒	☐	The area of the tested solar cells is 0.09 cm2. See the Response to Referee's Letter.
Method used to determine the device area	☒	☐	A carbon back-electrode with an average area of 0.09 cm2 was deposited on the CsPbBr3 perovskite film by a doctor-blade coating method. See the Response to Referee's Letter.

2. Current-voltage characterization

Item	Yes	No	Details
Current density-voltage (J-V) plots in both forward and backward direction	☐	☒	Backward scan is applied for preliminary characterization, and overall efficiency is directly calculated from solar energy and discharge electrical energy. See the Response to Referee's Letter.
Voltage scan conditions<br>*For instance: scan direction, speed, dwell times*	☒	☐	The J-V plots were all recorded by the scans (a voltage step of 10 mV and a delay time of 50 ms) between -0.1 to 1.5V. See the Response to Referee's Letter.
Test environment<br>*For instance: characterization temperature, in air or in glove box*	☒	☐	The J-V plots were all recorded at ambient air. See the Response to Referee's Letter.
Protocol for preconditioning of the device before its characterization	☐	☒	No preconditioning was used.
Stability of the J-V characteristic<br>*Verified with time evolution of the maximum power point or with the photocurrent at maximum power point; see ref. 7 for details.*	☐	☒	Cells were stable during the measurements.

3. Hysteresis or any other unusual behaviour

Item	Yes	No	Details
Description of the unusual behaviour observed during the characterization	☐	☒	No unusual behaviour.
Related experimental data	☐	☒	No unusual behaviour.

4. Efficiency

Item	Yes	No	Details
External quantum efficiency (EQE) or incident photons to current efficiency (IPCE)	☐	☒	We mainly verify that Turing structure films improve the Power Conversion Efficiency. Thus, the focus of this work is not on solar cells, such information is unnecessary.
A comparison between the integrated response under the standard reference spectrum and the response measure under the simulator	☐	☒	The focus of this work is not about solar cells, such information is not requisite.
For tandem solar cells, the bias illumination and bias voltage used for each subcell	☐	☒	No tandem solar cells are fabricated in this work.

5. Calibration

Item	Yes	No	Details
Light source and reference cell or sensor used for the characterization	☒	☐	An Oriel 92251A-1000 sunlight simulator was used to provide the testing light AM 1.5G (100 mW cm-2). The light intensity was calibrated at 100 mW cm-2 by the standard reference of a Newport 91150V silicon cell before use. See the Response to Referee's Letter.

Confirmation that the reference cell was calibrated and certified	☒ Yes ☐ No	We confirm that the reference cell was calibrated and certified. See the Response to Referee's Letter.
Calculation of spectral mismatch between the reference cell and the devices under test	☐ Yes ☒ No	We did not calculate the mismatch between the reference cell and the devices under test, for this value is very small with our solar simulator and calibration reference cell.

6. Mask/aperture

Size of the mask/aperture used during testing	☐ Yes ☒ No	No mask is applied.
Variation of the measured short-circuit current density with the mask/aperture area	☐ Yes ☒ No	The focus of this work is not about solar cells, such experiment is not requisite.

7. Performance certification

Identity of the independent certification laboratory that confirmed the photovoltaic performance	☐ Yes ☒ No	The focus of this work is not on solar cells, such information is unnecessary.
A copy of any certificate(s) *Provide in Supplementary Information*	☐ Yes ☒ No	The focus of this work is not on solar cells, such information is unnecessary.

8. Statistics

Number of solar cells tested	☐ Yes ☒ No	We have tested a lot of perovskite solar cells during the experiment, but the focus of this work is not on solar cells, and such information is unnecessary.
Statistical analysis of the device performance	☐ Yes ☒ No	We conducted repeated experiments and tested a lot of perovskite solar cells during the experiment, but the focus of this work is not on solar cells, and such information is unnecessary.

9. Long-term stability analysis

Type of analysis, bias conditions and environmental conditions *For instance: illumination type, temperature, atmosphere humidity, encapsulation method, preconditioning temperature*	☐ Yes ☒ No	The test of the long-term stability analysis has little to do with our experiment.
